# Supplementary material for: Nitropyridines as 2π-Partners in 1,3-Dipolar Cycloadditions with N-Methyl Azomethine Ylide: An Easy Access to Condensed Pyrrolines
Source: Molecules. 2021 Sep 13;26(18):5547. doi: 10.3390/molecules26185547 (PMC8471275; doi:10.3390/molecules26185547)

Table of contents

NMR and HRMS spectra for **2a**	S2-S4
NMR and HRMS spectra for **2b**	S5-S8
NMR and HRMS spectra for **2c**	S9-S11
NMR and HRMS spectra for **2d**	S12-S14
NMR and HRMS spectra for **2e**	S15-S17
NMR and HRMS spectra for **2f**	S18-S20
NMR and HRMS spectra for **2g**	S21-S23
NMR and HRMS spectra for **2h**	S24-S26
NMR and HRMS spectra for **2i**	S27-S29
NMR and HRMS spectra for **2j**	S30-S32
NMR and HRMS spectra for **2k**	S33-S35
NMR and HRMS spectra for **2l**	S36-S38
NMR and HRMS spectra for **2m**	S39-S42
NMR and HRMS spectra for **2n**	S43-S45
NMR and HRMS spectra for **2o**	S46-S48
NMR and HRMS spectra for **2p**	S49-S52
NMR and HRMS spectra for **2q**	S53-S55
NMR and HRMS spectra for **4a**	S56-S58
NMR and HRMS spectra for **4b**	S59-S61
NMR and HRMS spectra for **4c**	S62-S64
NMR and HRMS spectra for **4d**	S65-S67
NMR and HRMS spectra for **4e**	S68-S70
NMR and HRMS spectra for **4f**	S71-S73
NMR and HRMS spectra for **4g**	S74-S76
NMR and HRMS spectra for **4h**	S77-S79
NMR and HRMS spectra for **4i**	S80-S82
NMR and HRMS spectra for **4j**	S83-S85
NMR and HRMS spectra for **5**	S86-S92

© Zelinsky Institute of Organic Chemistry, Moscow; Bruker AM300 SF=300.13 MHz {1H} SI=32K SW=6010 O1=2401 PW=9.0 AQ=1.352 RD=0.00 NS=1 SR=−1.41 TE=297K 8 October 2020 Opr: Daeva E.D.; Solv: CDCl3;

The Best Applied NMR! On−Line: "http://nmr.ioc.ac.ru:8080"

/LPIK AF−363

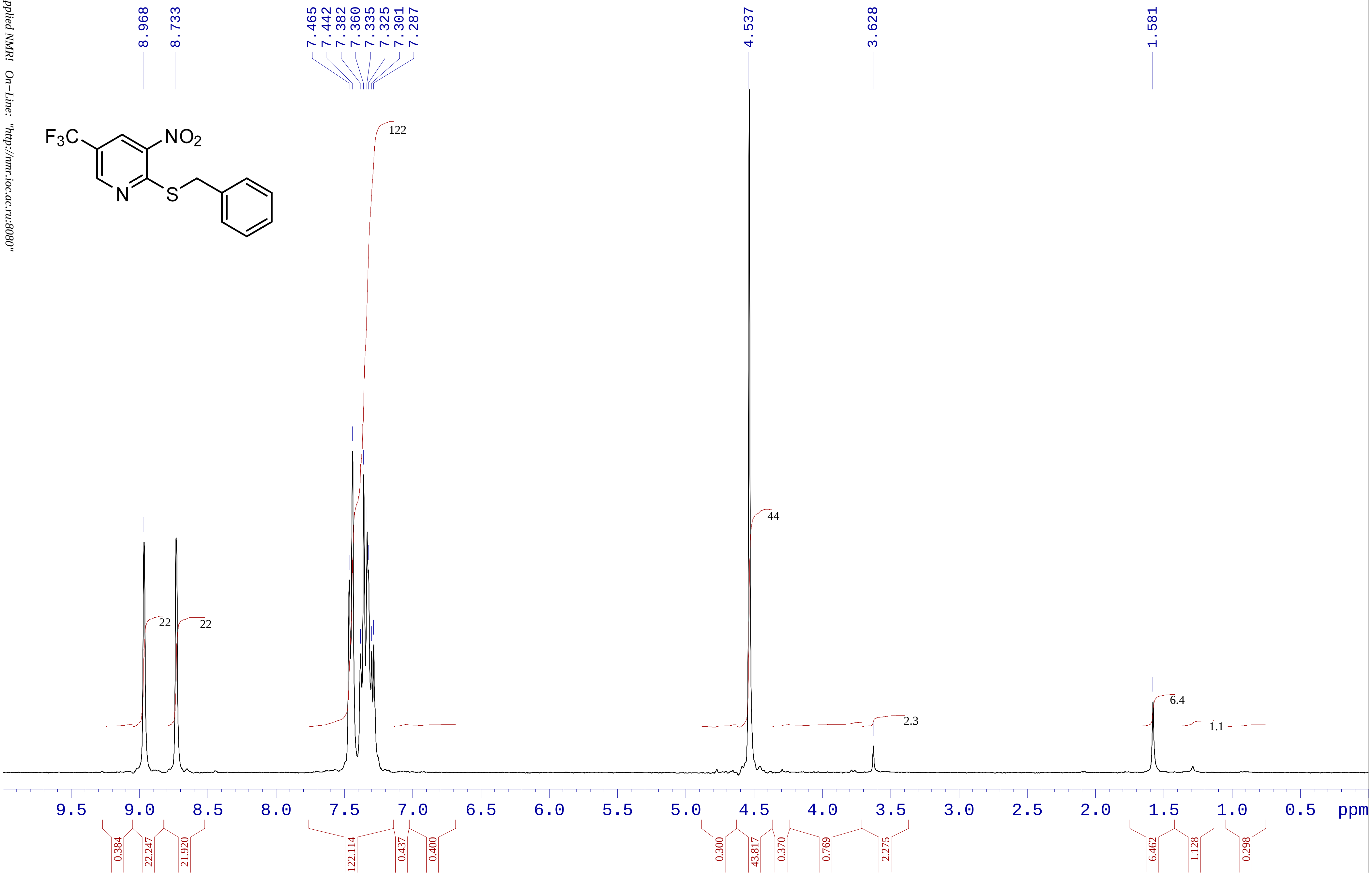

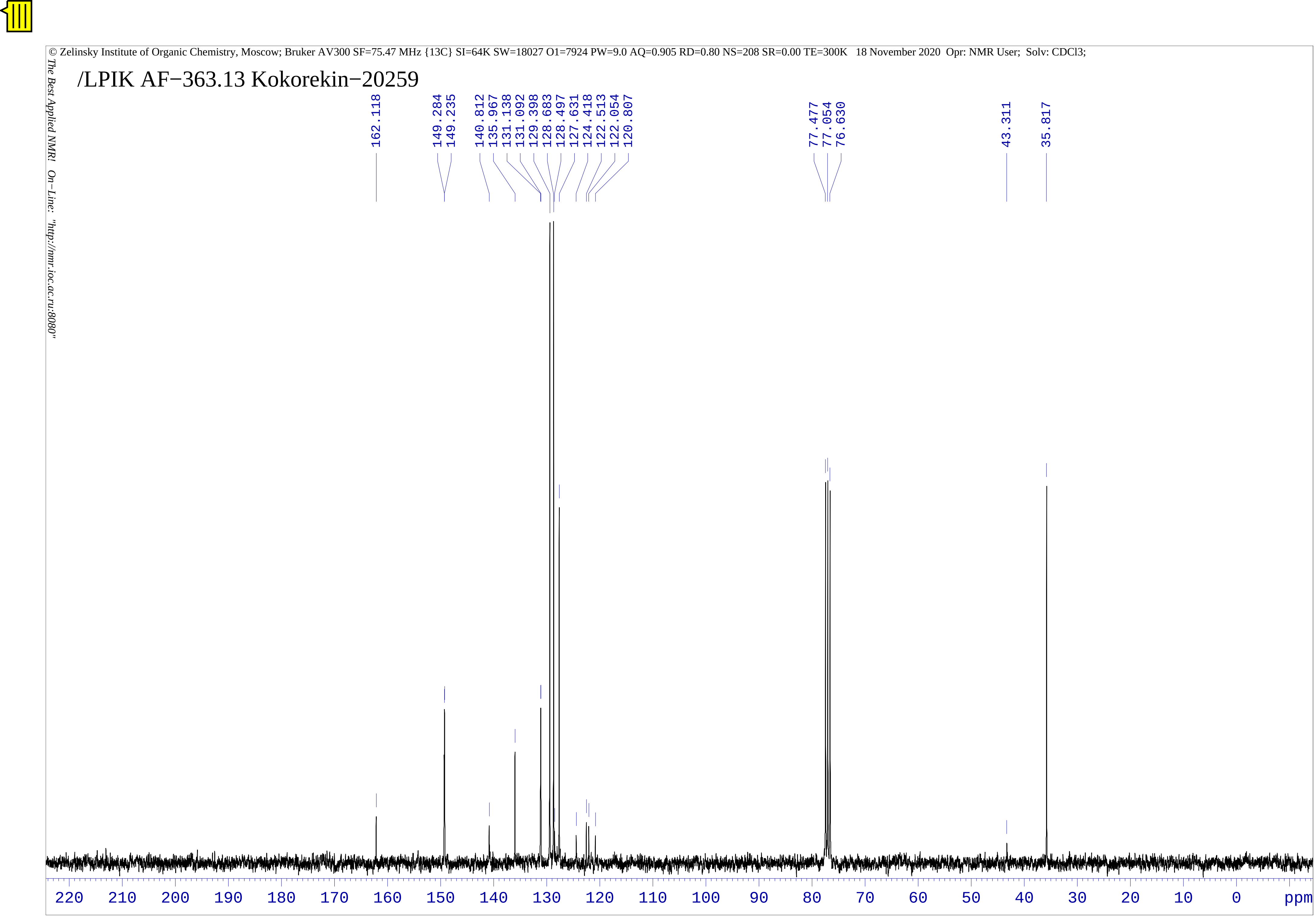
© Zelinsky Institute of Organic Chemistry, Moscow; Bruker AV300 SF=75.47 MHz {13C} SI=64K SW=18027 O1=7924 PW=9.0 AQ=0.905 RD=0.80 NS=208 SR=0.00 TE=300K 18 November 2020 Opr: NMR User; Solv: CDCl3;
The Best Applied NMR! On−Line: "http://nmr.ioc.ac.ru:8080"
/LPIK AF−363.13 Kokorekin−20259
162.118
149.284
149.235
140.812
135.967
131.138
131.092
129.398
128.683
128.497
127.631
124.418
122.513
122.054
120.807
77.477
77.054
76.630
43.311
35.817
220 210 200 190 180 170 160 150 140 130 120 110 100 90 80 70 60 50 40 30 20 10 0 ppm

Display Report

Analysis Info

Analysis Name	D:\Data\Kolotyrkina\2021\Bastrakov\0210024.d	Acquisition Date	10.02.2021 20:12:35
Method	tune_50-1600.m	Operator	BDAL@DE
Sample Name	/LPIK AF-363	Instrument / Ser#	micrOTOF 10248
Comment	C13H9F3N2O2S mH 315.0409 calibrant added CH3OH		

Acquisition Parameter

Source Type	ESI	Ion Polarity	Positive	Set Nebulizer	1.0 Bar
Focus	Not active			Set Dry Heater	200 °C
Scan Begin	50 m/z	Set Capillary	4500 V	Set Dry Gas	4.0 l/min
Scan End	1600 m/z	Set End Plate Offset	-500 V	Set Divert Valve	Waste

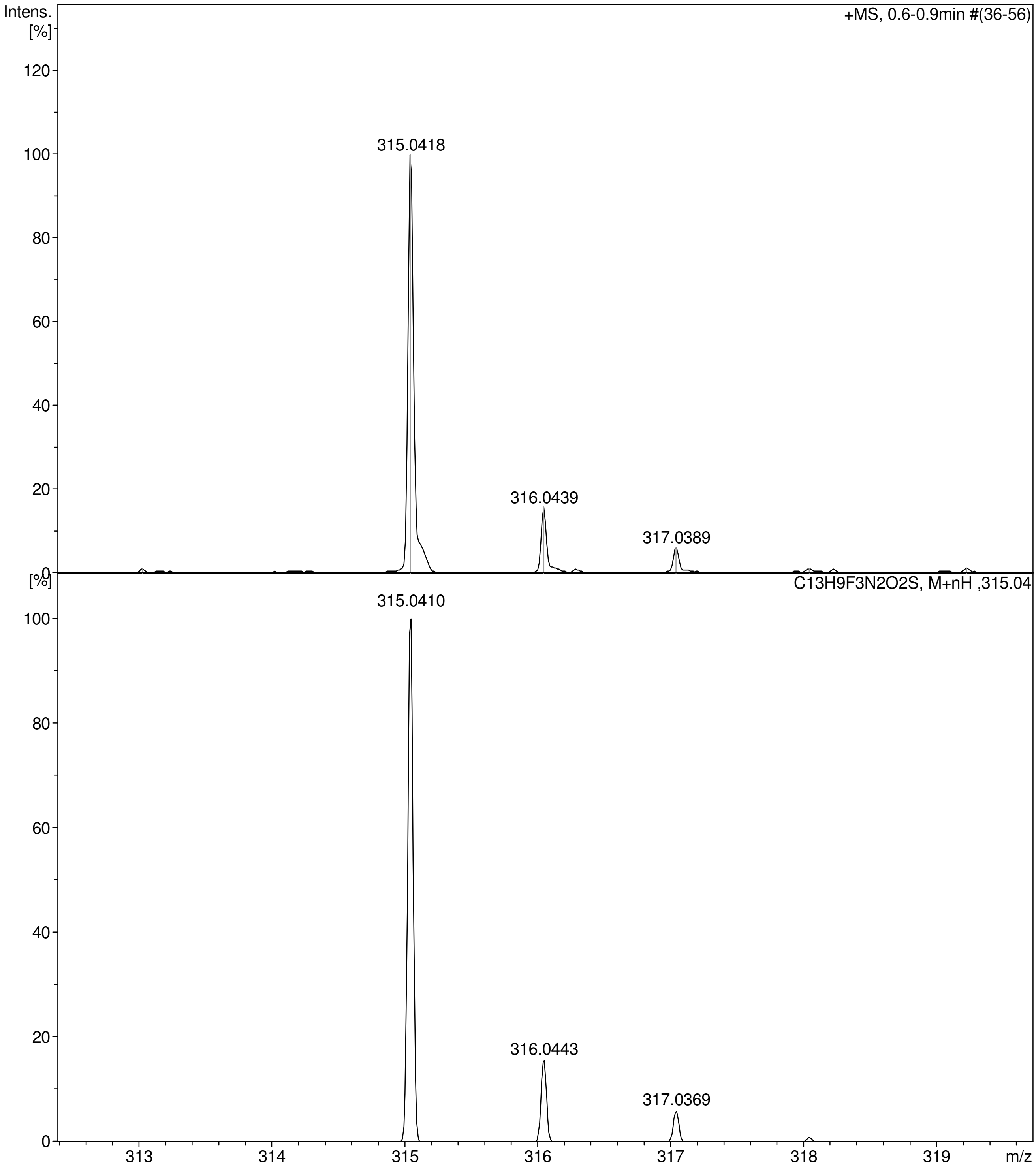

© Zelinsky Institute of Organic Chemistry, Moscow; Bruker AM300 SF=300.13 MHz {1H} SI=32K SW=6010 O1=2401 PW=9.0 AQ=1.352 RD=0.00 NS=1 SR=−1.41 TE=296K 8 October 2020 Opr: Daeva E.D.; Solv: CDCl3;

/LPIK AF−364

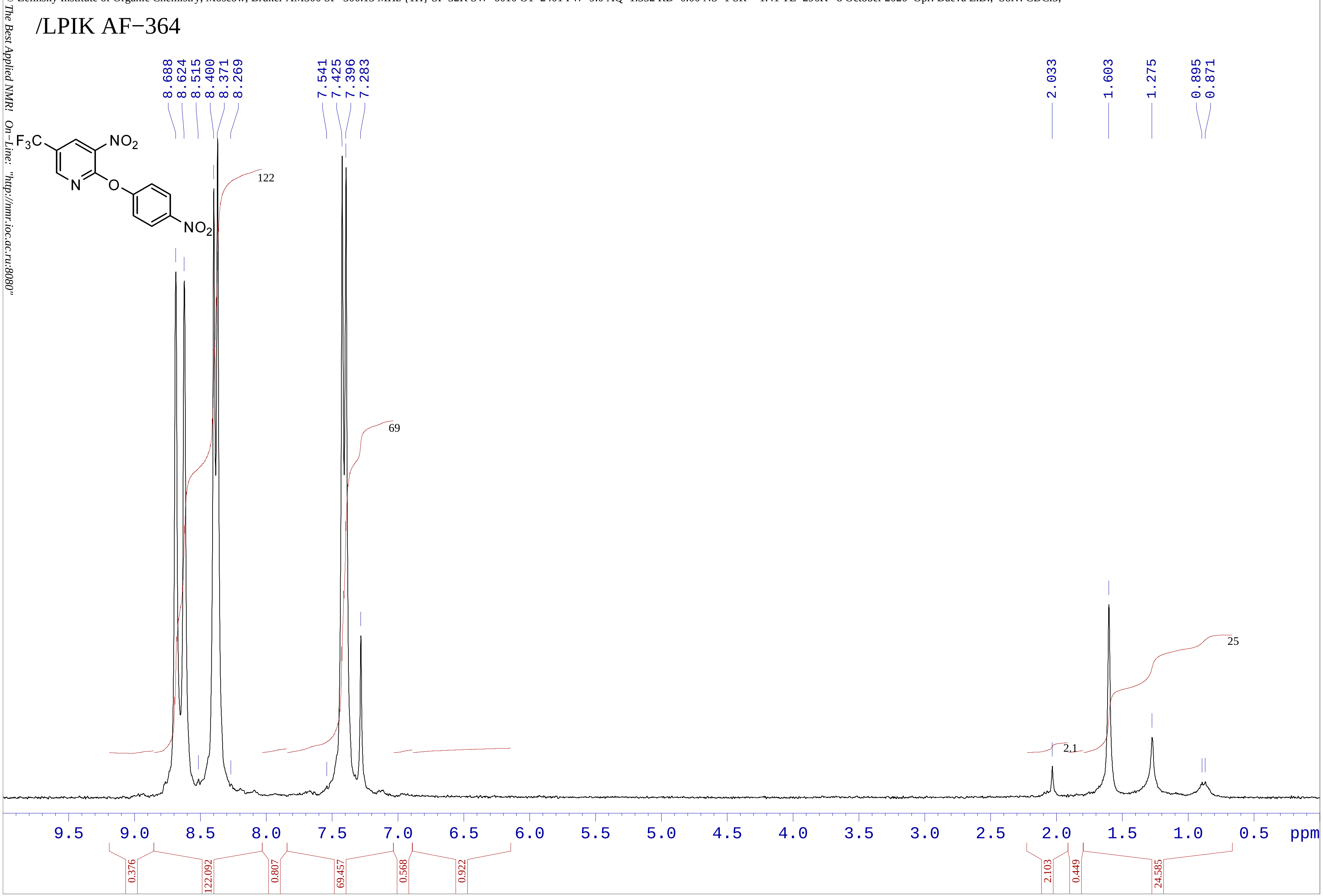

© Zelinsky Institute of Organic Chemistry, Moscow; Bruker AV300 SF=75.47 MHz {13C} SI=64K SW=18027 O1=7924 PW=9.0 AQ=0.905 RD=0.80 NS=235 SR=0.00 TE=300K 18 November 2020 Opr: NMR User; Solv: CDCl3;

The Best Applied NMR! On−Line: "http://nmr.ioc.ac.ru:8080"

/LPIK AF−364.13 Kokorekin−20259

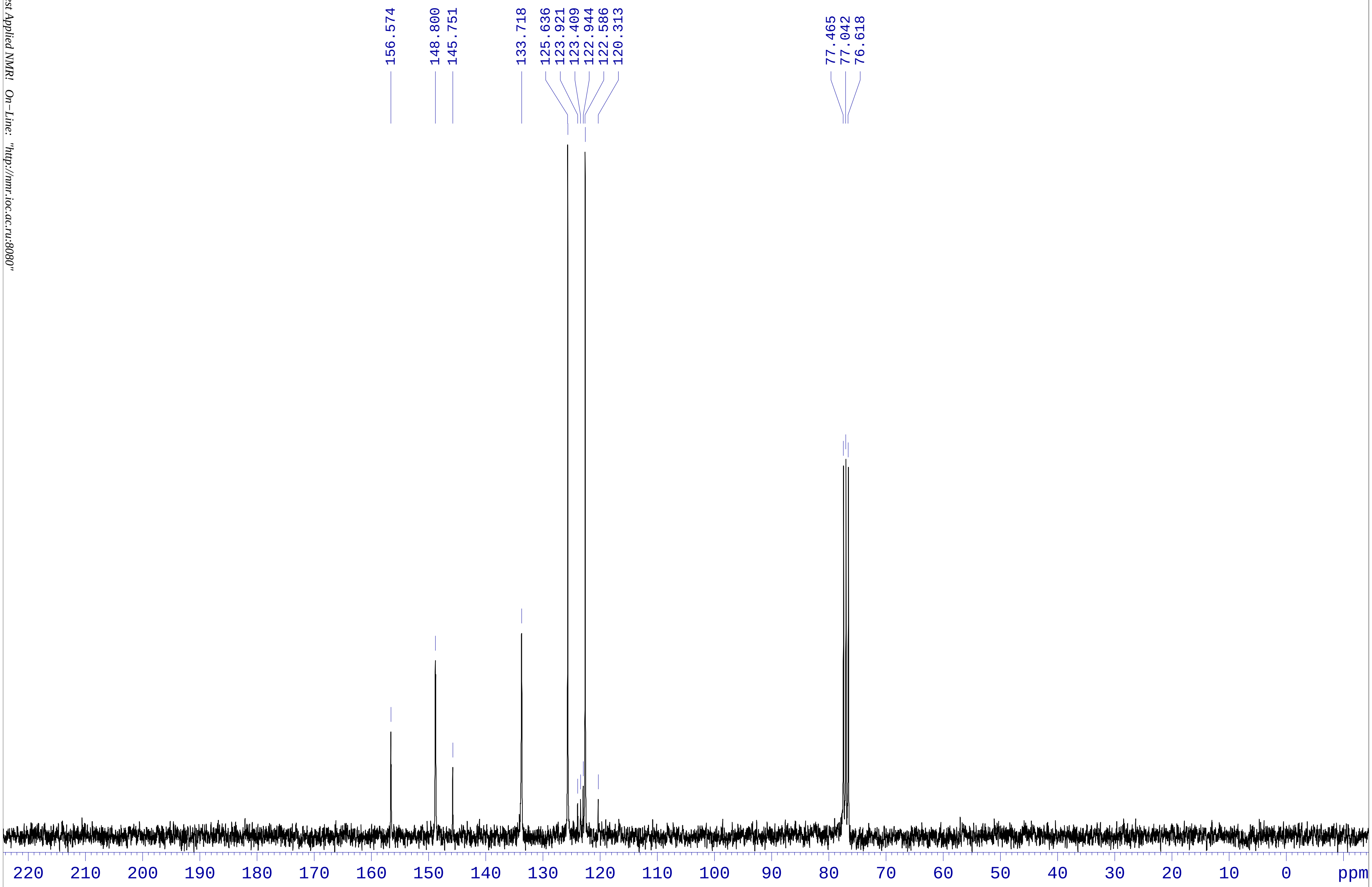

Display Report

Analysis Info

Analysis Name	D:\Data\Kolotyrkina\2021\Bastrakov\0211019.d	Acquisition Date	11.02.2021 12:34:23
Method	tune_50-1600.m	Operator	BDAL@DE
Sample Name	/LPIK AF-364	Instrument / Ser#	micrOTOF 10248
Comment	C12H6F3N3O5 mH 330.0332 alibrant added CH3OH		

Acquisition Parameter

Source Type	ESI	Ion Polarity	Positive	Set Nebulizer	1.0 Bar
Focus	Not active			Set Dry Heater	200 °C
Scan Begin	50 m/z	Set Capillary	4500 V	Set Dry Gas	4.0 l/min
Scan End	1600 m/z	Set End Plate Offset	-500 V	Set Divert Valve	Waste

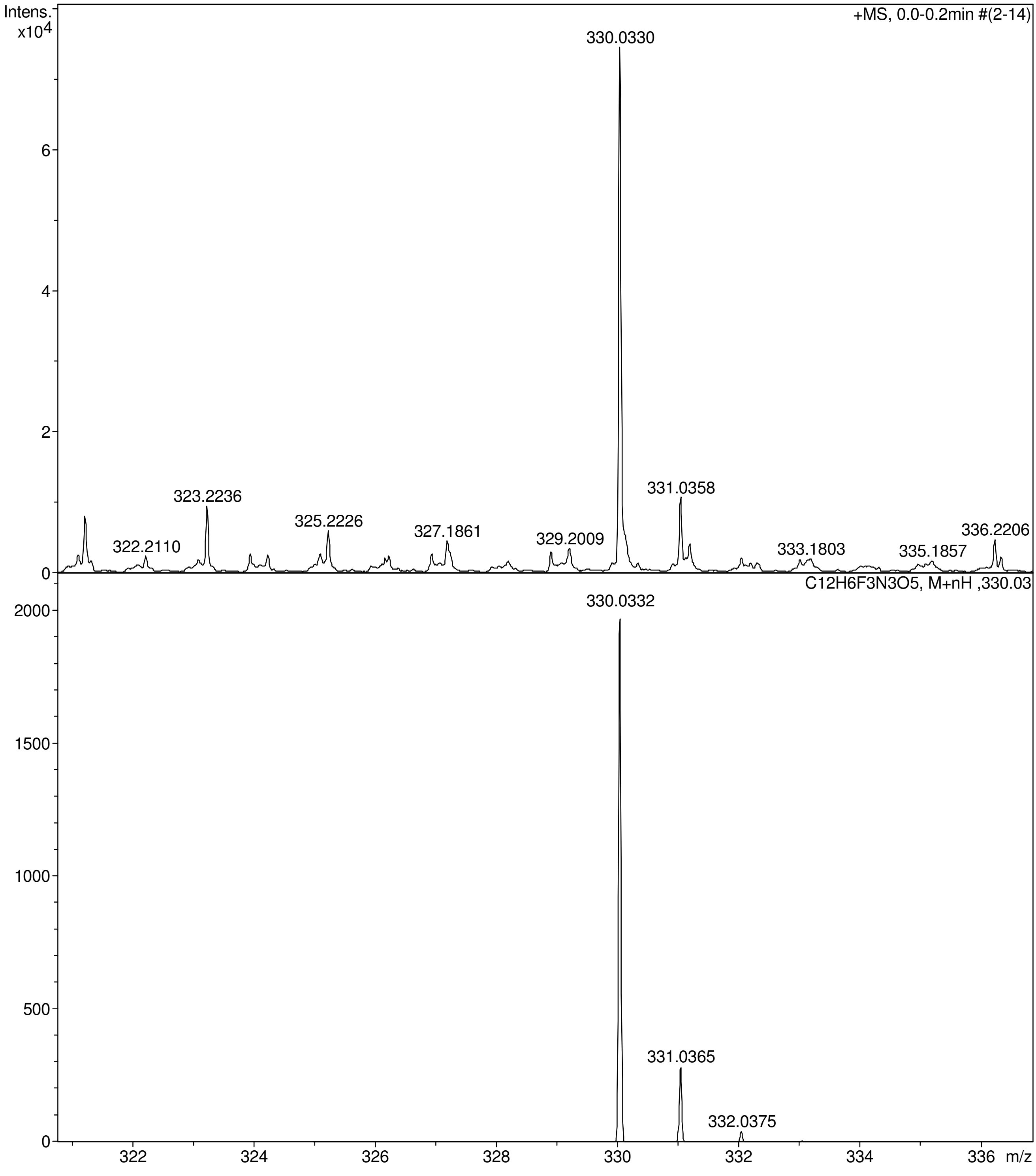

Display Report

Analysis Info

Analysis Name	D:\Data\Kolotyrkina\2021\Bastrakov\0211020.d	Acquisition Date	11.02.2021 12:40:15
Method	tune_50-1600.m	Operator	BDAL@DE
Sample Name	/LPIK AF-364	Instrument / Ser#	micrOTOF 10248
Comment	C13H9ClN2O5 mH 309.0272 alibrant added CH3OH		

Acquisition Parameter

Source Type	ESI	Ion Polarity	Positive	Set Nebulizer	1.0 Bar
Focus	Not active			Set Dry Heater	200 °C
Scan Begin	50 m/z	Set Capillary	4500 V	Set Dry Gas	4.0 l/min
Scan End	1600 m/z	Set End Plate Offset	-500 V	Set Divert Valve	Waste

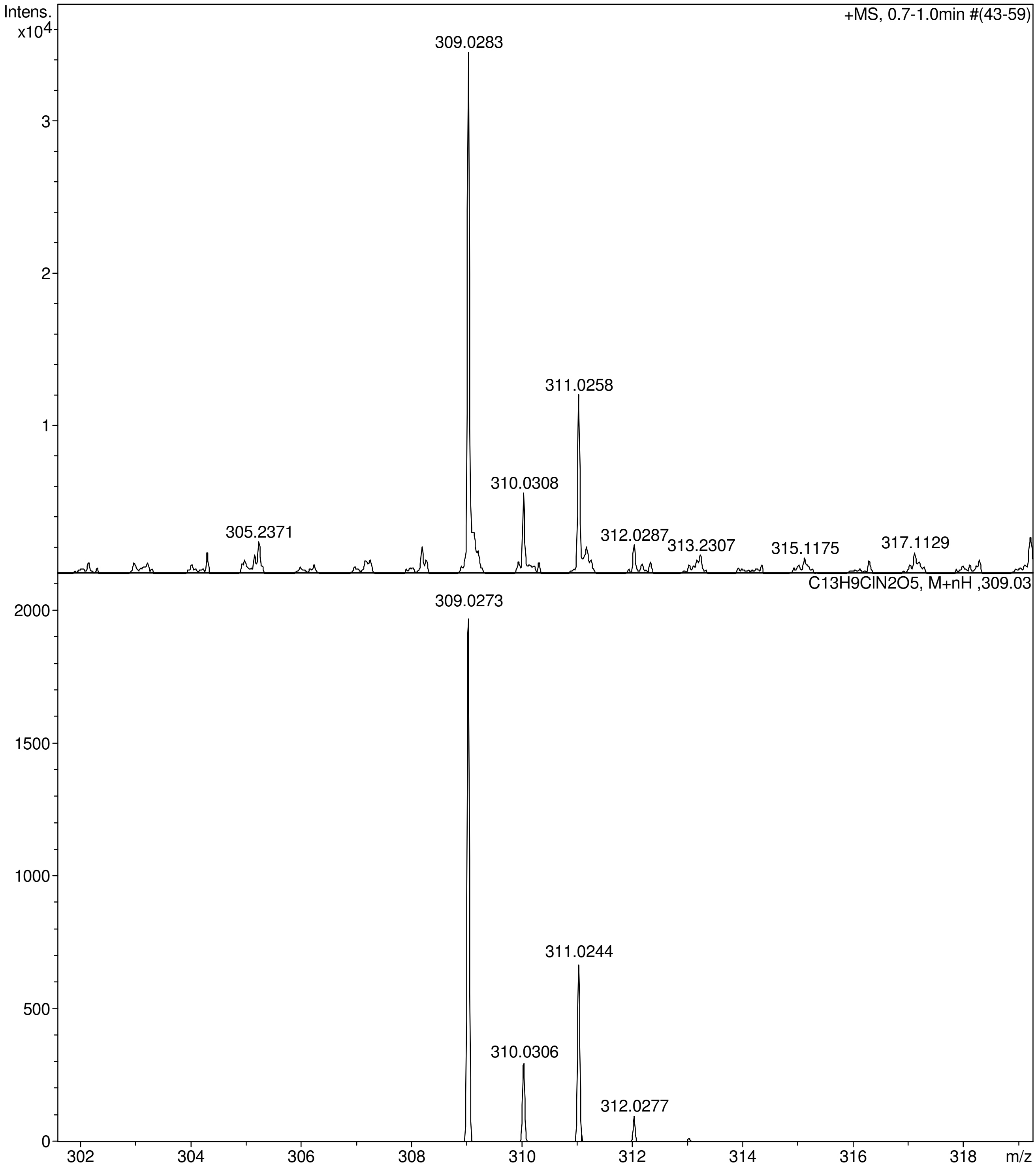

© Zelinsky Institute of Organic Chemistry, Moscow; Bruker AM300 SF=300.13 MHz {1H} SI=32K SW=6010 O1=2401 PW=9.0 AQ=1.352 RD=0.00 NS=1 SR=−1.41 TE=296K 14 October 2020 Opr: Daeva E.D.; Solv: CDCl3;

The Best Applied NMR! On−Line: "http://nmr.ioc.ac.ru:8080"

/LPIK AF−369

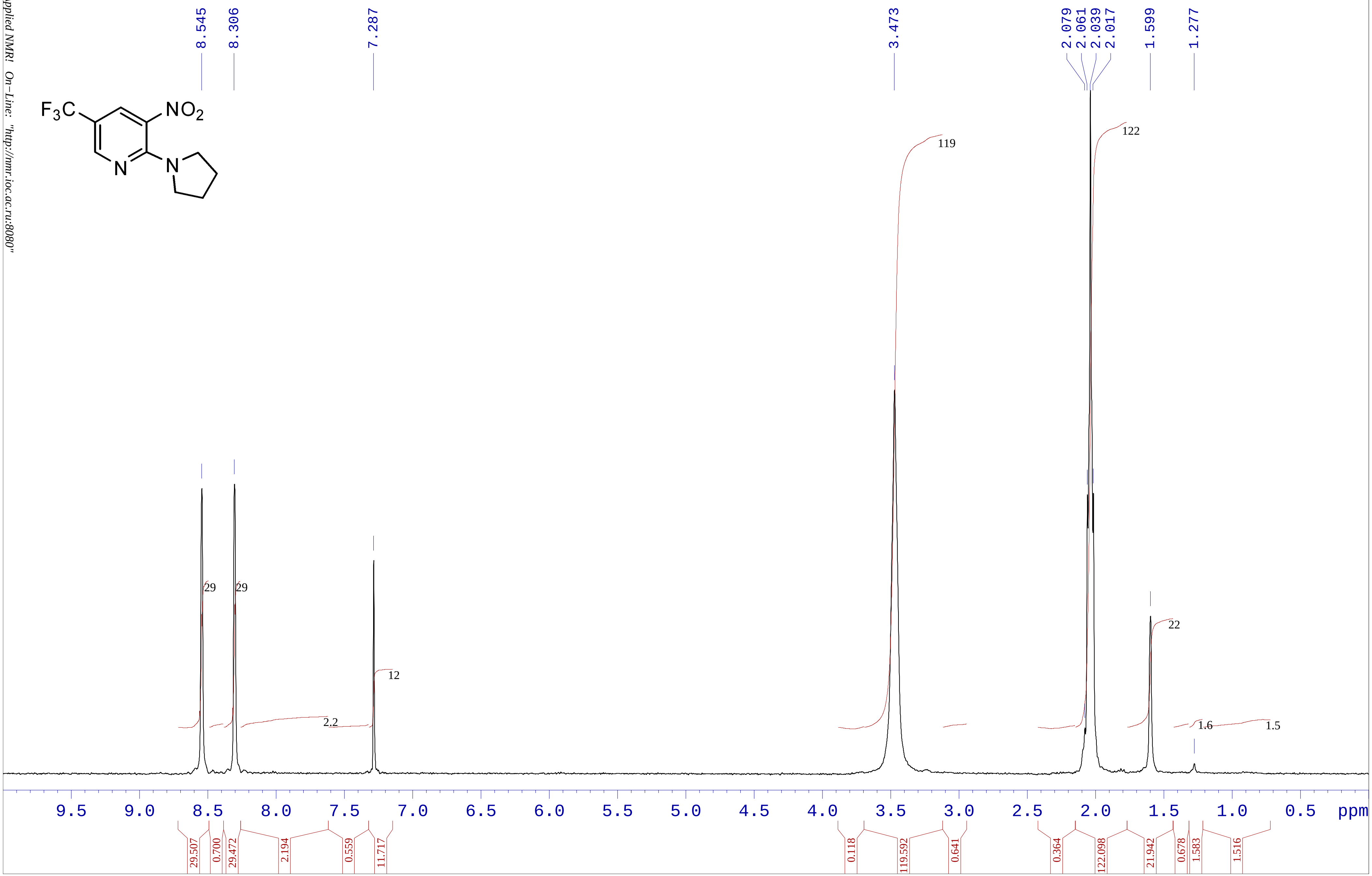

© Zelinsky Institute of Organic Chemistry, Moscow; Bruker AV300 SF=75.47 MHz {13C} SI=64K SW=18027 O1=7924 PW=9.0 AQ=0.905 RD=0.80 NS=194 SR=0.00 TE=300K 17 November 2020 Opr: NMR User; Solv: CDCl3;

The Best Applied NMR! On−Line: "http://nmr.ioc.ac.ru:8080"

/LPIK AF−369.13 Kokorekin−20259

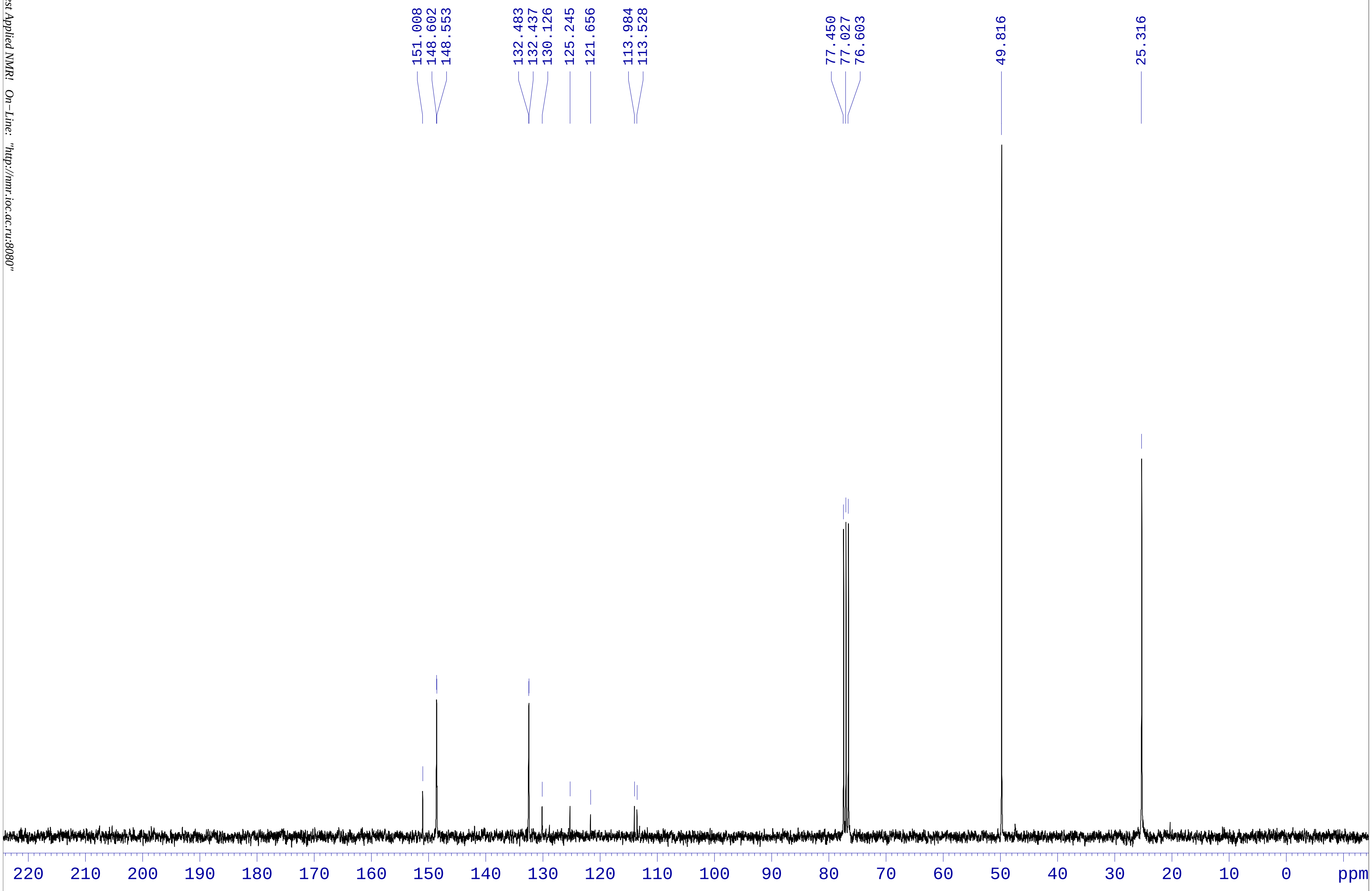

Display Report

Analysis Info

Analysis Name	D:\Data\Kolotyrkina\2021\Bastrakov\0210025.d	Acquisition Date	10.02.2021 20:24:40
Method	tune_50-1600.m	Operator	BDAL@DE
Sample Name	/LPIK AF-369	Instrument / Ser#	micrOTOF 10248
Comment	C10H10F3N3O2 mH 262.0797 calibrant added CH3CN		

Acquisition Parameter

Source Type	ESI	Ion Polarity	Positive	Set Nebulizer	1.0 Bar
Focus	Not active			Set Dry Heater	200 °C
Scan Begin	50 m/z	Set Capillary	4500 V	Set Dry Gas	4.0 l/min
Scan End	1600 m/z	Set End Plate Offset	-500 V	Set Divert Valve	Waste

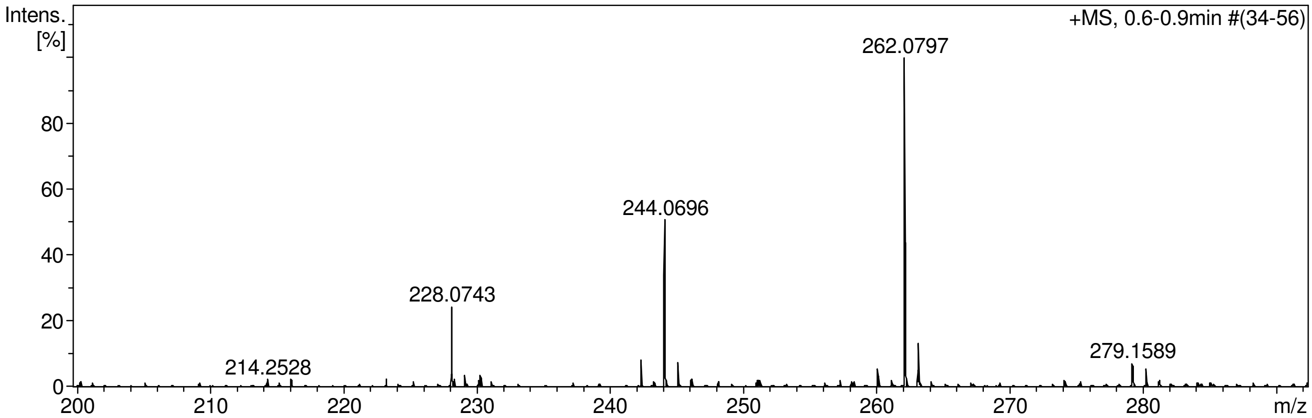

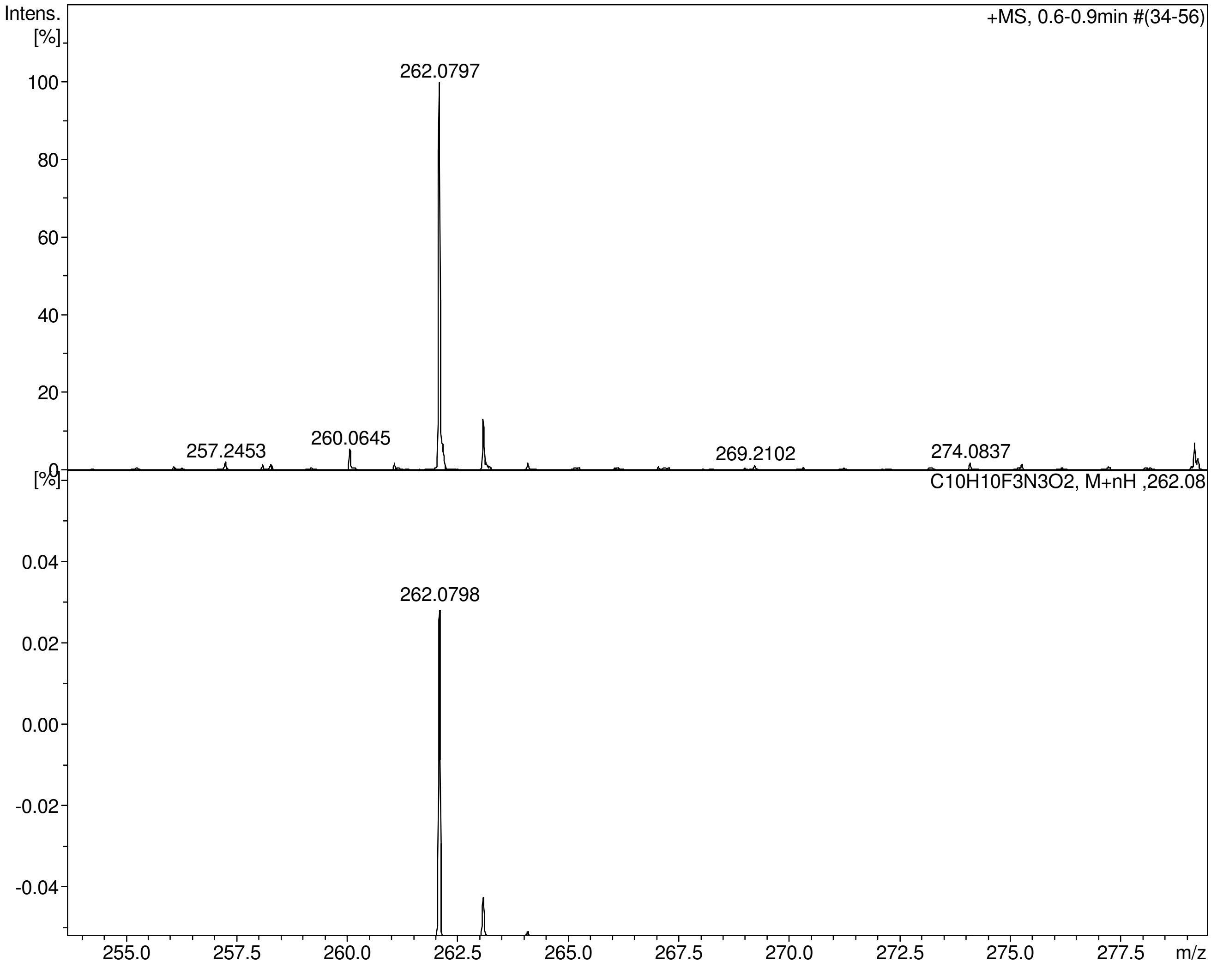

© Zelinsky Institute of Organic Chemistry, Moscow; Bruker AM300 SF=300.13 MHz {1H} SI=16K SW=6010 O1=2401 PW=9.0 AQ=1.352 RD=0.00 NS=1 SR=4.45 TE=301K 10 November 2020 Opr: Daeva E.D.; Solv: CDCl3;

The Best Applied NMR! On-Line: "http://nmr.ioc.ac.ru:8080"

/LPIK AF−386

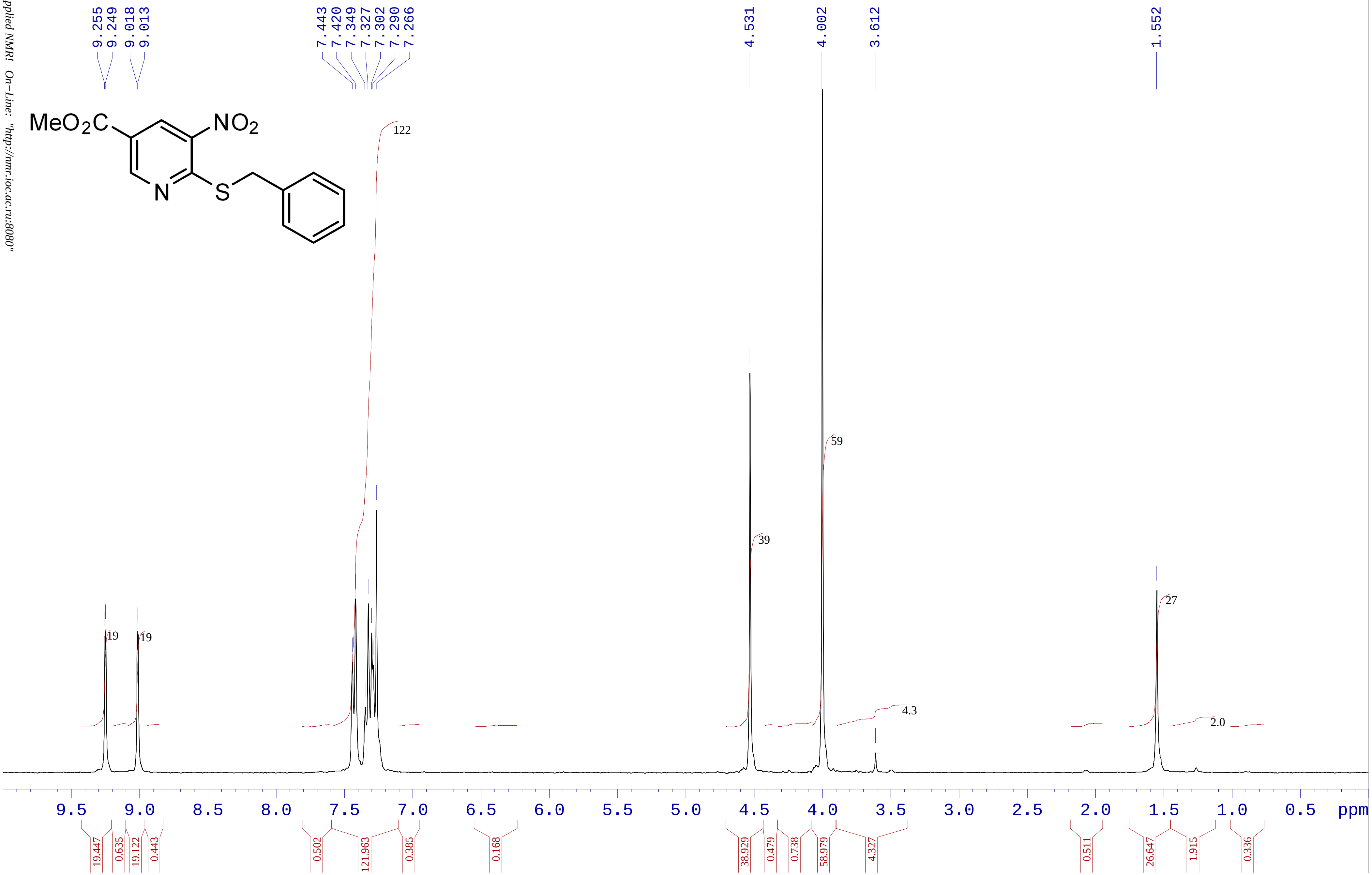

© Zelinsky Institute of Organic Chemistry, Moscow; Bruker AV300 SF=75.47 MHz {13C} SI=64K SW=18027 O1=7924 PW=9.0 AQ=0.905 RD=0.80 NS=58 SR=0.00 TE=300K 17 November 2020 Opr: NMR User; Solv: CDCl3;

The Best Applied NMR! On−Line: "http://nmr.ioc.ac.ru:8080"

/LPIK AF−386.13 Kokorekin−20259

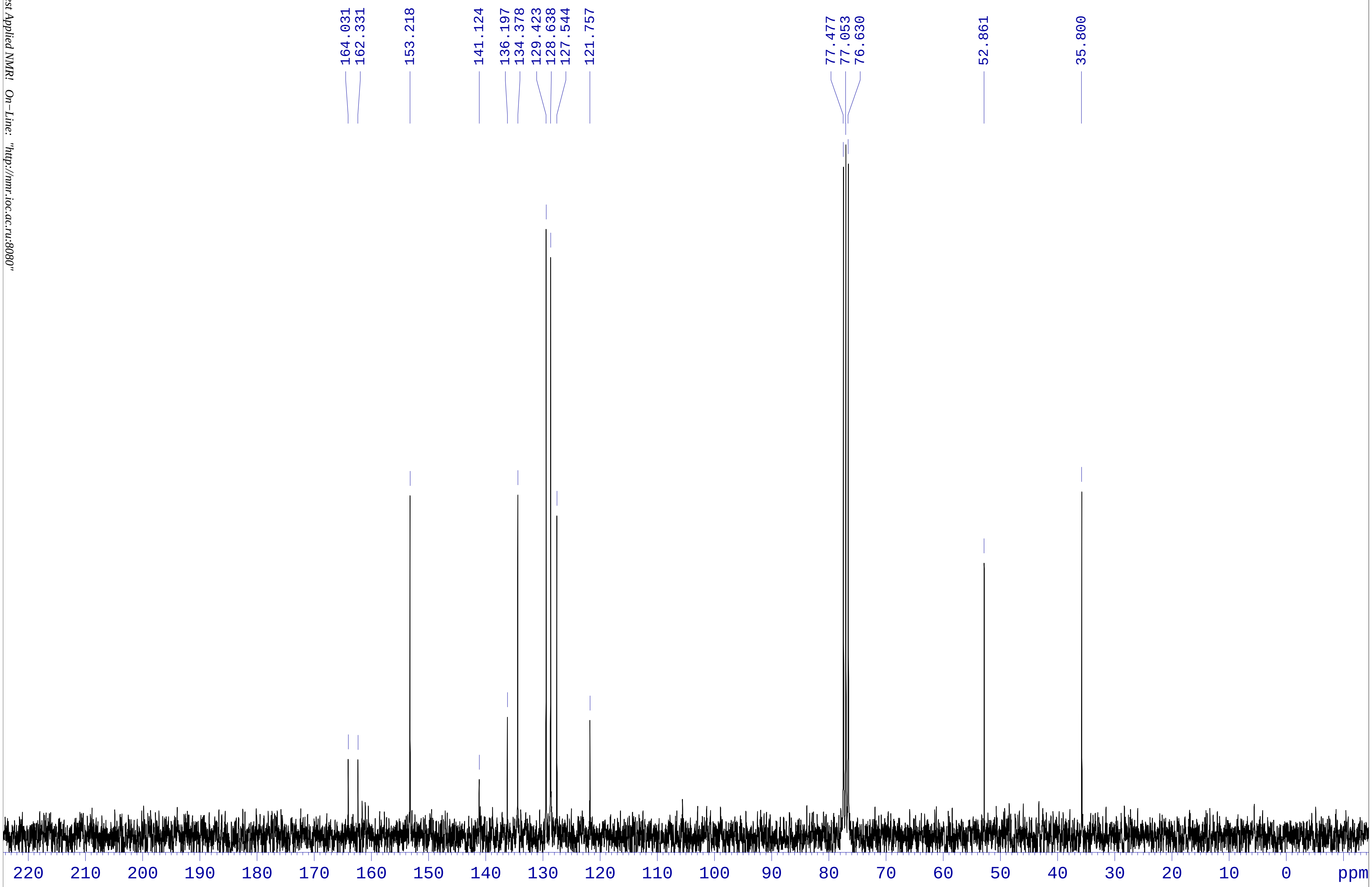

Display Report

Analysis Info

Analysis Name	D:\Data\Kolotyrkina\2021\Bastrakov\0210027.d	Acquisition Date	10.02.2021 20:41:38
Method	tune_50-1600.m	Operator	BDAL@DE
Sample Name	/LPIK AF-386	Instrument / Ser#	micrOTOF 10248
Comment	C14H12N2O4S mH 305.050 calibrant added CH3CN		

Acquisition Parameter

Source Type	ESI	Ion Polarity	Positive	Set Nebulizer	1.0 Bar
Focus	Not active			Set Dry Heater	200 °C
Scan Begin	50 m/z	Set Capillary	4500 V	Set Dry Gas	4.0 l/min
Scan End	1600 m/z	Set End Plate Offset	-500 V	Set Divert Valve	Waste

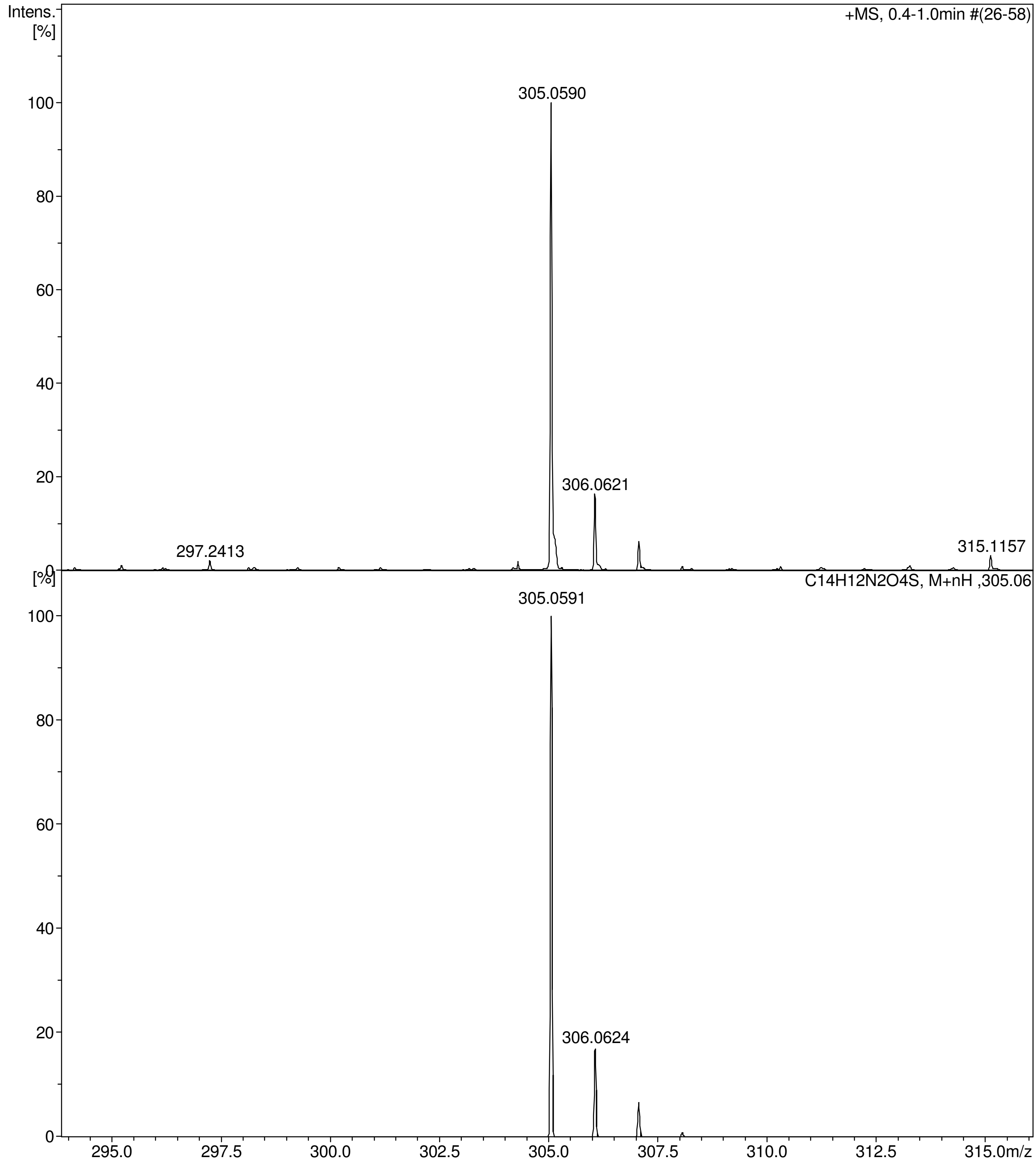

© Zelinsky Institute of Organic Chemistry, Moscow; Bruker AM300 SF=300.13 MHz {1H} SI=16K SW=6010 O1=2401 PW=9.0 AQ=1.352 RD=0.00 NS=1 SR=4.45 TE=301K 12 November 2020 Opr: Daeva E.D.; Solv: CDCl3;

The Best Applied NMR! On−Line: "http://nmr.ioc.ac.ru:8080"

/LPIK AF−389

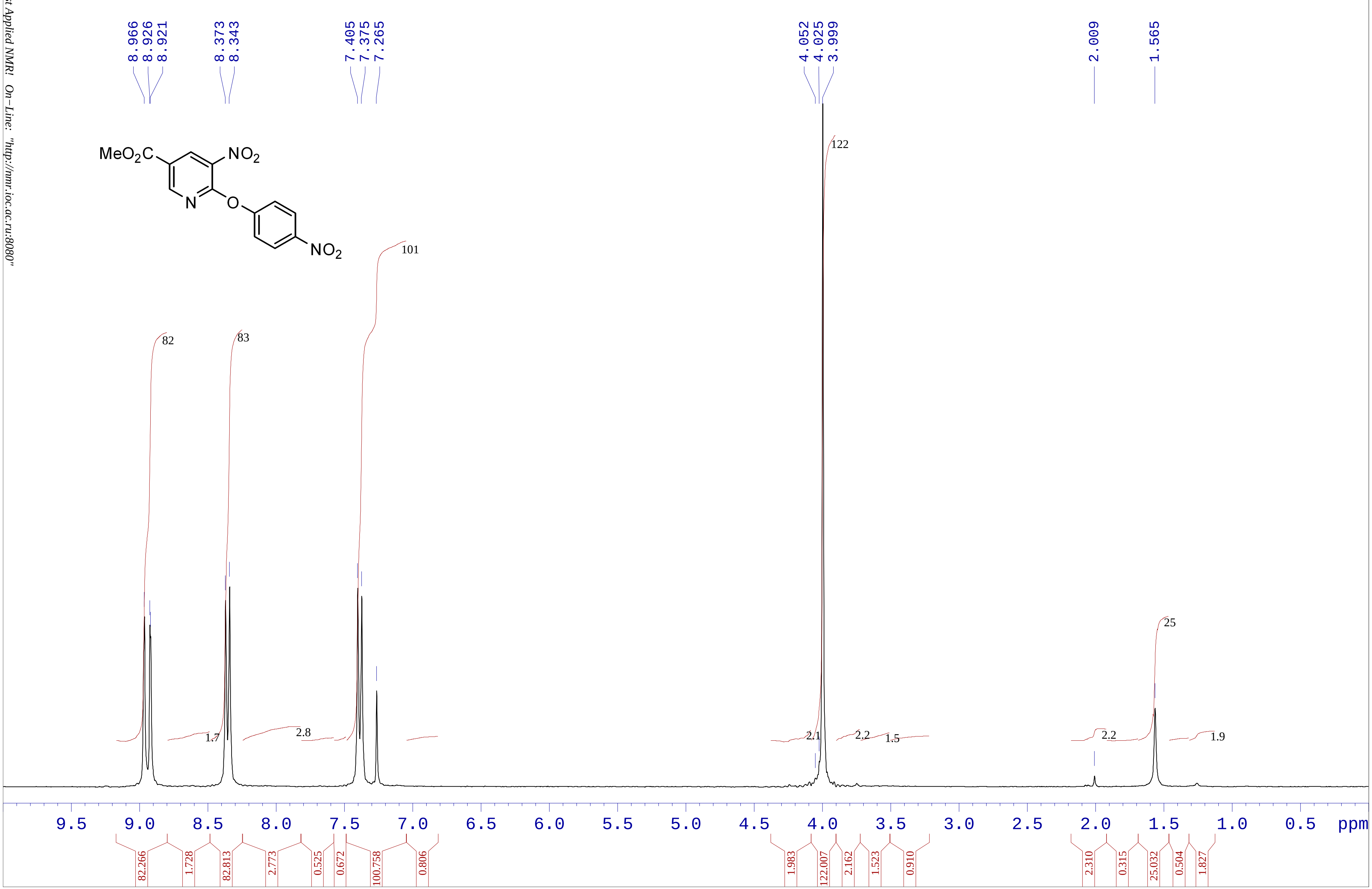

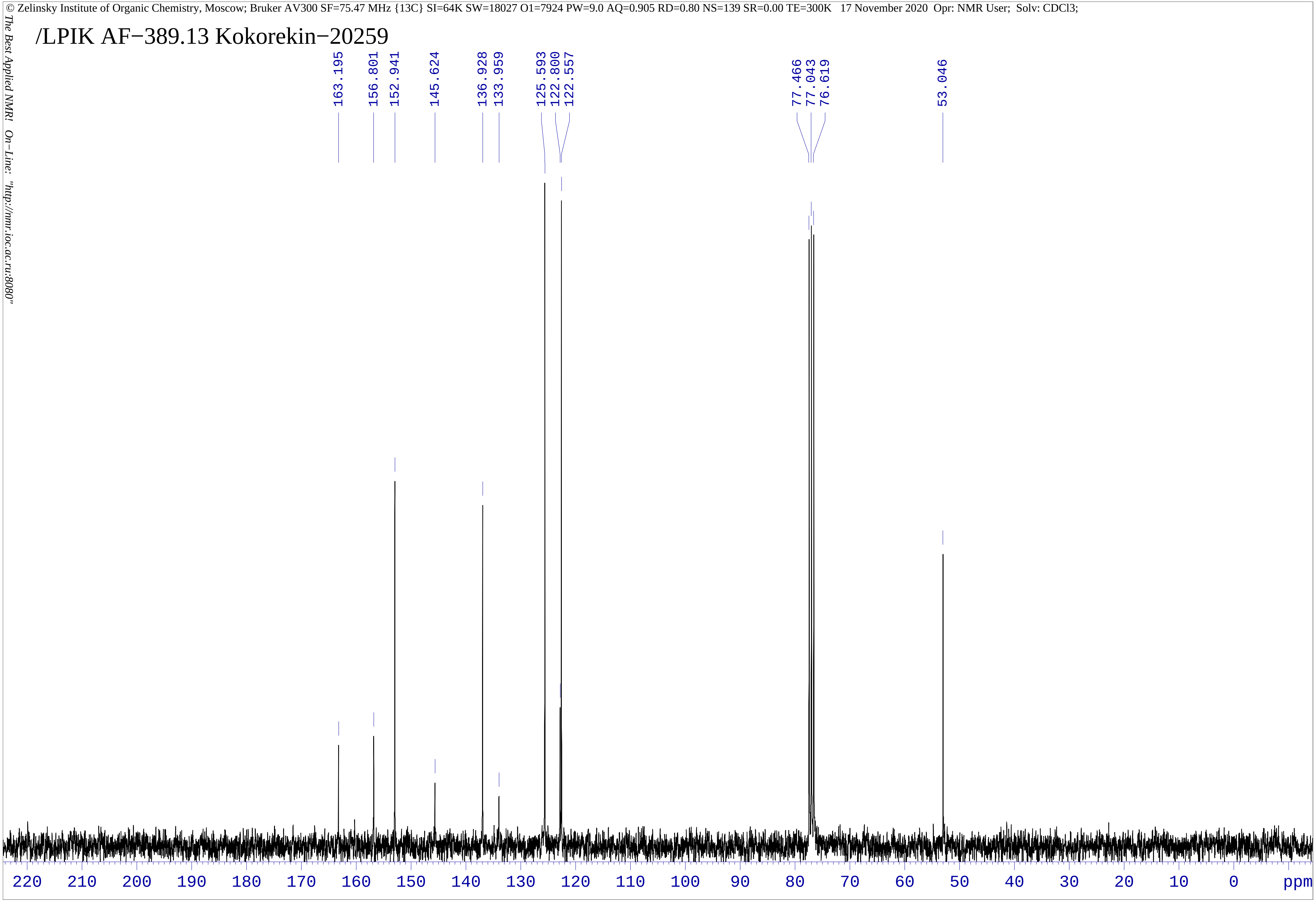
© Zelinsky Institute of Organic Chemistry, Moscow; Bruker AV300 SF=75.47 MHz {13C} SI=64K SW=18027 O1=7924 PW=9.0 AQ=0.905 RD=0.80 NS=139 SR=0.00 TE=300K 17 November 2020 Opr: NMR User; Solv: CDCl3;
The Best Applied NMR! On−Line: "http://nmr.ioc.ac.ru:8080"
/LPIK AF−389.13 Kokorekin−20259
163.195
156.801
152.941
145.624
136.928
133.959
125.593
122.800
122.557
77.466
77.043
76.619
53.046
220
210
200
190
180
170
160
150
140
130
120
110
100
90
80
70
60
50
40
30
20
10
0
ppm

Display Report

Analysis Info

Analysis Name	D:\Data\Kolotyrkina\2021\Bastrakov\0210028.d	Acquisition Date	10.02.2021 20:47:05
Method	tune_50-1600.m	Operator	BDAL@DE
Sample Name	/LPIK AF-389	Instrument / Ser#	micrOTOF 10248
Comment	C13H9N3O7 mH 320.0543calibrant added CH3CN		

Acquisition Parameter

Source Type	ESI	Ion Polarity	Positive	Set Nebulizer	1.0 Bar
Focus	Not active			Set Dry Heater	200 °C
Scan Begin	50 m/z	Set Capillary	4500 V	Set Dry Gas	4.0 l/min
Scan End	1600 m/z	Set End Plate Offset	-500 V	Set Divert Valve	Waste

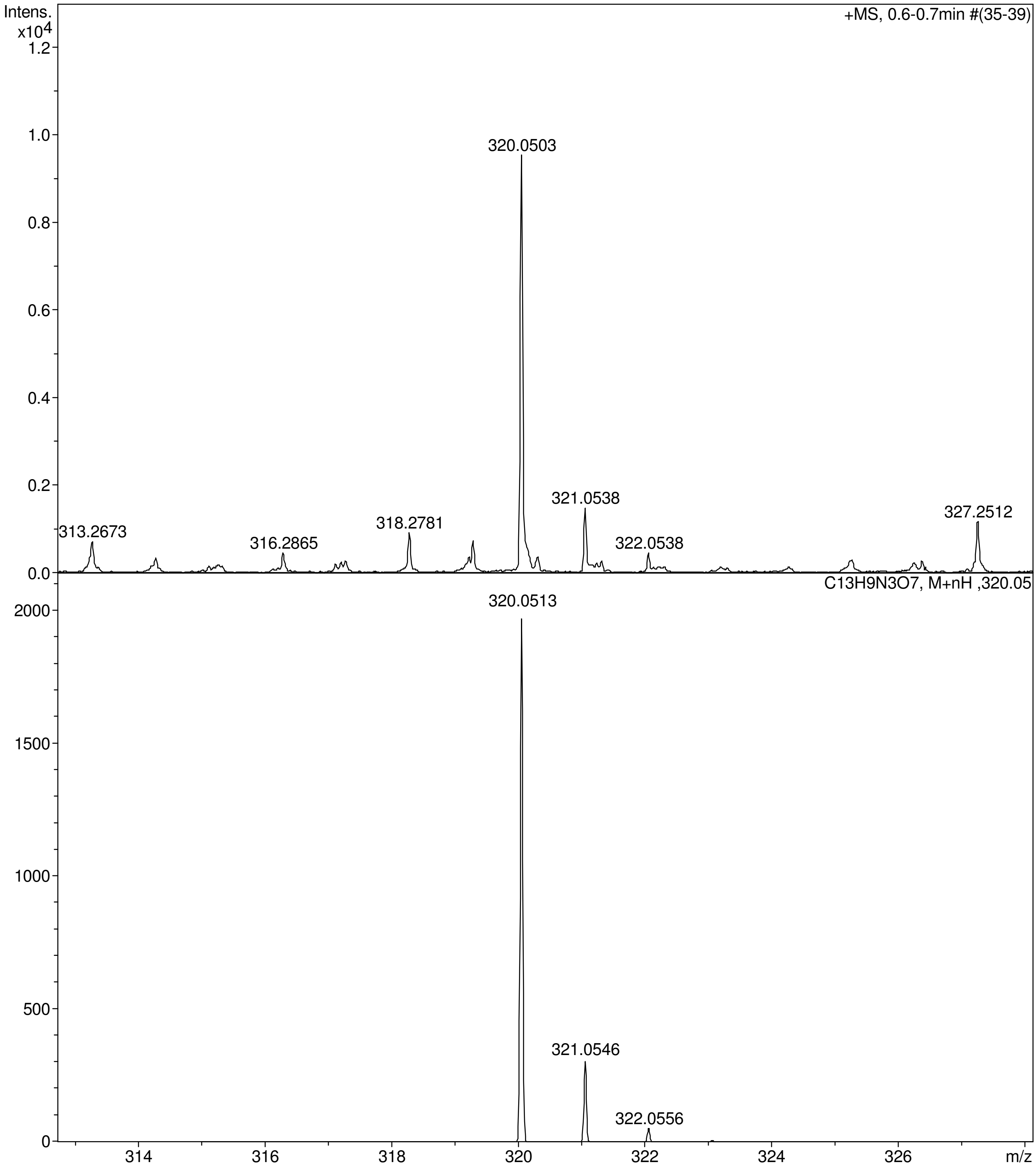

© Zelinsky Institute of Organic Chemistry, Moscow; Bruker AM300 SF=300.13 MHz {1H} SI=16K SW=6010 O1=2401 PW=9.0 AQ=1.352 RD=0.00 NS=1 SR=4.03 TE=301K 17 November 2020 Opr: Daeva E.D.; Solv: CDCl3;

The Best Applied NMR! On−Line: "http://nmr.ioc.ac.ru:8080"

/LPIK AF−391

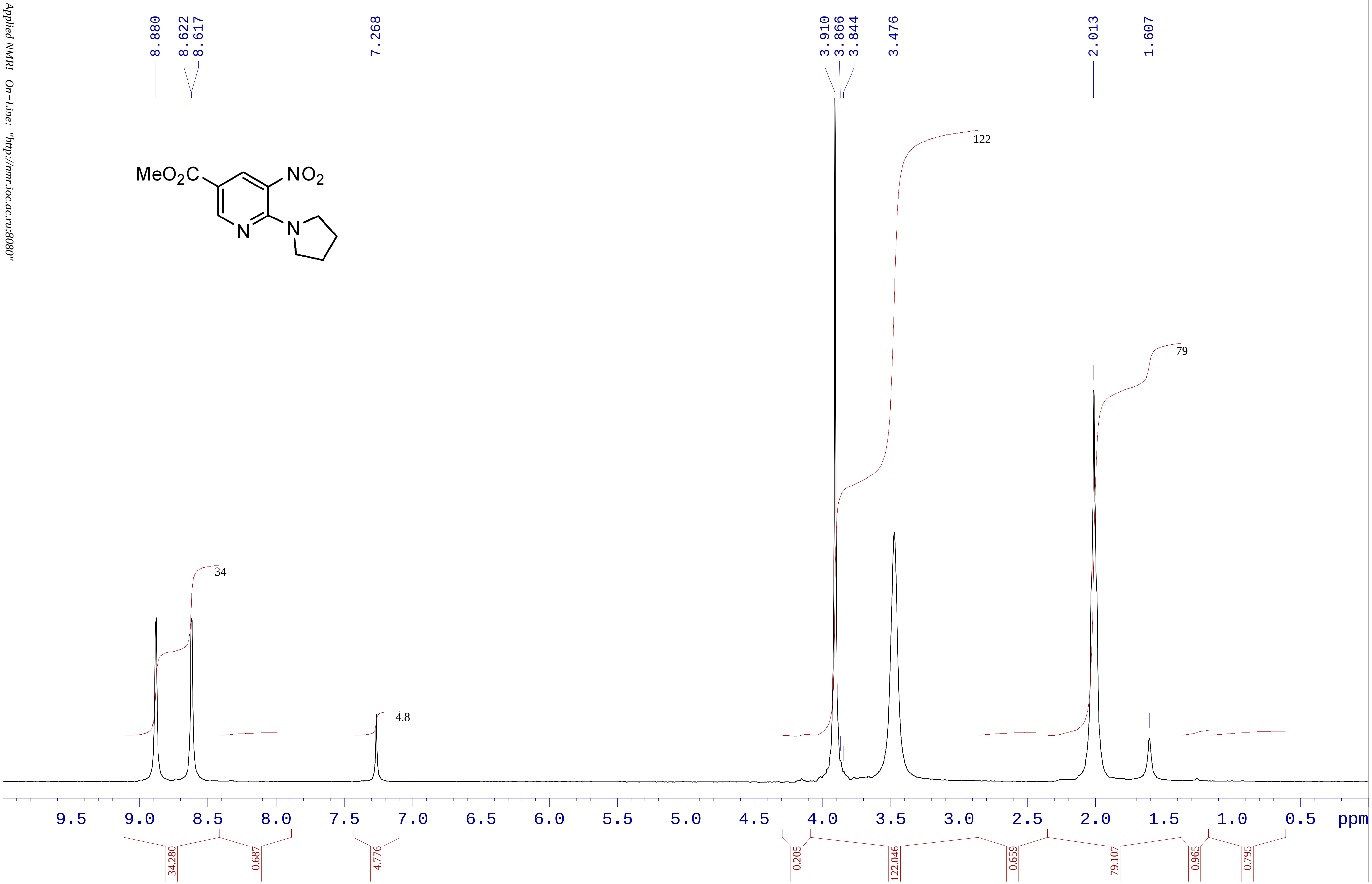

© Zelinsky Institute of Organic Chemistry, Moscow; Bruker AV300 SF=75.47 MHz {13C} SI=64K SW=18027 O1=7924 PW=9.0 AQ=0.905 RD=0.80 NS=64 SR=0.00 TE=300K 18 November 2020 Opr: NMR User; Solv: CDCl3;

The Best Applied NMR! On−Line: "http://nmr.ioc.ac.ru:8080"

/LPIK AF−391.13 Kokorekin−20259

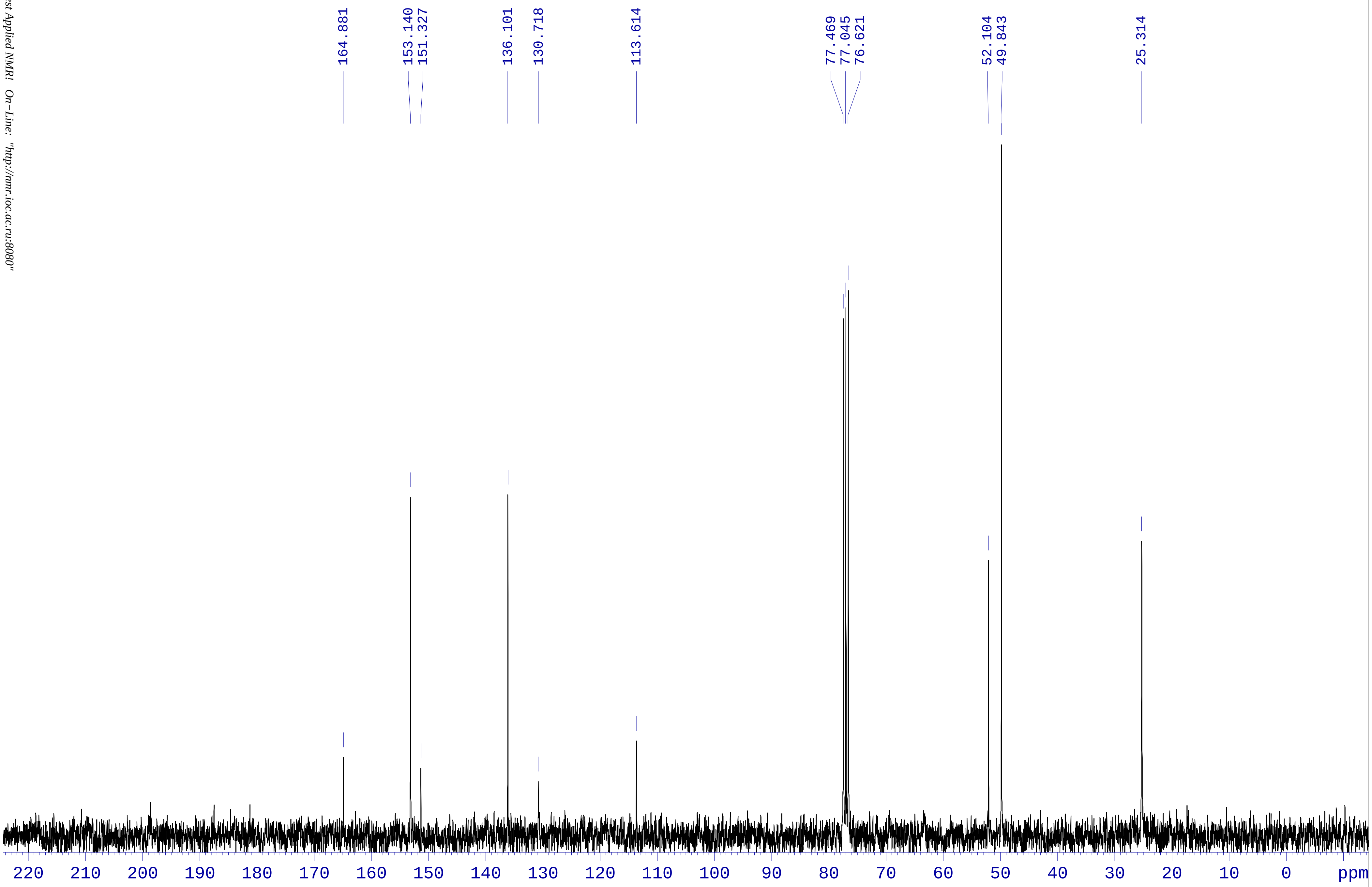

Display Report

Analysis Info

Analysis Name	D:\Data\Kolotyrkina\2021\Bastrakov\0210029.d	Acquisition Date	10.02.2021 20:56:14
Method	tune_50-1600.m	Operator	BDAL@DE
Sample Name	/LPIK AF-391	Instrument / Ser#	micrOTOF 10248
Comment	C11H13N3O4 mH 252.0979 calibrant added CH3CN		

Acquisition Parameter

Source Type	ESI	Ion Polarity	Positive	Set Nebulizer	1.0 Bar
Focus	Not active			Set Dry Heater	200 °C
Scan Begin	50 m/z	Set Capillary	4500 V	Set Dry Gas	4.0 l/min
Scan End	1600 m/z	Set End Plate Offset	-500 V	Set Divert Valve	Waste

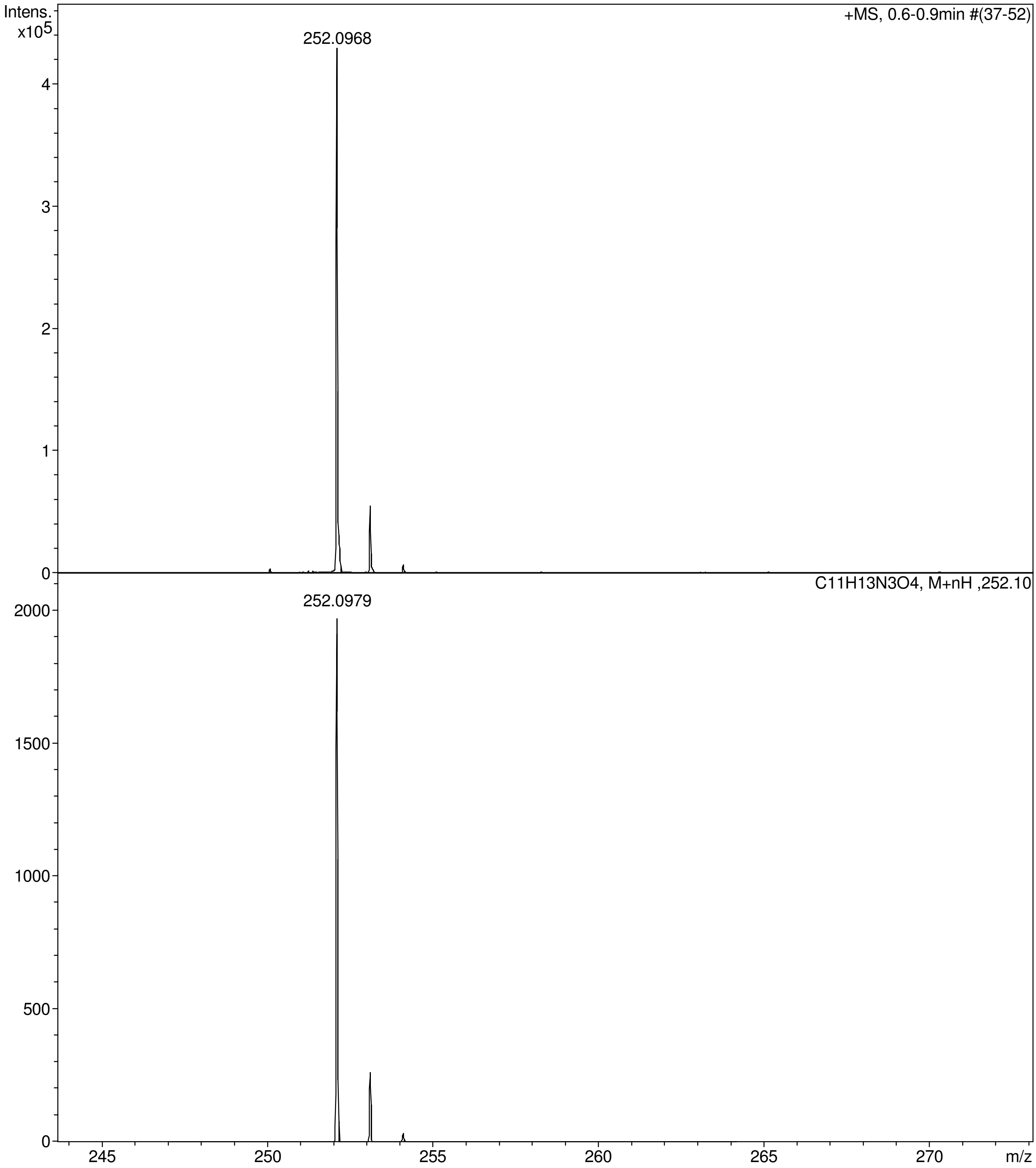

© Zelinsky Institute of Organic Chemistry, Moscow; Bruker AM300 SF=300.13 MHz {1H} SI=16K SW=6010 O1=2401 PW=9.0 AQ=1.352 RD=0.00 NS=1 SR=4.04 TE=299K 26 November 2020 Opr: Daeva E.D.; Solv: CDCl3;

The Best Applied NMR! On−Line: "http://nmr.ioc.ac.ru:8080"

/LPIK AF−399

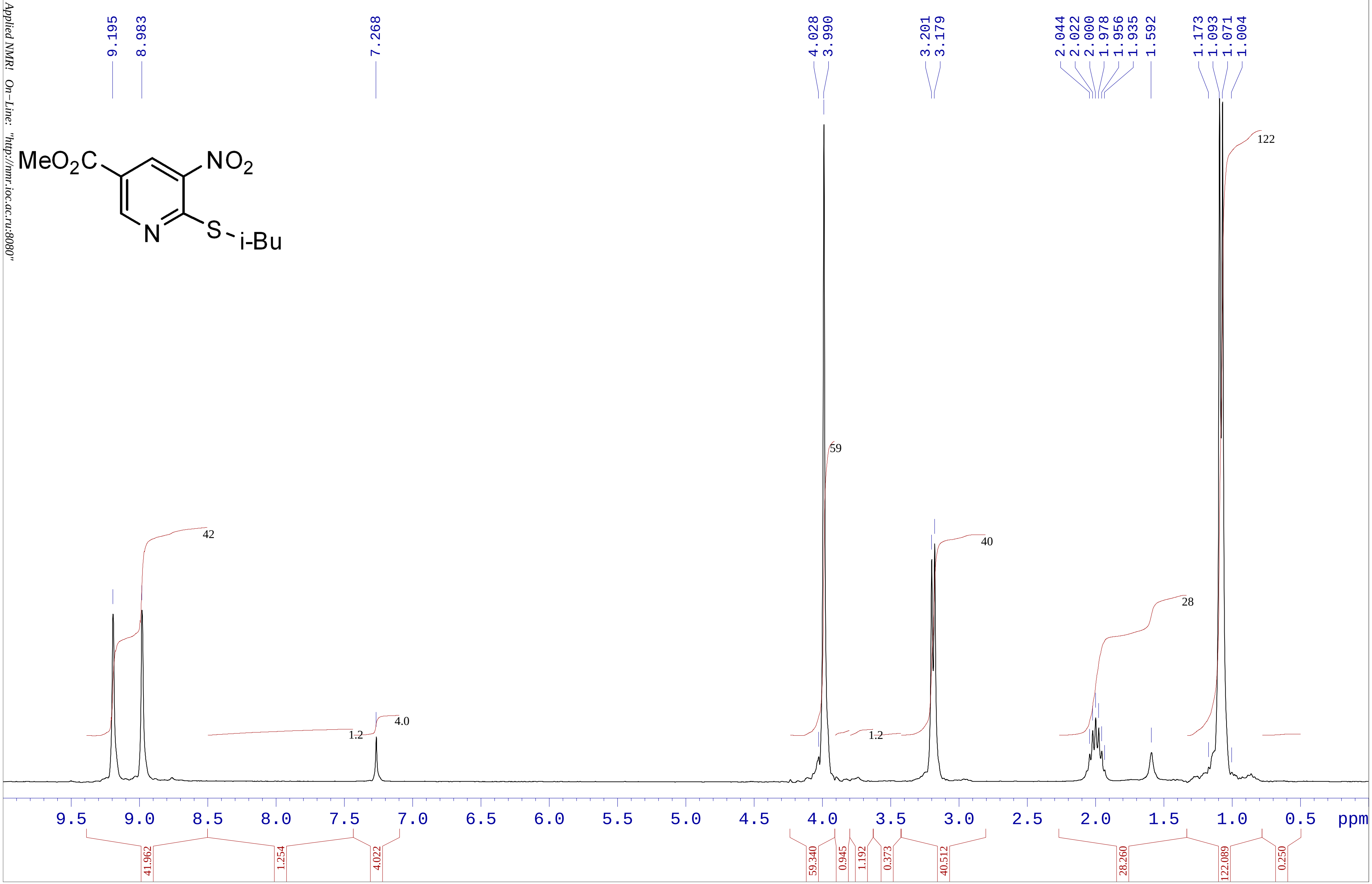

© Zelinsky Institute of Organic Chemistry, Moscow; Bruker AV300 SF=75.47 MHz {13C} SI=64K SW=18027 O1=7924 PW=9.0 AQ=0.905 RD=0.80 NS=143 SR=0.00 TE=300K 2 March 2021 Opr: NMR User; Solv: CDCl3;

The Best Applied NMR! On−Line: "http://nmr.ioc.ac.ru:8080"

/LPIK AF−399.13 Kokorekin−20259

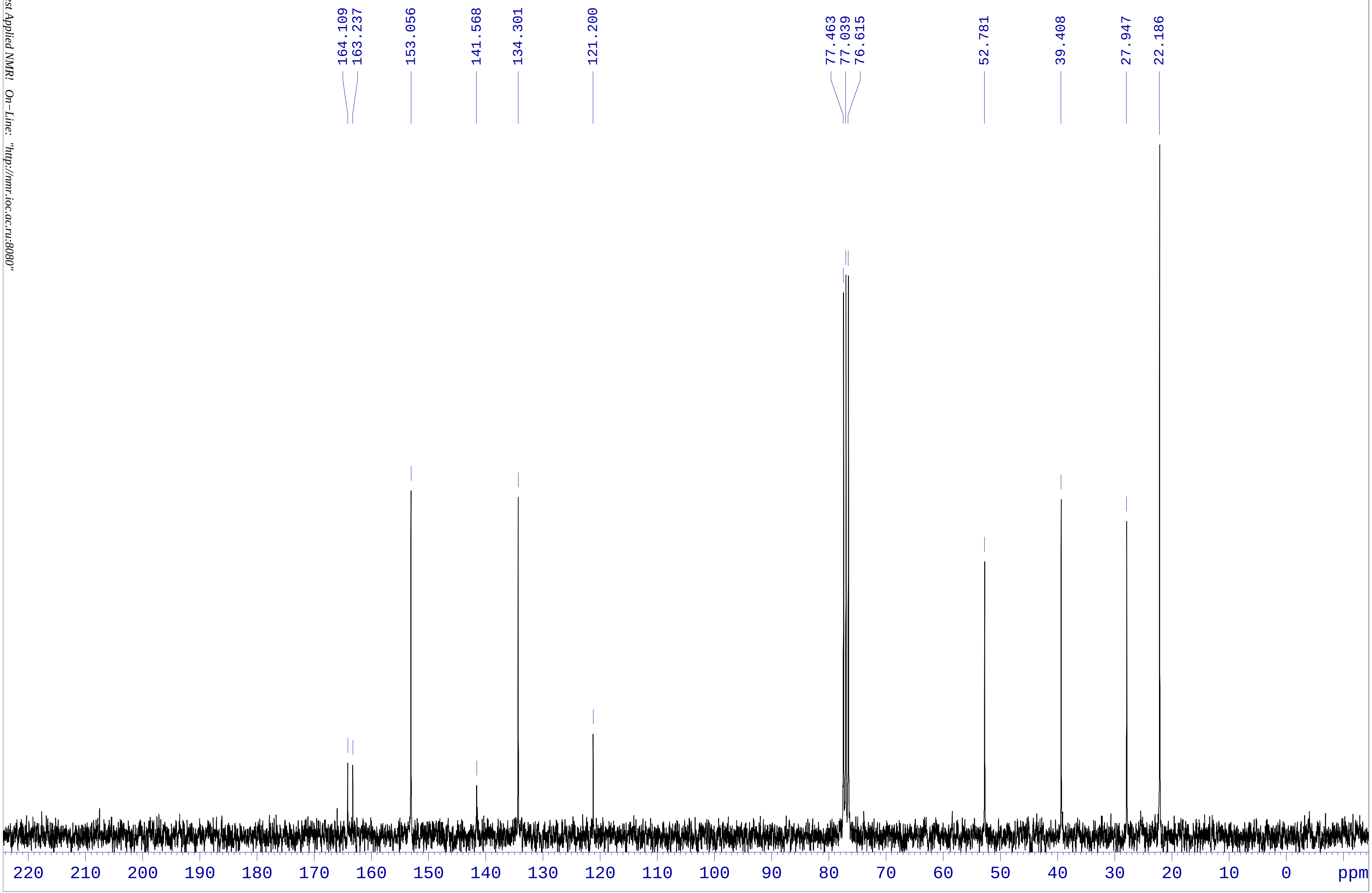

Display Report

Analysis Info

Analysis Name	D:\Data\Kolotyrkina\2021\Bastrakov\0211022.d	Acquisition Date	11.02.2021 12:57:08
Method	tune_50-1600.m	Operator	BDAL@DE
Sample Name	/LPIK AF-399	Instrument / Ser#	micrOTOF 10248
Comment	C11H14N2O4S mH 271.0747 calibrant added CH3OH		

Acquisition Parameter

Source Type	ESI	Ion Polarity	Positive	Set Nebulizer	1.0 Bar
Focus	Not active			Set Dry Heater	200 °C
Scan Begin	50 m/z	Set Capillary	4500 V	Set Dry Gas	4.0 l/min
Scan End	1600 m/z	Set End Plate Offset	-500 V	Set Divert Valve	Waste

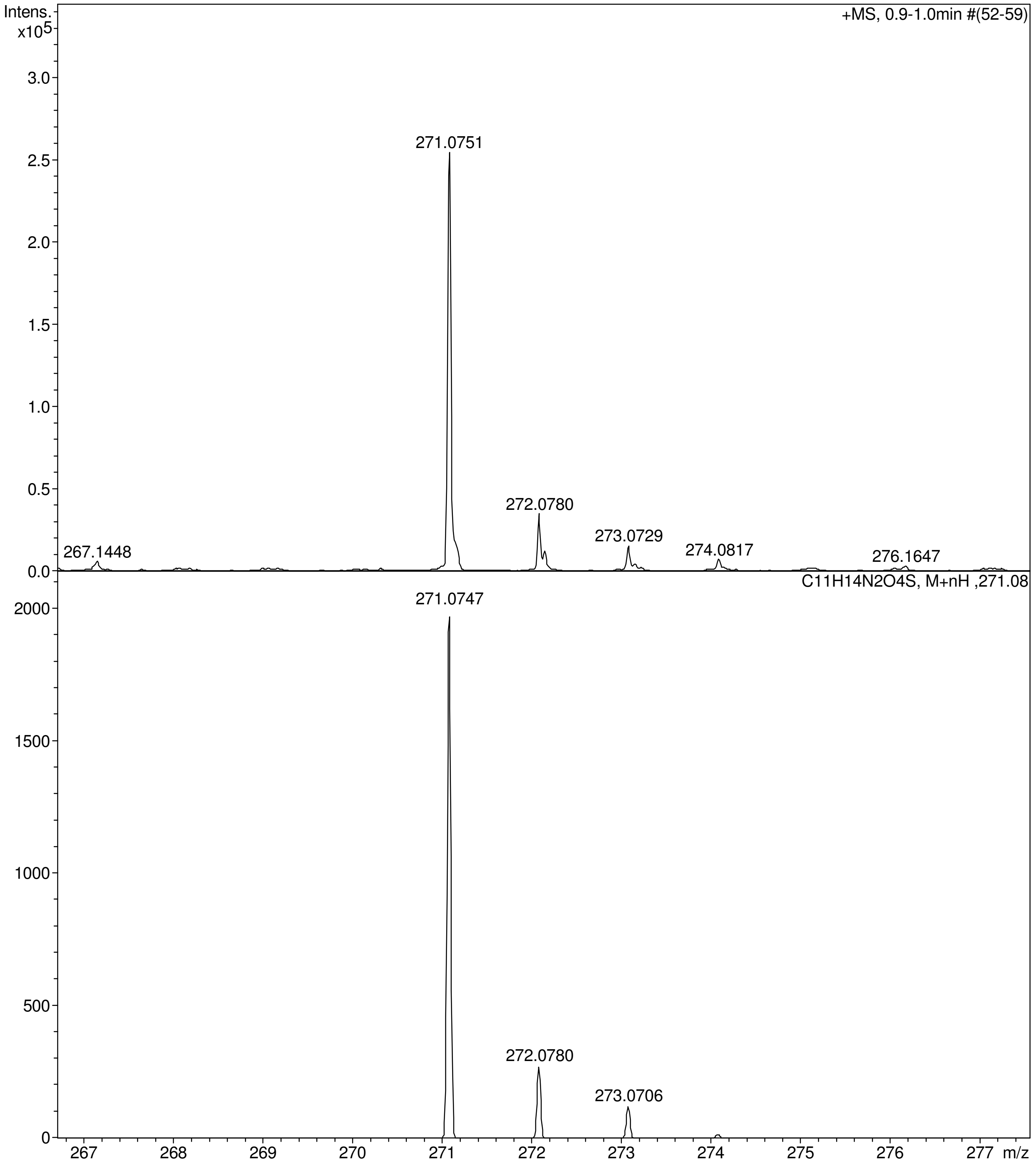

© Zelinsky Institute of Organic Chemistry, Moscow; Bruker AV300 SF=300.13 MHz {1H} SI=64K SW=6010 O1=2101 PW=15.0 AQ=2.715 RD=0.10 NS=1 SR=0.00 TE=300K 10 March 2021 Opr: NMR User; Solv: CDCl3;

The Best Applied NMR! On-Line: "http://nmr.ioc.ac.ru:8080"

/LPIK AF−406 Kokorekin−20259

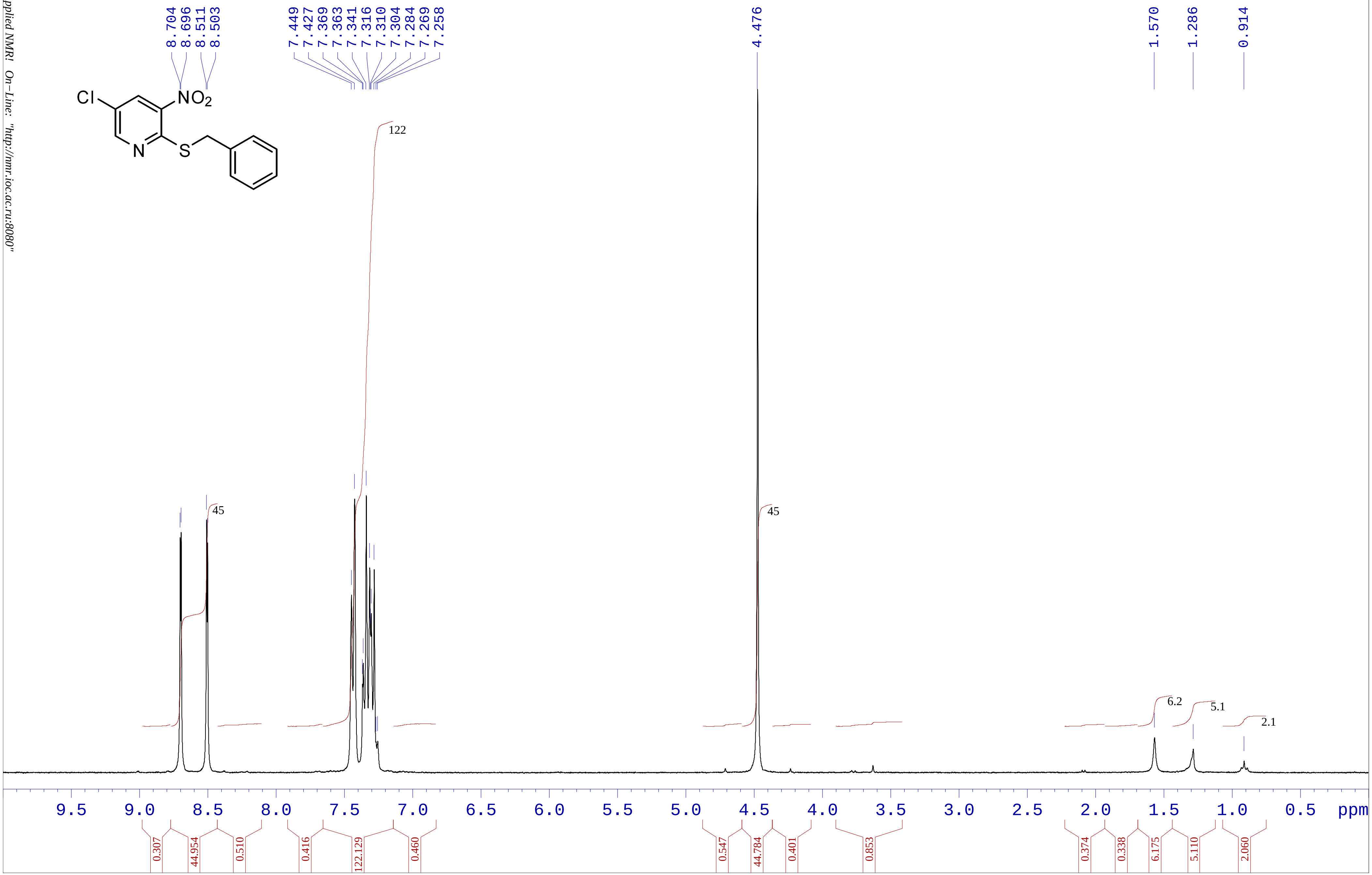

© Zelinsky Institute of Organic Chemistry, Moscow; Bruker AM300 SF=75.49 MHz {13C} SI=32K SW=17855 O1=7549 PW=10.0 AQ=1.143 RD=0.80 NS=200 SR=0.00 TE=298K 19 March 2021 Opr: NMR User; Solv: CDCl3;

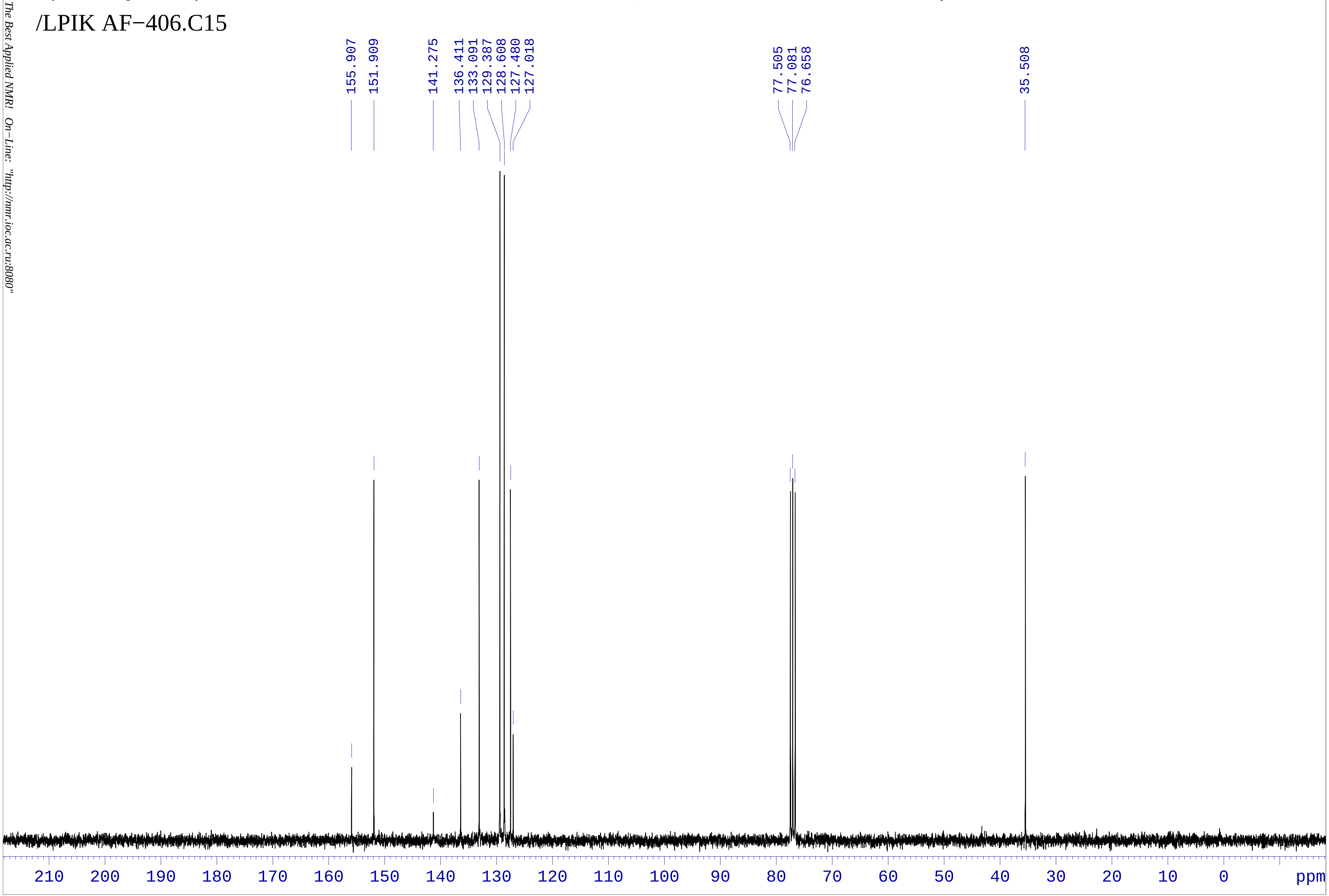

Display Report

Analysis Info

Analysis Name	D:\Data\Kolotyrkina\2021\Bastrakov\0428019.d	Acquisition Date	28.04.2021 12:10:25
Method	tune_50-1600.m	Operator	BDAL@DE
Sample Name	/LPIK AF-406	Instrument / Ser#	micrOTOF 10248
Comment	C12H9ClN2O2S mH 281.0146 clb added CH3OH		

Acquisition Parameter

Source Type	ESI	Ion Polarity	Positive	Set Nebulizer	1.0 Bar
Focus	Not active			Set Dry Heater	200 °C
Scan Begin	50 m/z	Set Capillary	4500 V	Set Dry Gas	4.0 l/min
Scan End	1600 m/z	Set End Plate Offset	-500 V	Set Divert Valve	Waste

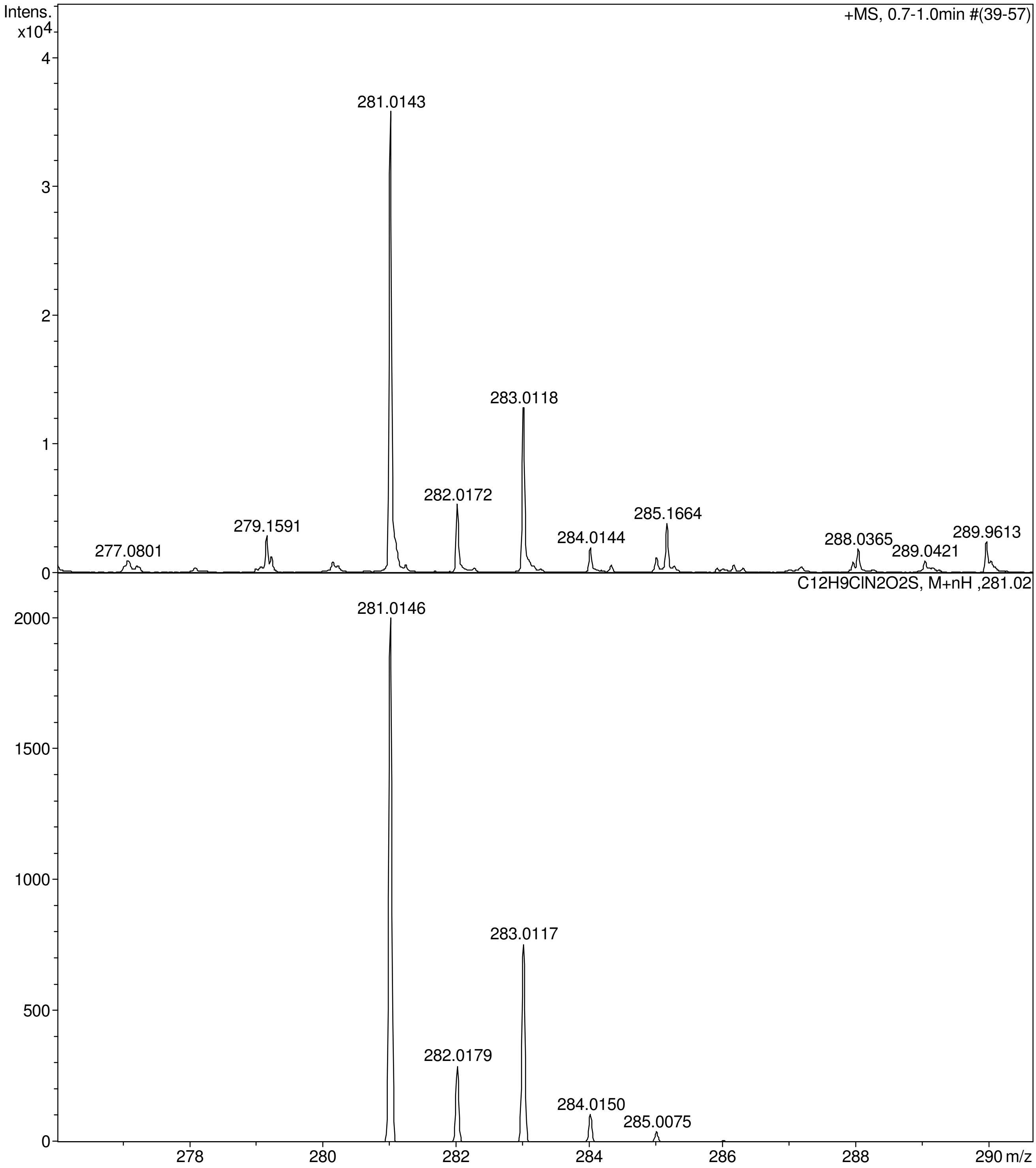

© Zelinsky Institute of Organic Chemistry, Moscow; Bruker AM300 SF=300.13 MHz {1H} SI=32K SW=6010 O1=2401 PW=9.0 AQ=1.352 RD=0.00 NS=1 SR=0.00 TE=300K 16 March 2021 Opr: Daeva E.D.; Solv: CDCl3;

The Best Applied NMR! On−Line: "http://nmr.ioc.ac.ru:8080"

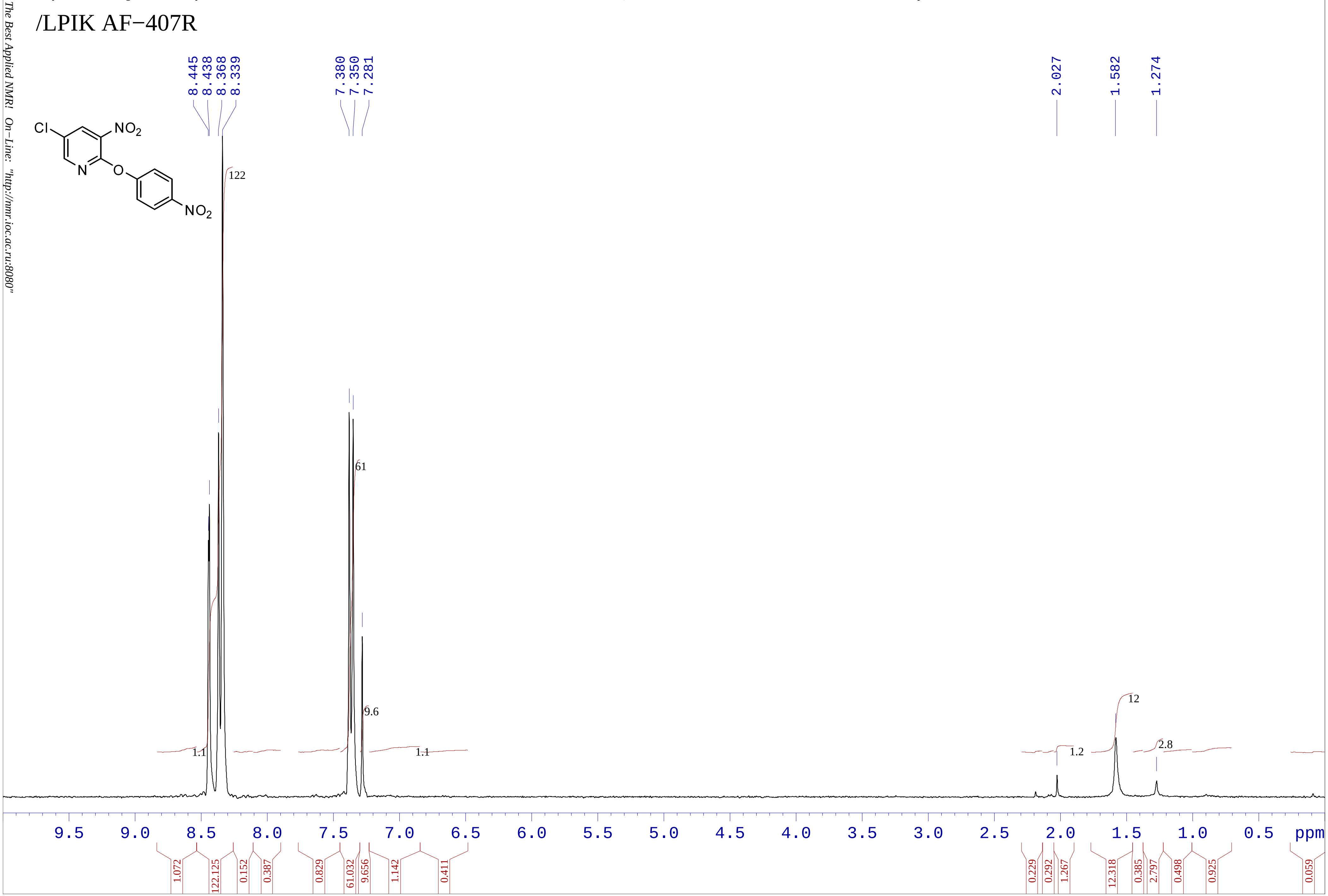

© Zelinsky Institute of Organic Chemistry, Moscow; Bruker AM300 SF=75.49 MHz {13C} SI=32K SW=17855 O1=7549 PW=10.0 AQ=1.143 RD=0.80 NS=200 SR=0.00 TE=298K 19 March 2021 Opr: NMR User; Solv: CDCl3;

The Best Applied NMR! On−Line: "http://nmr.ioc.ac.ru:8080"

/LPIK AF−407.C13

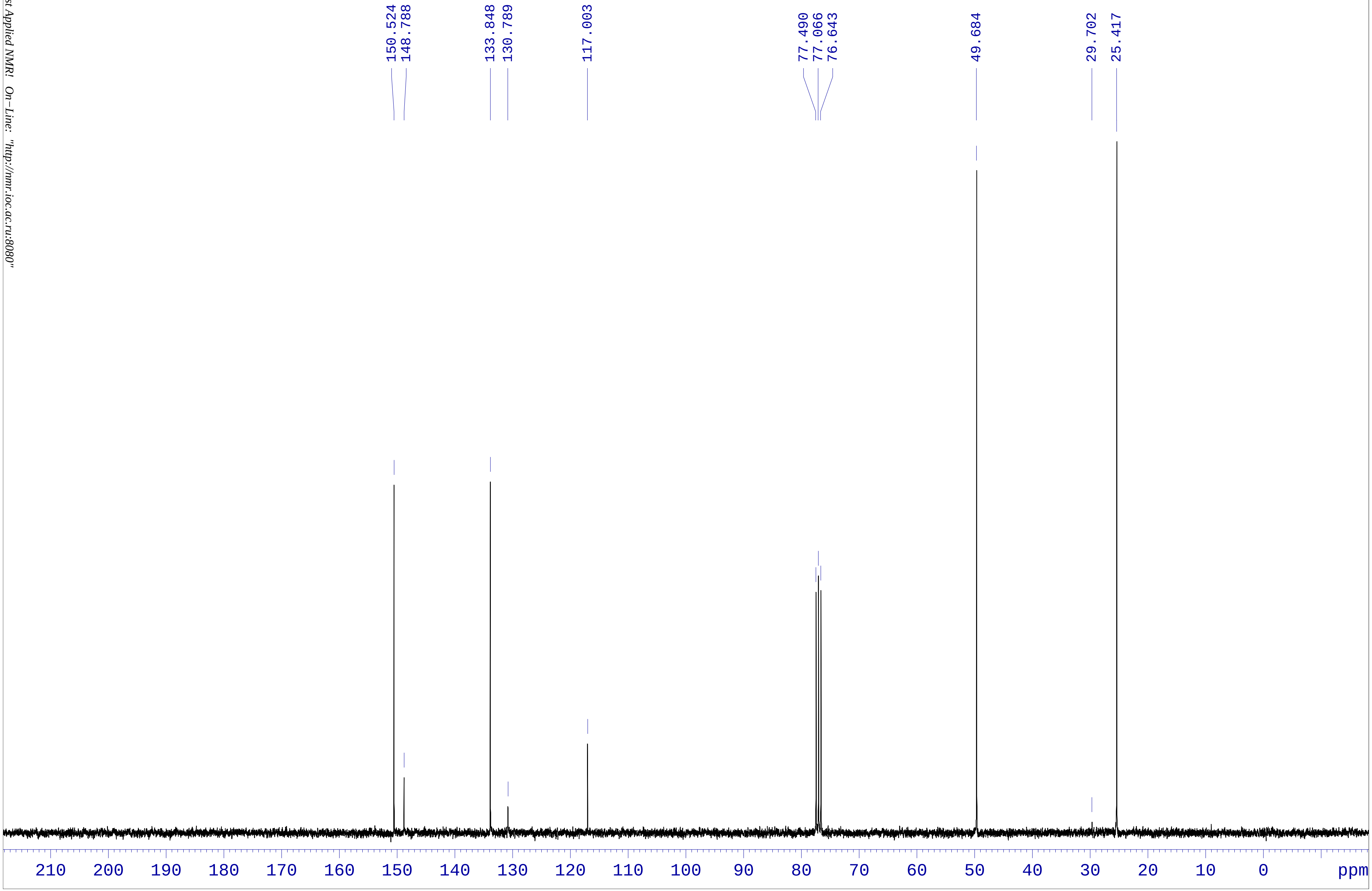

Display Report

Analysis Info

Analysis Name	D:\Data\Kolotyrkina\2021\Bastrakov\0428020.d	Acquisition Date	28.04.2021 12:16:29
Method	tune_50-1600.m	Operator	BDAL@DE
Sample Name	/LPIK AF-407	Instrument / Ser#	micrOTOF 10248
Comment	C11H6ClN3O5 mH 296.0068 clb added CH3OH		

Acquisition Parameter

Source Type	ESI	Ion Polarity	Positive	Set Nebulizer	1.0 Bar
Focus	Not active			Set Dry Heater	200 °C
Scan Begin	50 m/z	Set Capillary	4500 V	Set Dry Gas	4.0 l/min
Scan End	1600 m/z	Set End Plate Offset	-500 V	Set Divert Valve	Waste

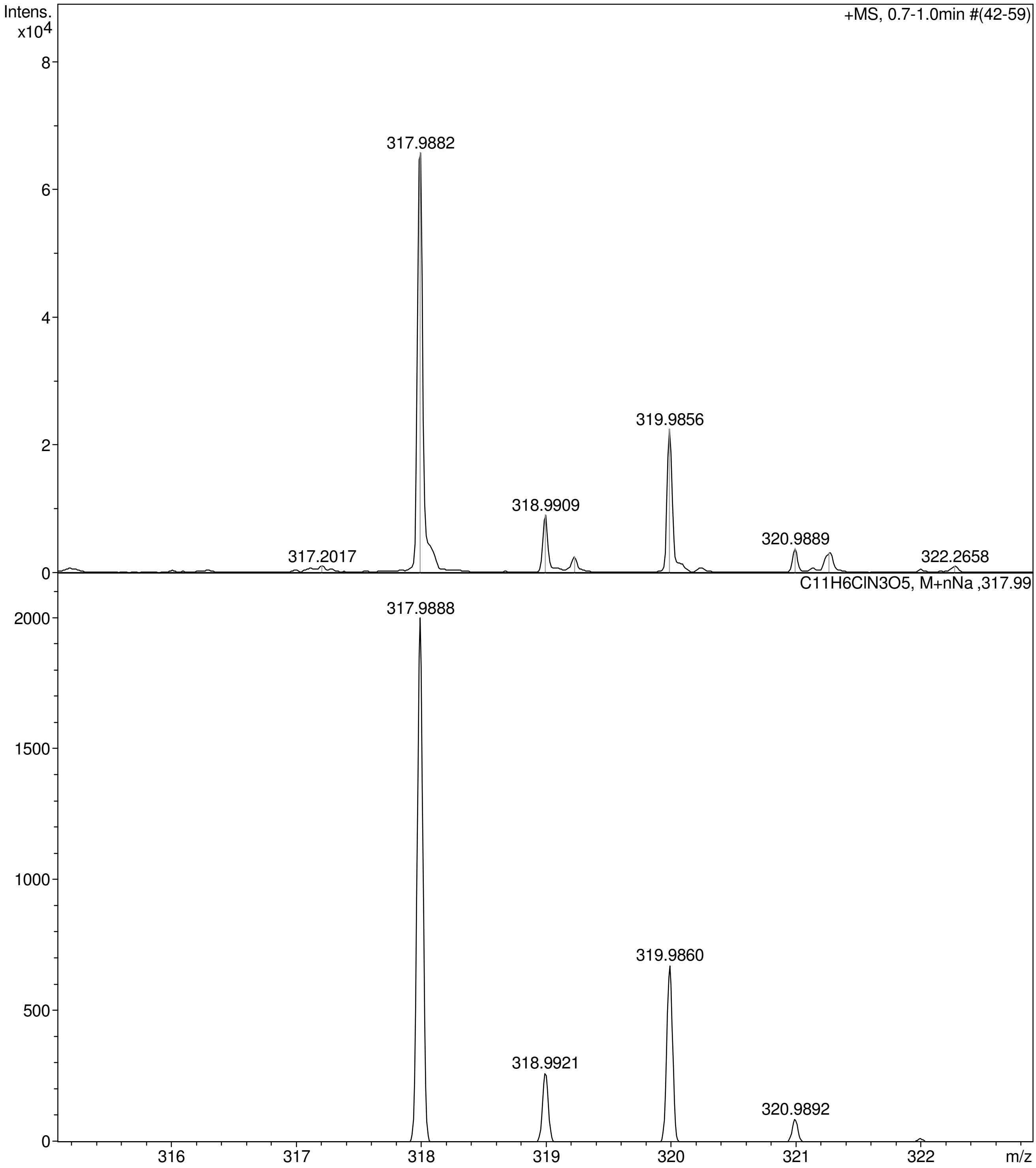

© Zelinsky Institute of Organic Chemistry, Moscow; Bruker AV300 SF=300.13 MHz {1H} SI=64K SW=6010 O1=2101 PW=15.0 AQ=2.715 RD=0.10 NS=1 SR=0.00 TE=300K 10 March 2021 Opr: NMR User; Solv: CDCl3;

The Best Applied NMR! On−Line: "http://nmr.ioc.ac.ru:8080"

/LPIK AF−408 Kokorekin−20259

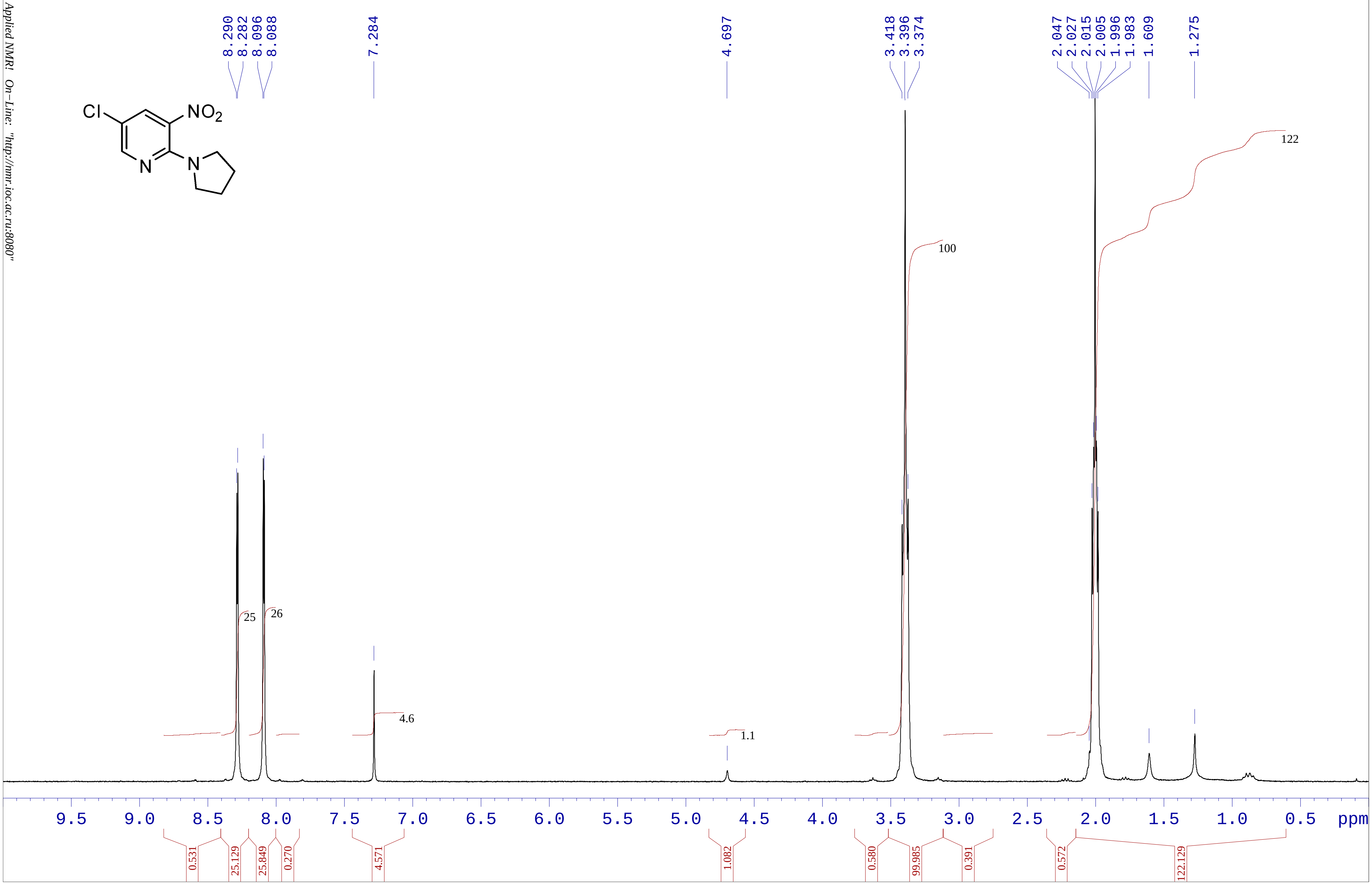

© Zelinsky Institute of Organic Chemistry, Moscow; Bruker AM300 SF=75.49 MHz {13C} SI=32K SW=17855 O1=7549 PW=10.0 AQ=1.143 RD=0.80 NS=200 SR=0.00 TE=298K 19 March 2021 Opr: NMR User; Solv: CDCl3;

The Best Applied NMR! On−Line: "http://nmr.ioc.ac.ru:8080"

/LPIK AF−408.C13

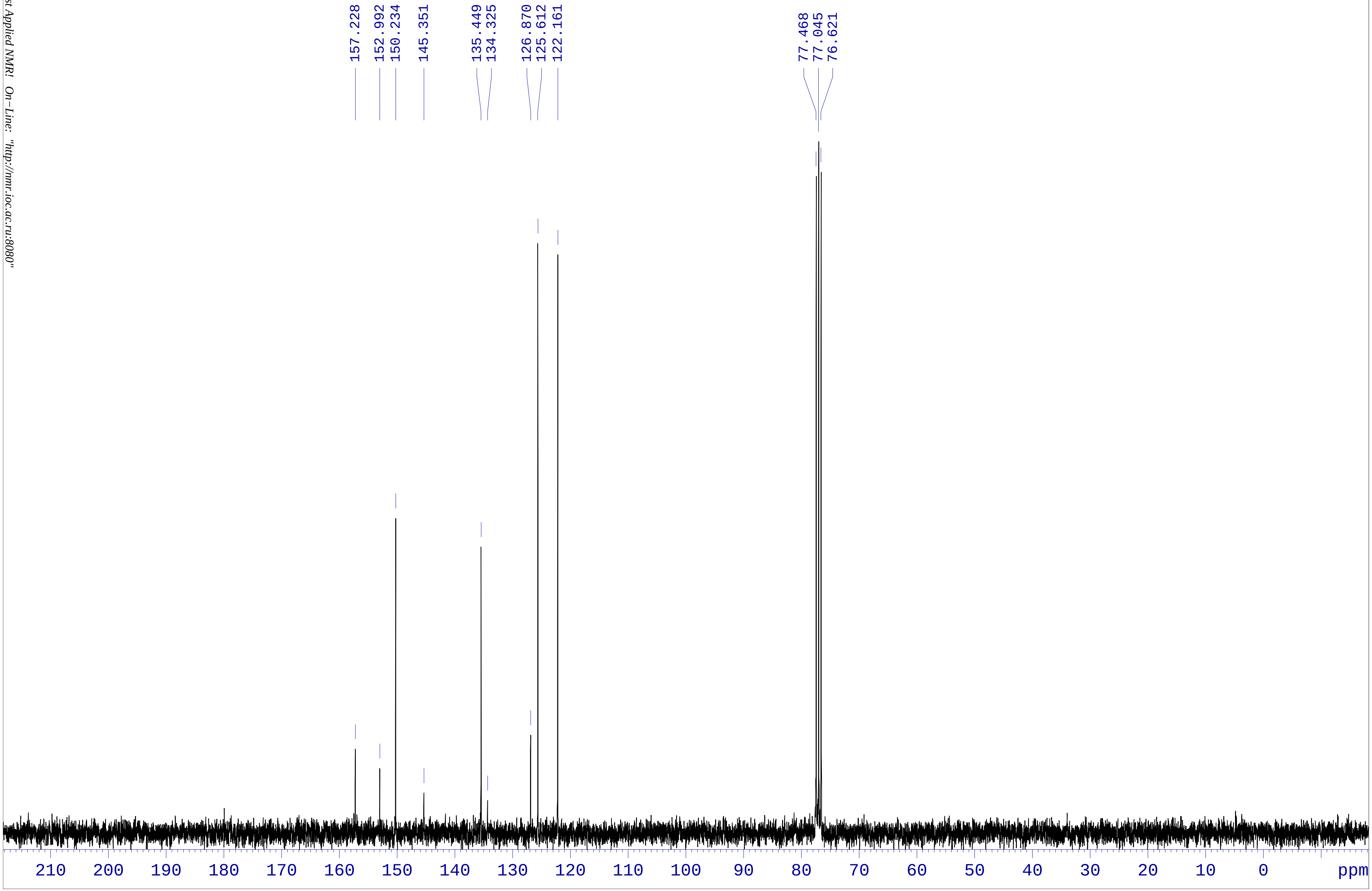

Display Report

Analysis Info

Analysis Name	D:\Data\Kolotyrkina\2021\Bastrakov\0428021.d	Acquisition Date	28.04.2021 12:28:48
Method	tune_50-1600.m	Operator	BDAL@DE
Sample Name	/LPIK AF-408	Instrument / Ser#	micrOTOF 10248
Comment	C9H10ClN3O2 mH 228.0534 clb added CH3OH		

Acquisition Parameter

Source Type	ESI	Ion Polarity	Positive	Set Nebulizer	1.0 Bar
Focus	Not active			Set Dry Heater	200 °C
Scan Begin	50 m/z	Set Capillary	4500 V	Set Dry Gas	4.0 l/min
Scan End	1600 m/z	Set End Plate Offset	-500 V	Set Divert Valve	Waste

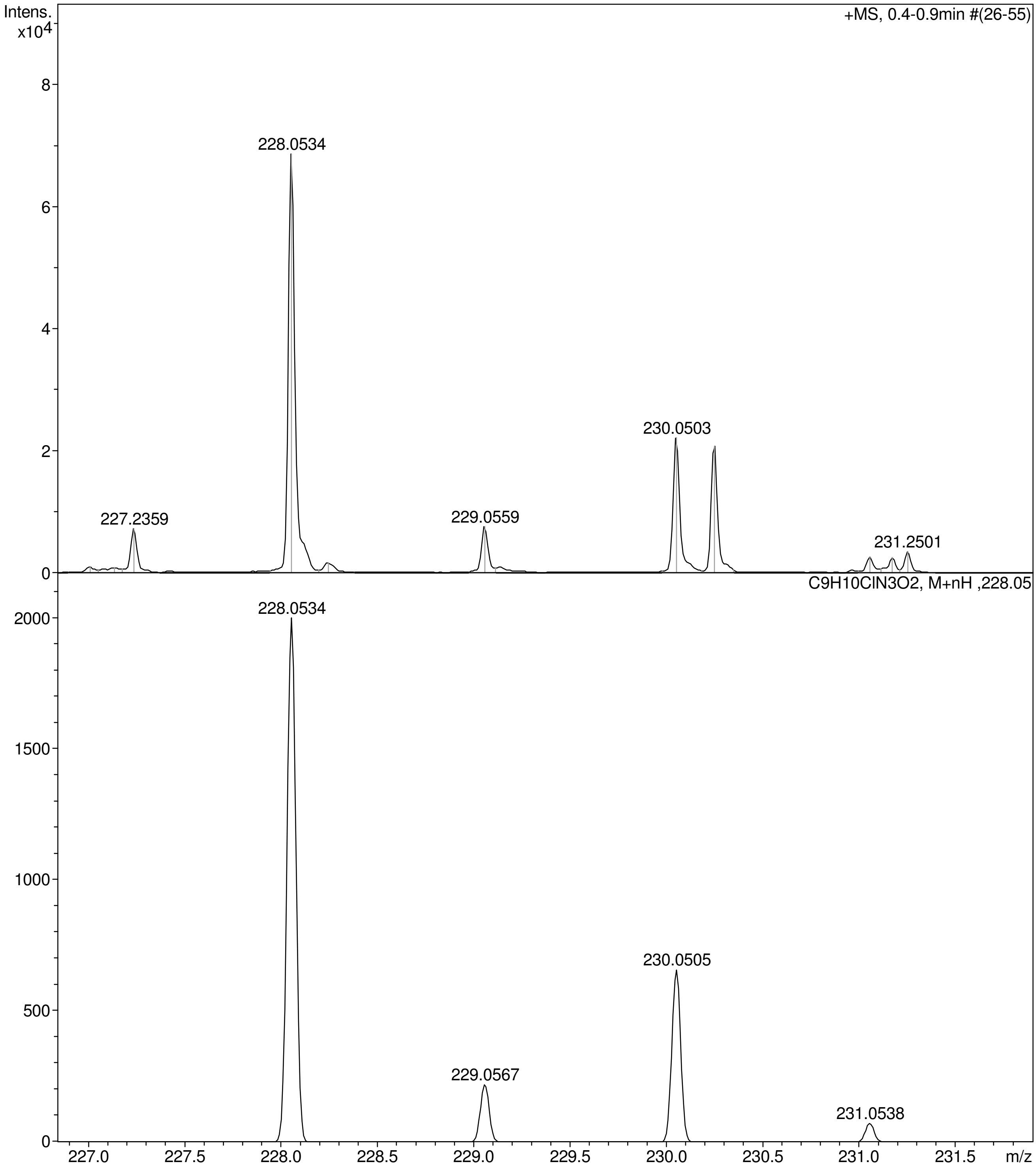

© Zelinsky Institute of Organic Chemistry, Moscow; Bruker AM300 SF=300.13 MHz {1H} SI=32K SW=6010 O1=2401 PW=9.0 AQ=1.352 RD=0.00 NS=1 SR=0.00 TE=299K 22 March 2021 Opr: Daeva E.D.; Solv: CDCl3;

The Best Applied NMR! On−Line: "http://nmr.ioc.ac.ru:8080"

/LPIK AF−416

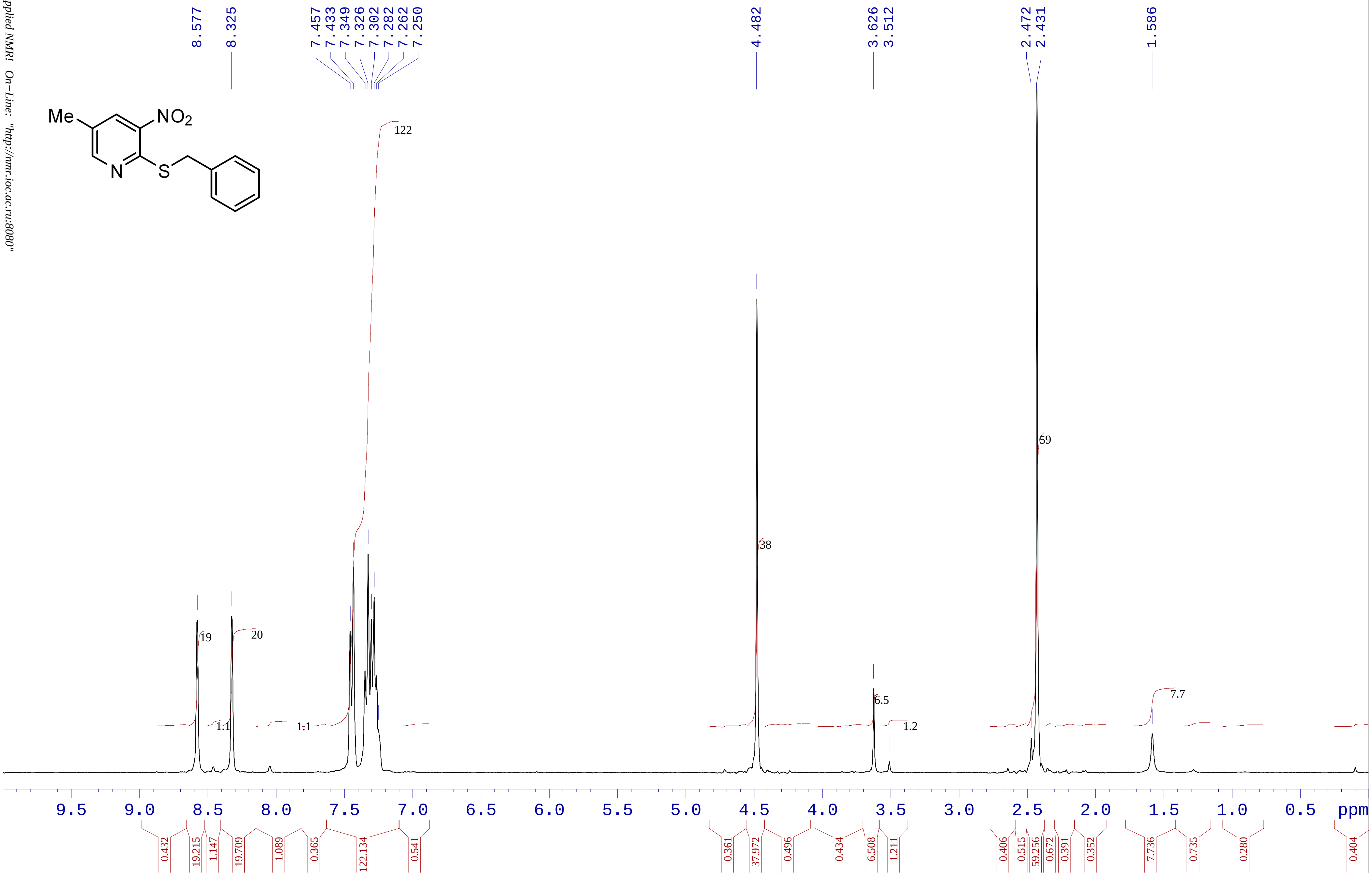

© Zelinsky Institute of Organic Chemistry, Moscow; Bruker AV300 SF=75.47 MHz {13C} SI=64K SW=18027 O1=7924 PW=9.0 AQ=0.905 RD=0.80 NS=103 SR=0.00 TE=300K 24 March 2021 Opr: NMR User; Solv: CDCl3;

The Best Applied NMR! On−Line: "http://nmr.ioc.ac.ru:8080"

/LPIK AF−416.13 Kokorekin−20259

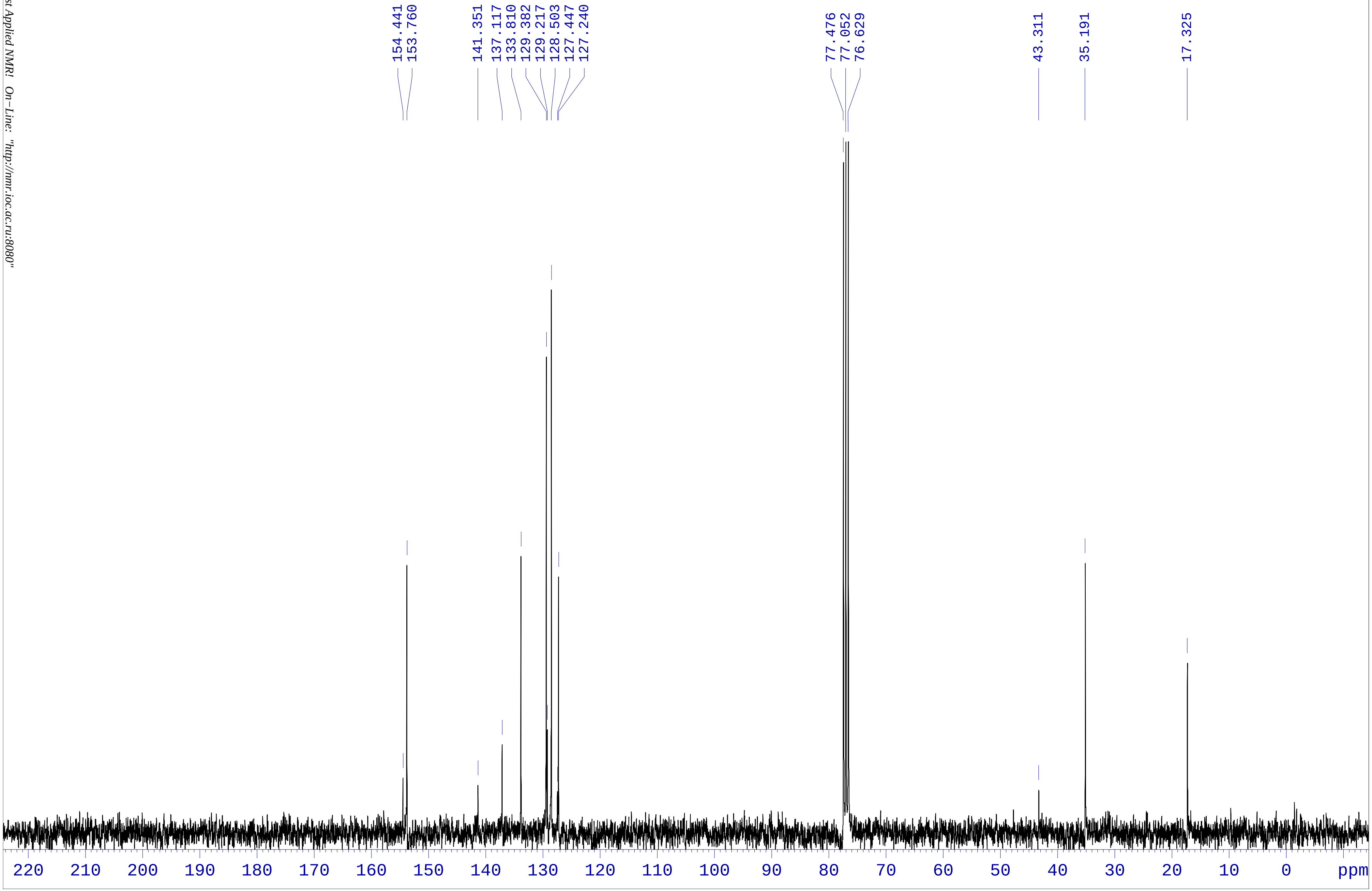

Display Report

Analysis Info

Analysis Name	D:\Data\Kolotyrkina\2021\Bastrakov\0428022.d	Acquisition Date	28.04.2021 12:33:48
Method	tune_50-1600.m	Operator	BDAL@DE
Sample Name	/LPIK AF-416	Instrument / Ser#	micrOTOF 10248
Comment	C13H12N2O2S mH 261.0692 clb added CH3OH		

Acquisition Parameter

Source Type	ESI	Ion Polarity	Positive	Set Nebulizer	1.0 Bar
Focus	Not active			Set Dry Heater	200 °C
Scan Begin	50 m/z	Set Capillary	4500 V	Set Dry Gas	4.0 l/min
Scan End	1600 m/z	Set End Plate Offset	-500 V	Set Divert Valve	Waste

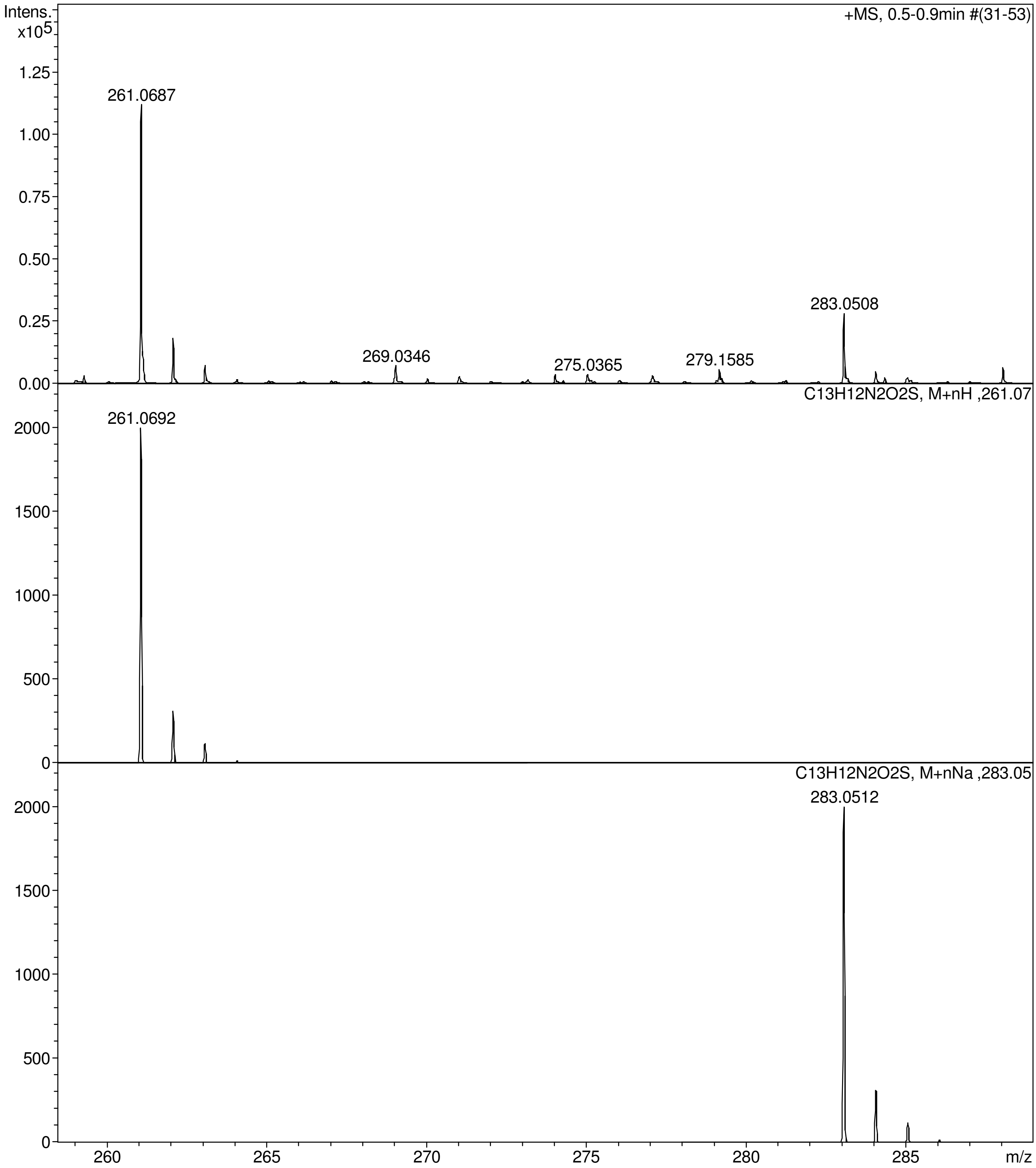

© Zelinsky Institute of Organic Chemistry, Moscow; Bruker AM300 SF=300.13 MHz {1H} SI=16K SW=6010 O1=2401 PW=9.0 AQ=1.351 RD=1.00 NS=1 SR=24.66 TE=298K 31 March 2021 Opr: Shulishov Eu.V.; Solv: CDCl3;

The Best Applied NMR! On-Line: "http://nmr.ioc.ac.ru:8080"

/LPIK AF_421

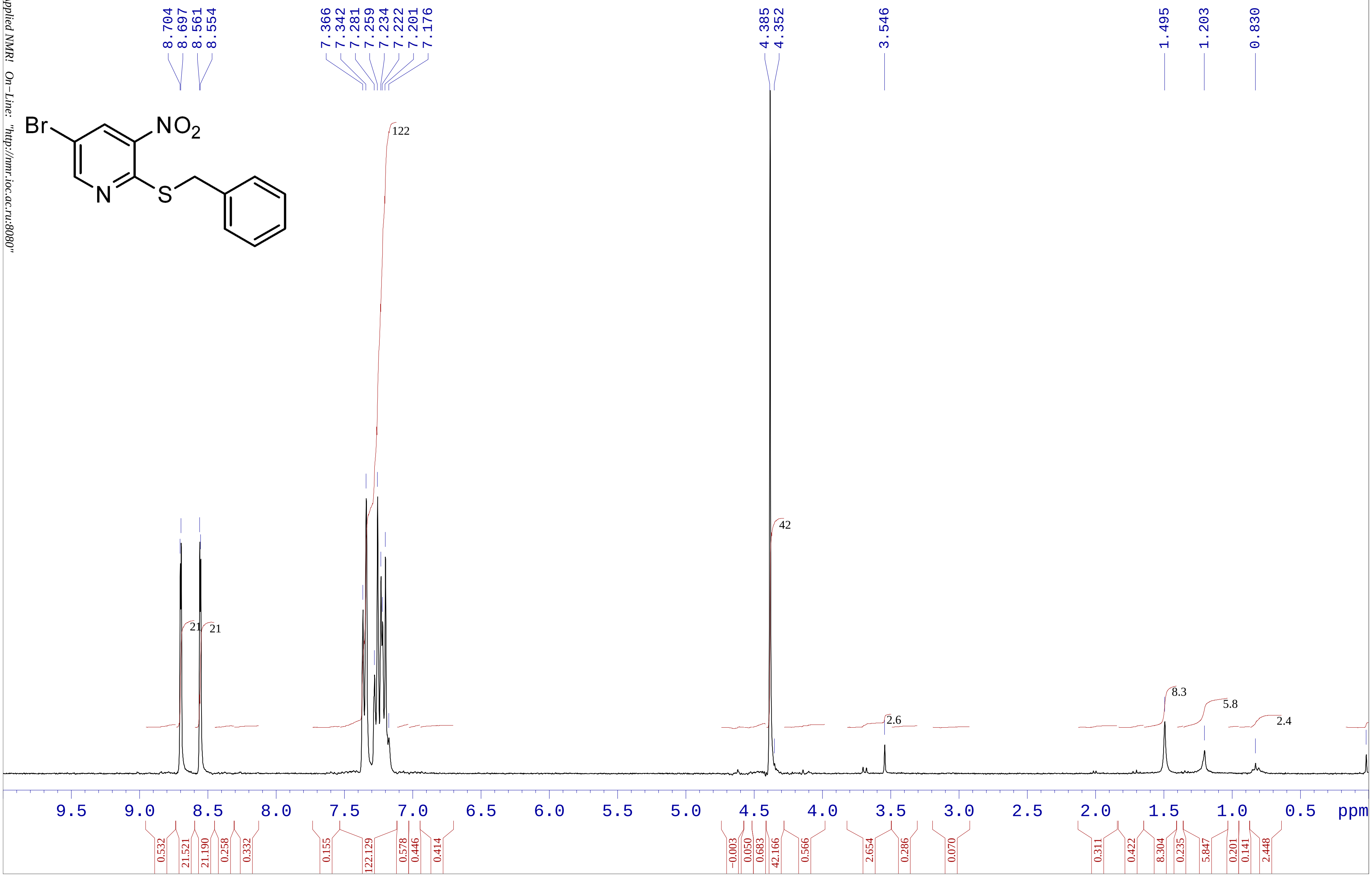

© Zelinsky Institute of Organic Chemistry, Moscow; Bruker AV300 SF=75.47 MHz {13C} SI=64K SW=18027 O1=7924 PW=9.0 AQ=0.905 RD=0.80 NS=146 SR=0.00 TE=300K 5 April 2021 Opr: NMR User; Solv: CDCl3;

The Best Applied NMR! On−Line: "http://nmr.ioc.ac.ru:8080"

/LPIK AF−421.13 Kokorekin−20259

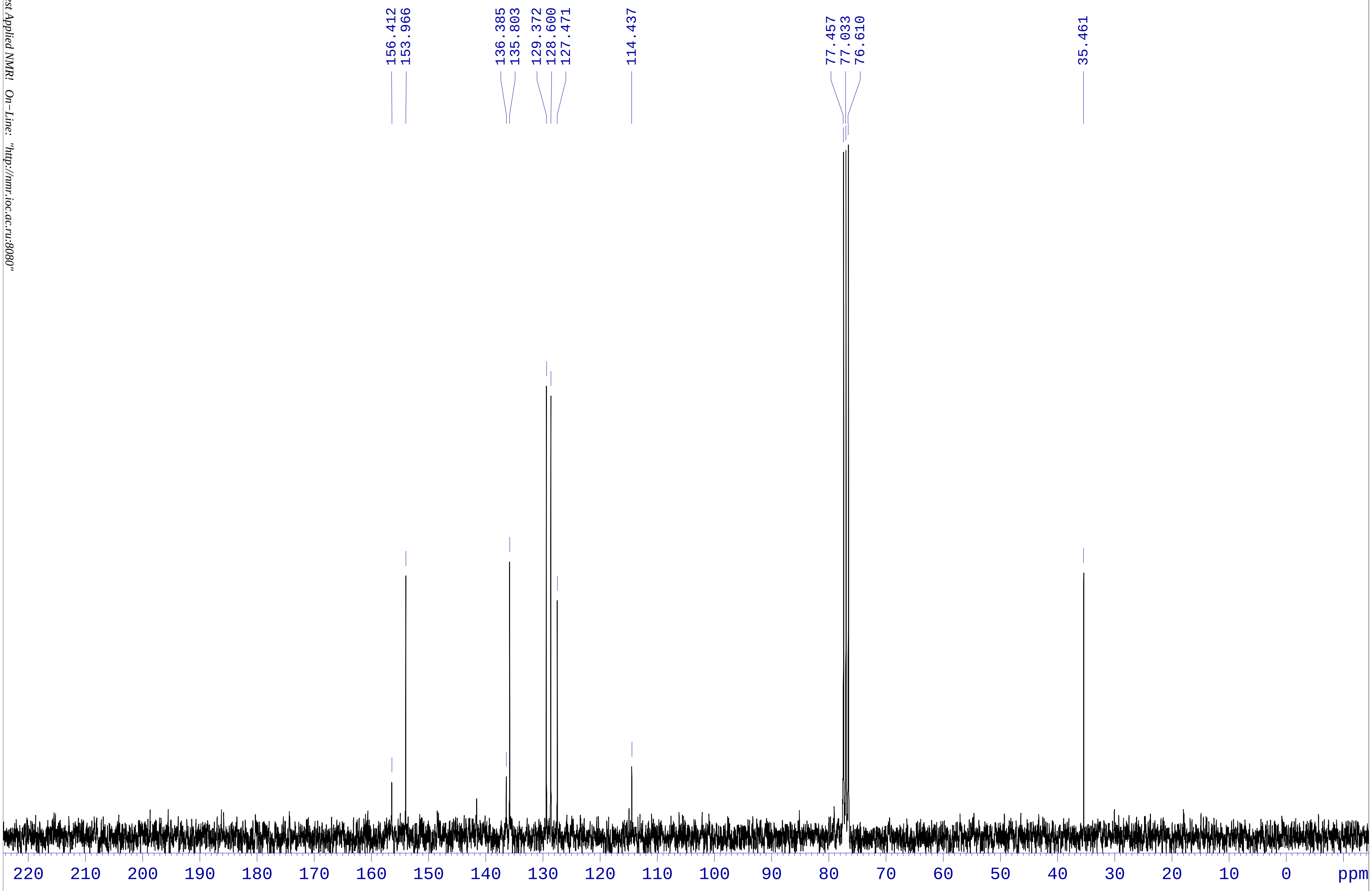

Display Report

Analysis Info

Analysis Name	D:\Data\Kolotyrkina\2021\Bastrakov\0428023.d	Acquisition Date	28.04.2021 12:38:09
Method	tune_50-1600.m	Operator	BDAL@DE
Sample Name	/LPIK AF-421	Instrument / Ser#	micrOTOF 10248
Comment	C12H9BrN2O2S mH 324.9640 clb added CH3OH		

Acquisition Parameter

Source Type	ESI	Ion Polarity	Positive	Set Nebulizer	1.0 Bar
Focus	Not active			Set Dry Heater	200 °C
Scan Begin	50 m/z	Set Capillary	4500 V	Set Dry Gas	4.0 l/min
Scan End	1600 m/z	Set End Plate Offset	-500 V	Set Divert Valve	Waste

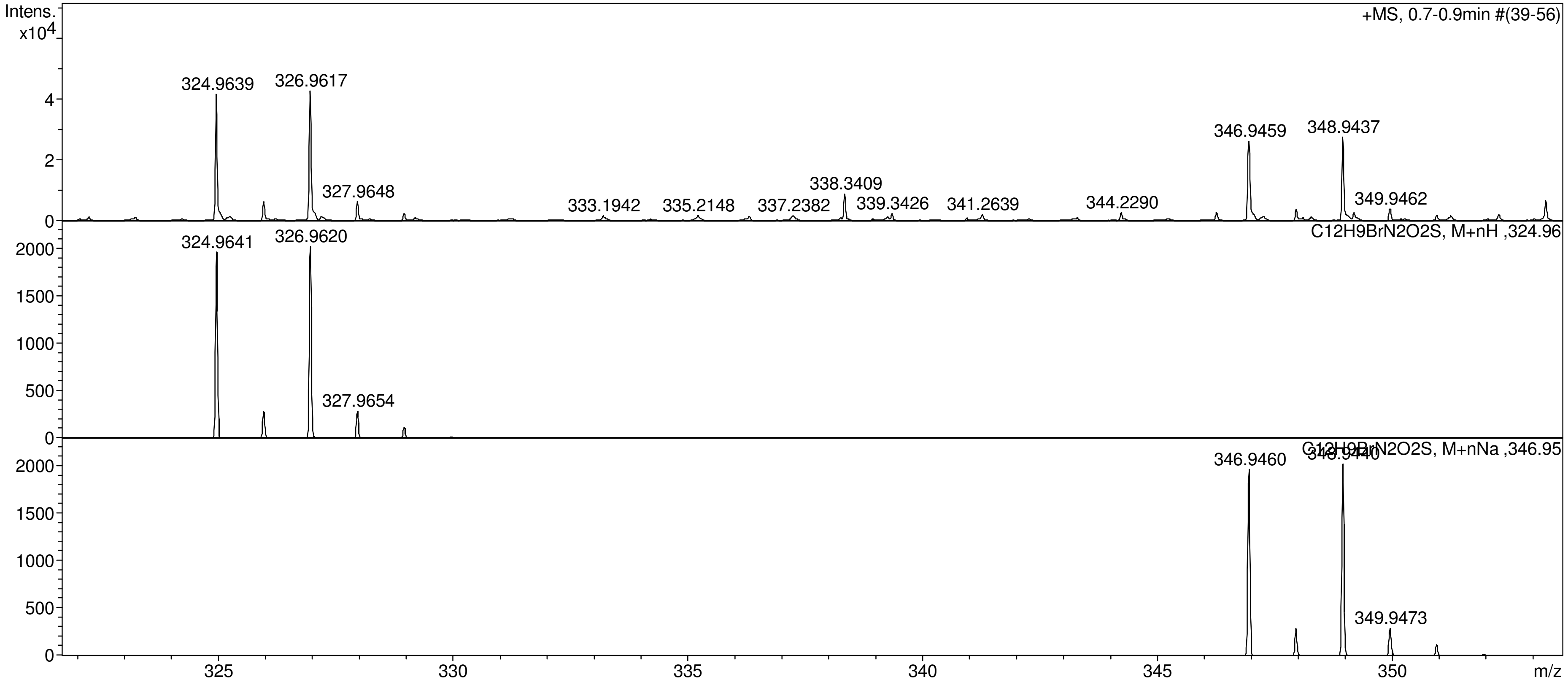

© Zelinsky Institute of Organic Chemistry, Moscow; Bruker AM300 SF=300.13 MHz {1H} SI=16K SW=6010 O1=2401 PW=9.0 AQ=1.351 RD=1.00 NS=1 SR=24.66 TE=298K 31 March 2021 Opr: Shulishov Eu.V.; Solv: CDCl3;

The Best Applied NMR! On-Line: "http://nmr.ioc.ac.ru:8080"

/LPIK AF_422

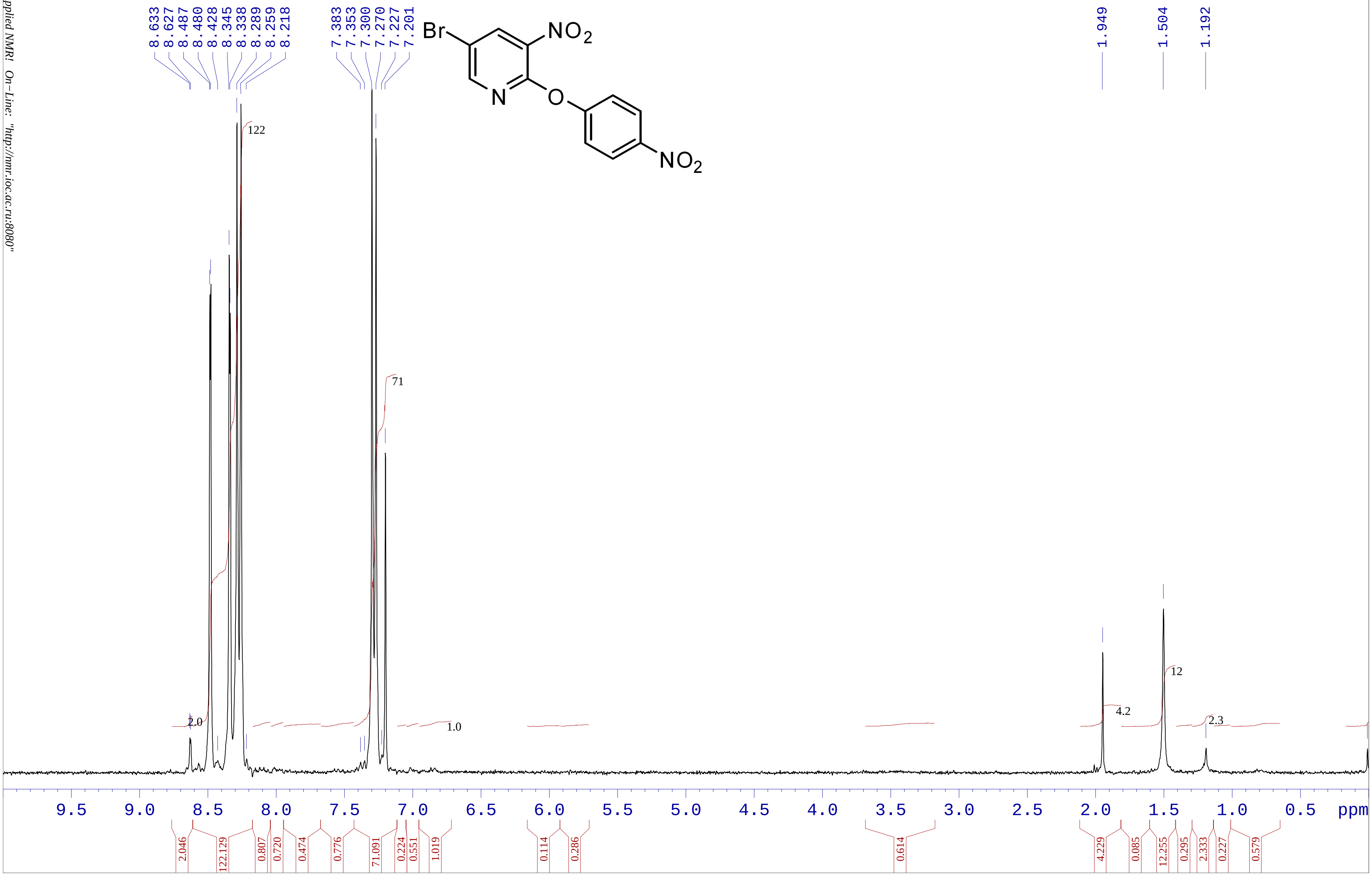

© Zelinsky Institute of Organic Chemistry, Moscow; Bruker AV300 SF=75.47 MHz {13C} SI=64K SW=18027 O1=7924 PW=9.0 AQ=0.905 RD=0.80 NS=274 SR=0.00 TE=300K 5 April 2021 Opr: NMR User; Solv: CDCl3;

The Best Applied NMR! On-Line: "http://nmr.ioc.ac.ru:8080"

/LPIK AF−422.13 Kokorekin−20259

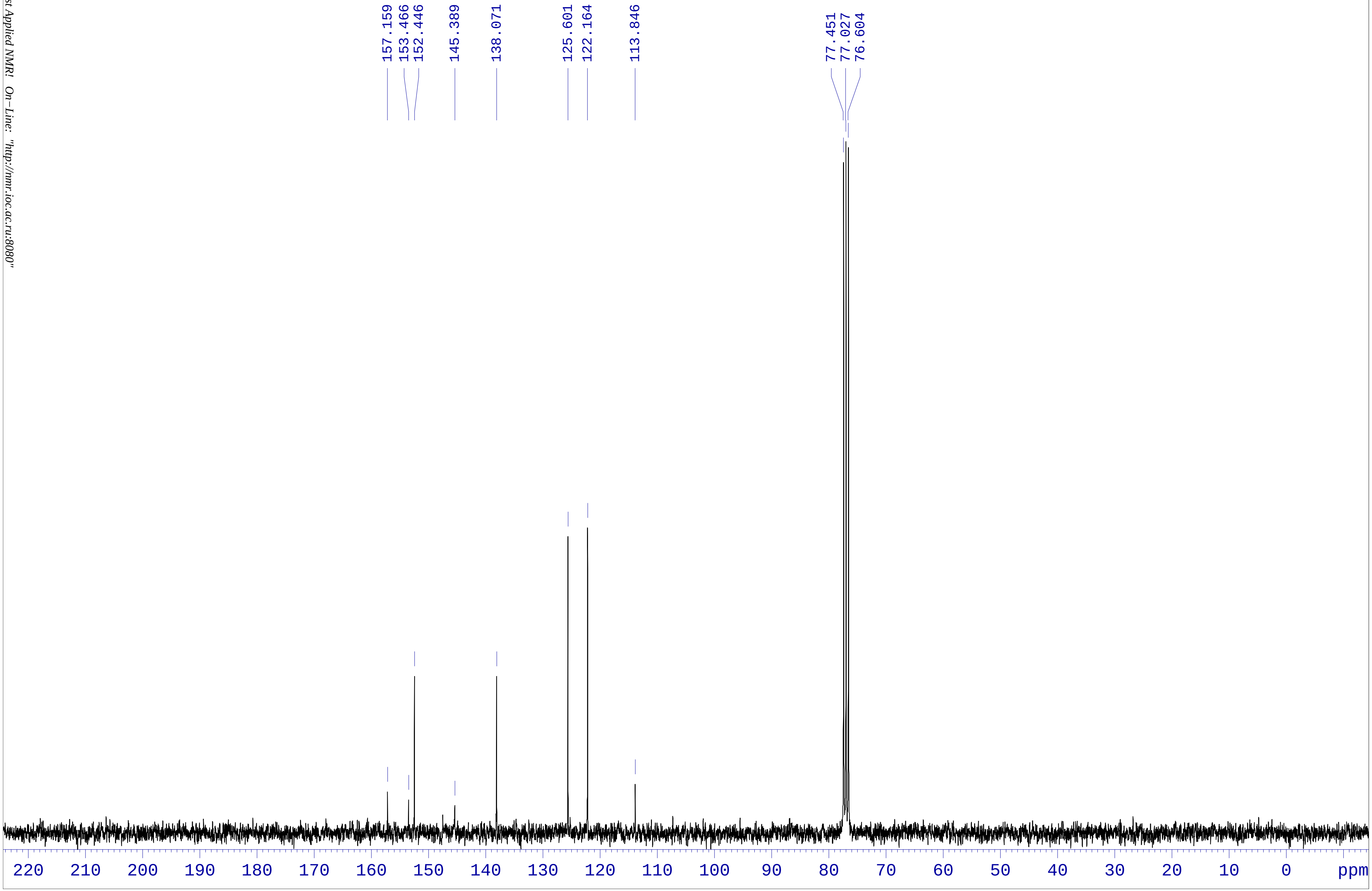

Display Report

Analysis Info

Analysis Name	D:\Data\Kolotyrkina\2021\Bastrakov\0428024.d	Acquisition Date	28.04.2021 12:42:53
Method	tune_50-1600.m	Operator	BDAL@DE
Sample Name	/LPIK AF-422	Instrument / Ser#	micrOTOF 10248
Comment	C11H6BrN3O5 mH 339.9563 clb added CH3OH		

Acquisition Parameter

Source Type	ESI	Ion Polarity	Positive	Set Nebulizer	1.0 Bar
Focus	Not active			Set Dry Heater	200 °C
Scan Begin	50 m/z	Set Capillary	4500 V	Set Dry Gas	4.0 l/min
Scan End	1600 m/z	Set End Plate Offset	-500 V	Set Divert Valve	Waste

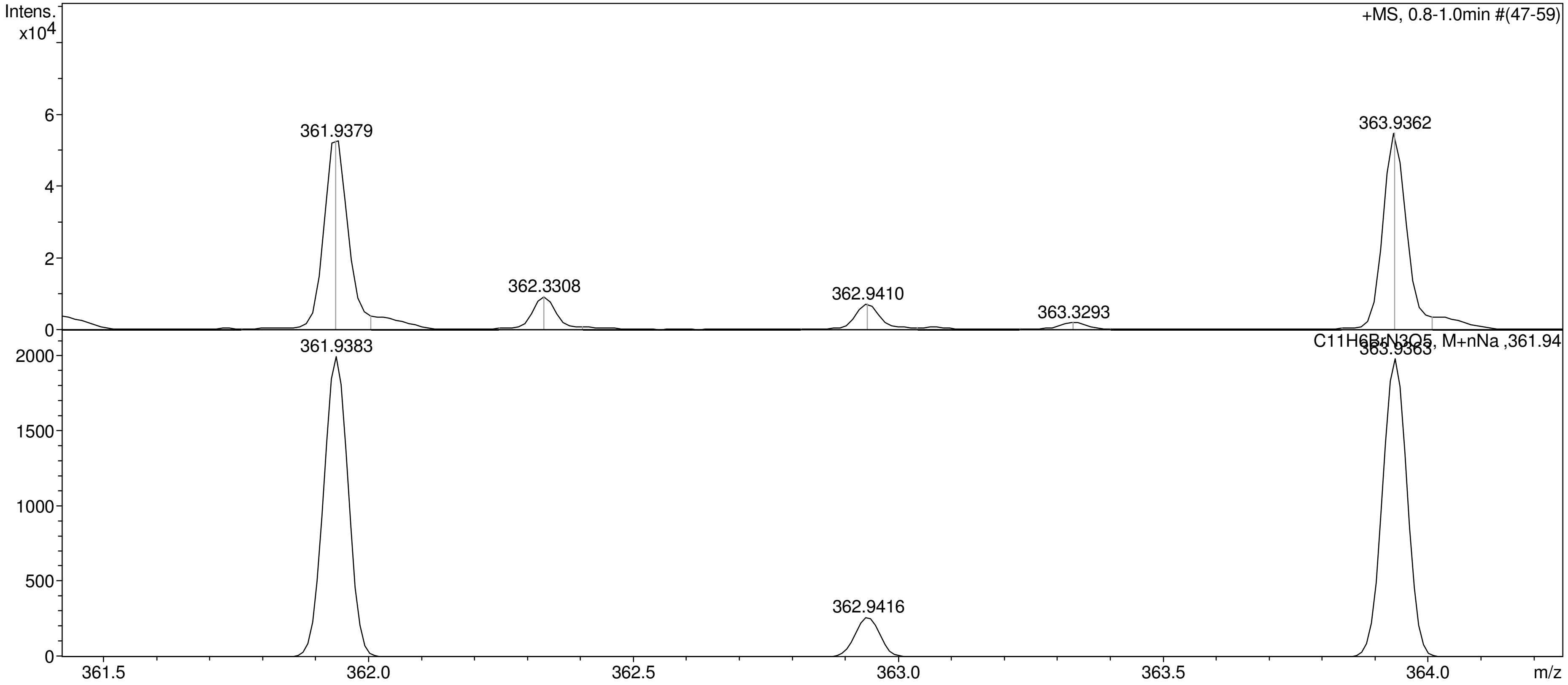

Display Report

Analysis Info

Analysis Name	D:\Data\Kolotyrkina\2021\Bastrakov\0428024.d	Acquisition Date	28.04.2021 12:42:53
Method	tune_50-1600.m	Operator	BDAL@DE
Sample Name	/LPIK AF-422	Instrument / Ser#	micrOTOF 10248
Comment	C11H6BrN3O5 mH 339.9563 clb added CH3OH		

Acquisition Parameter

Source Type	ESI	Ion Polarity	Positive	Set Nebulizer	1.0 Bar
Focus	Not active			Set Dry Heater	200 °C
Scan Begin	50 m/z	Set Capillary	4500 V	Set Dry Gas	4.0 l/min
Scan End	1600 m/z	Set End Plate Offset	-500 V	Set Divert Valve	Waste

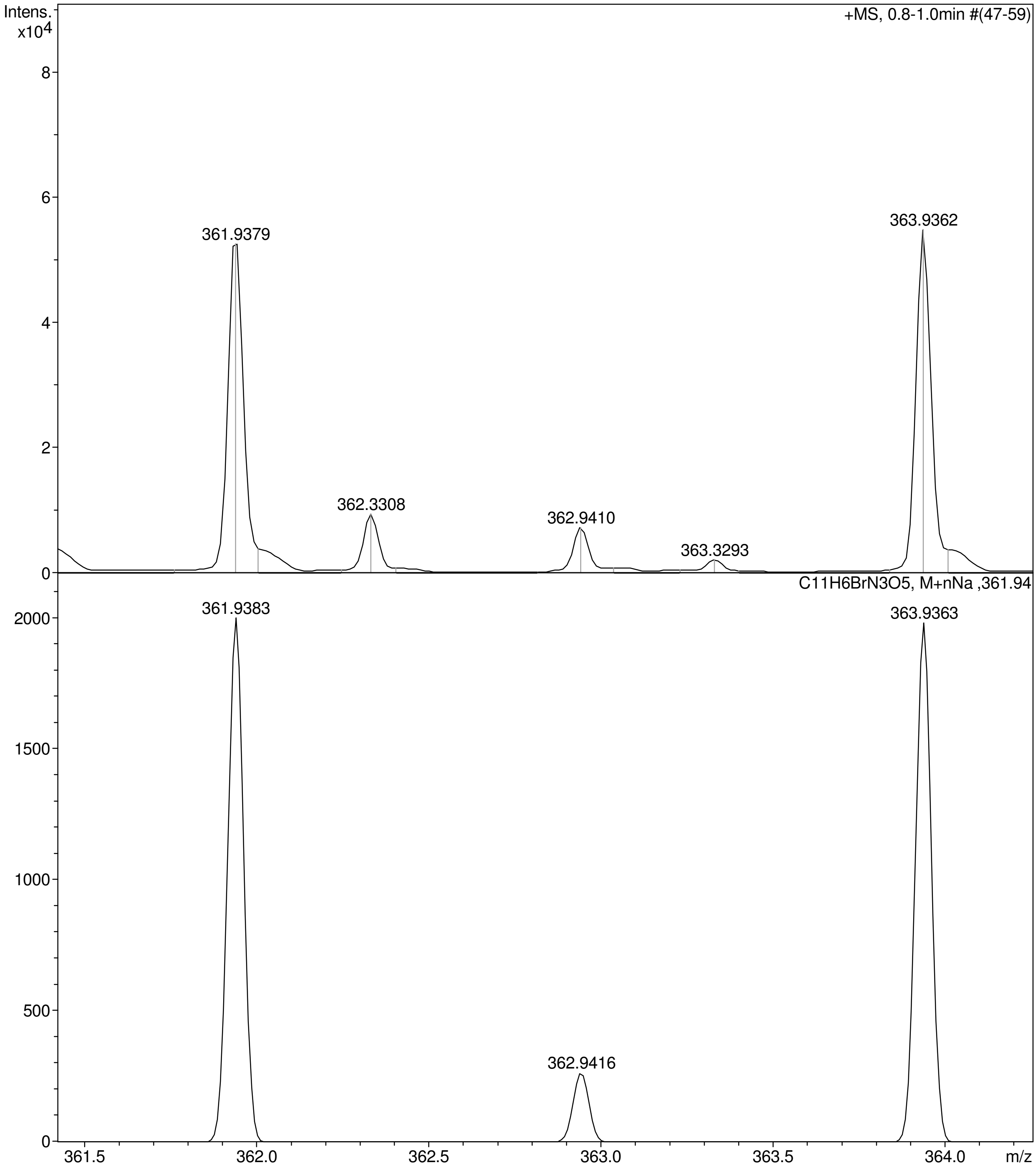

© Zelinsky Institute of Organic Chemistry, Moscow; Bruker AM300 SF=300.13 MHz {1H} SI=16K SW=6010 O1=2401 PW=9.0 AQ=1.351 RD=1.00 NS=1 SR=24.66 TE=298K 31 March 2021 Opr: Shulishov Eu.V.; Solv: CDCl3;

The Best Applied NMR! On−Line: "http://nmr.ioc.ac.ru:8080"

/LPIK AF_423

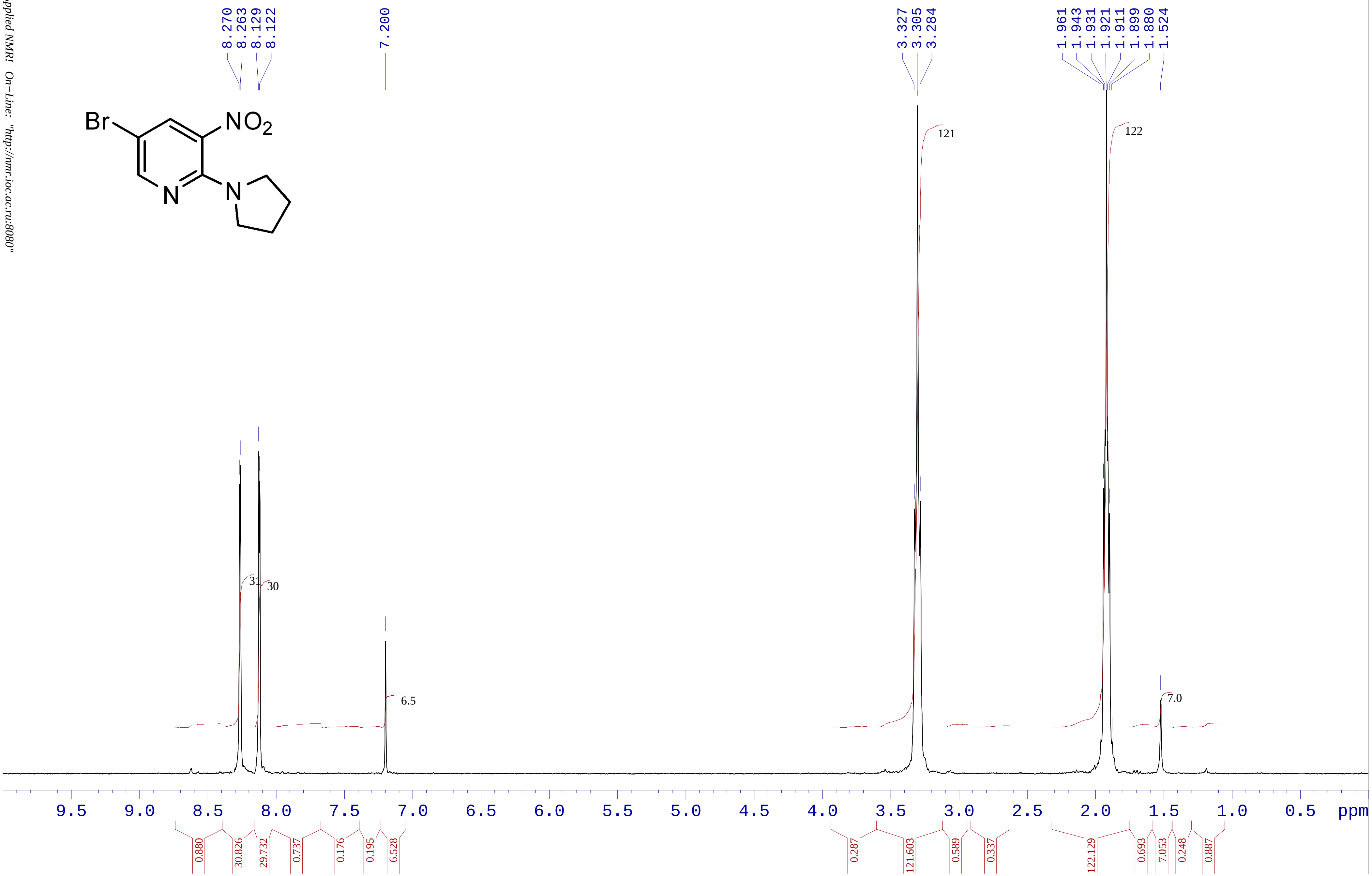

© Zelinsky Institute of Organic Chemistry, Moscow; Bruker AV300 SF=75.47 MHz {13C} SI=64K SW=18027 O1=7924 PW=9.0 AQ=0.905 RD=0.80 NS=269 SR=0.00 TE=300K 5 April 2021 Opr: NMR User; Solv: CDCl3;

The Best Applied NMR! On−Line: "http://nmr.ioc.ac.ru:8080"

/LPIK AF−423.13 Kokorekin−20259

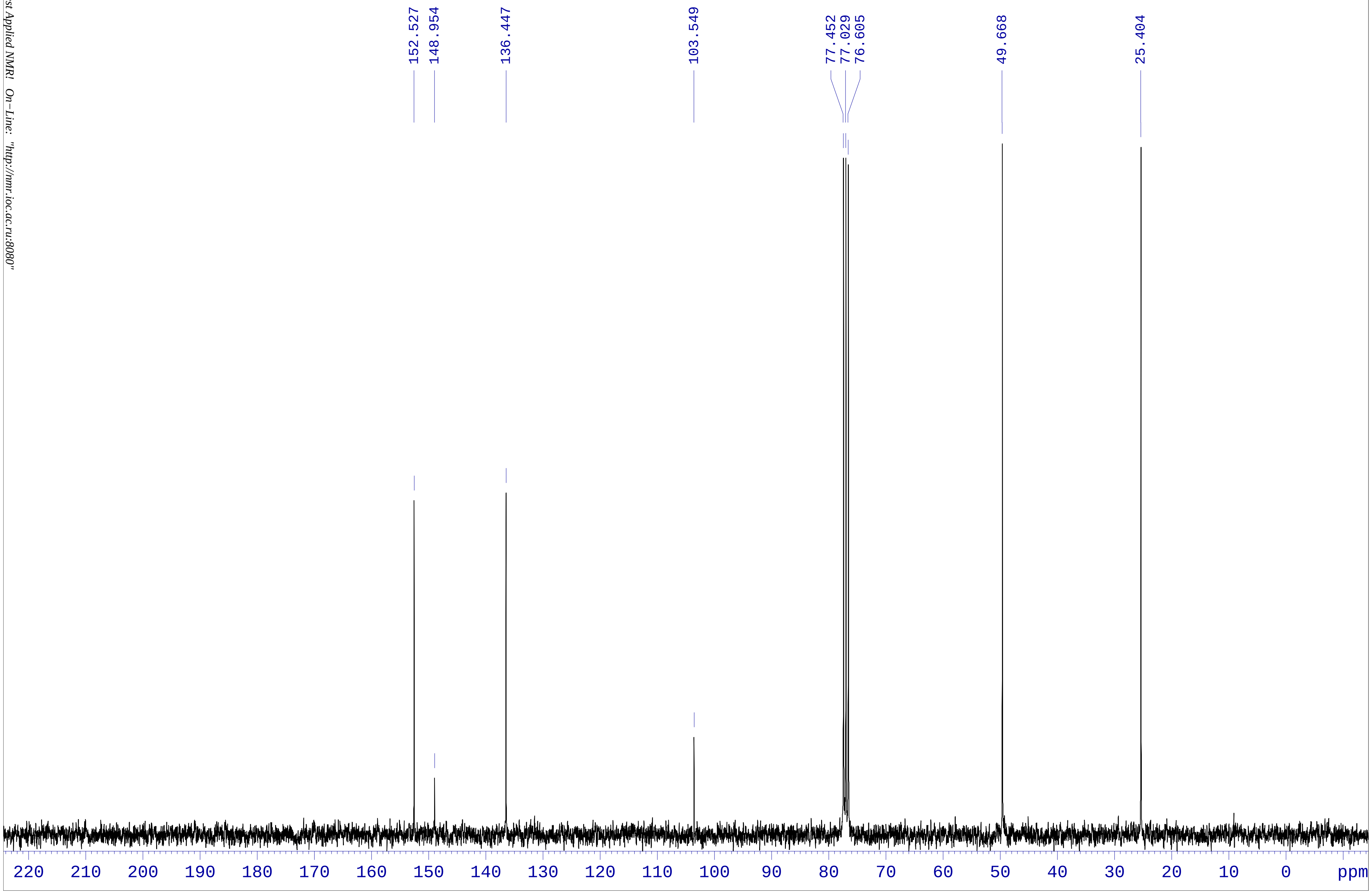

Display Report

Analysis Info

Analysis Name	D:\Data\Kolotyrkina\2021\Bastrakov\0428025.d	Acquisition Date	28.04.2021 12:51:19
Method	tune_50-1600.m	Operator	BDAL@DE
Sample Name	/LPIK AF-423	Instrument / Ser#	micrOTOF 10248
Comment	C9H10BrN3O2 mH 272.0029clb added CH3OH		

Acquisition Parameter

Source Type	ESI	Ion Polarity	Positive	Set Nebulizer	1.0 Bar
Focus	Not active			Set Dry Heater	200 °C
Scan Begin	50 m/z	Set Capillary	4500 V	Set Dry Gas	4.0 l/min
Scan End	1600 m/z	Set End Plate Offset	-500 V	Set Divert Valve	Waste

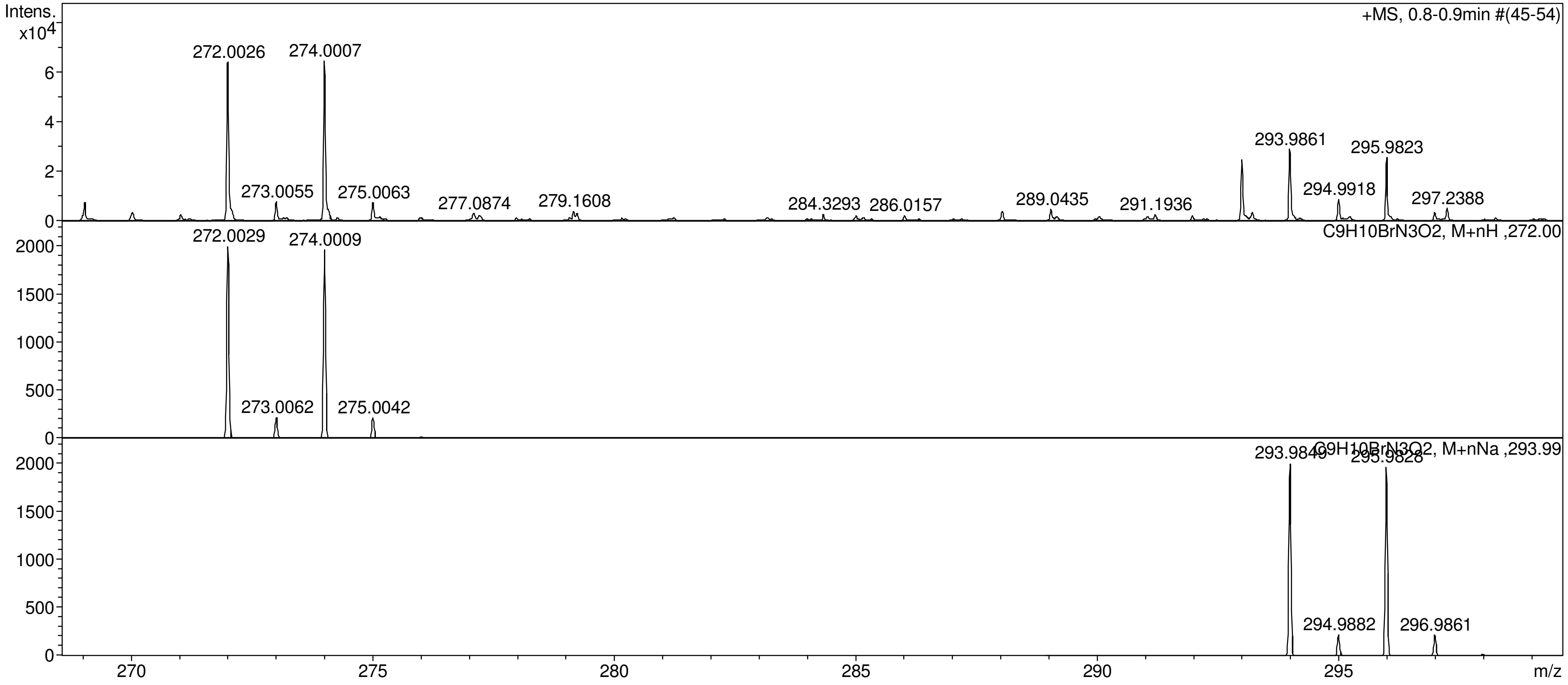

© Zelinsky Institute of Organic Chemistry, Moscow; Bruker AM300 SF=300.13 MHz {1H} SI=16K SW=6010 O1=2401 PW=9.0 AQ=1.351 RD=1.00 NS=1 SR=24.66 TE=298K 1 April 2021 Opr: Shulishov Eu.V.; Solv: CDCl3;

The Best Applied NMR! On−Line: "http://nmr.ioc.ac.ru:8080"

/LPIK AF426

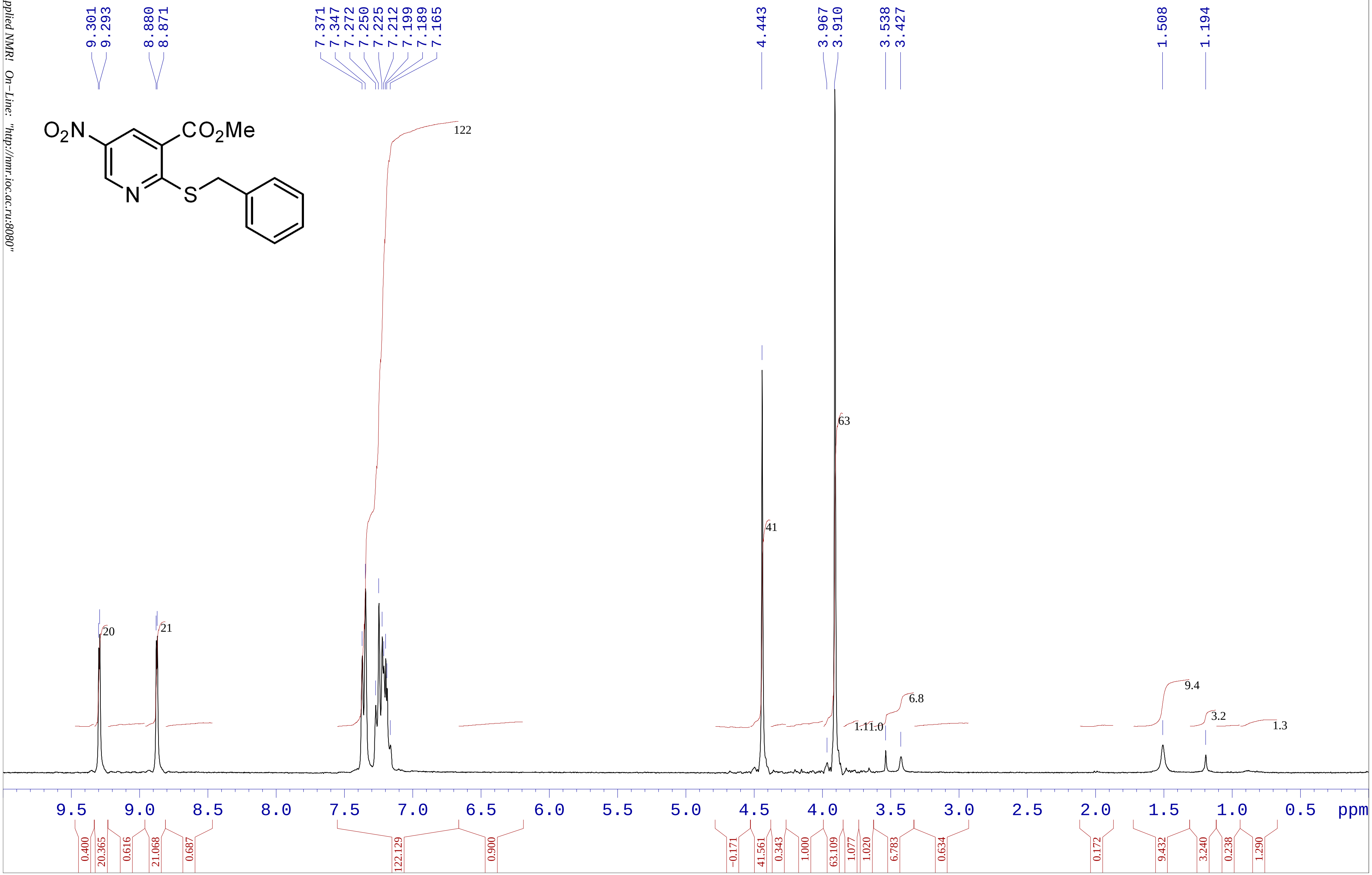

© Zelinsky Institute of Organic Chemistry, Moscow; Bruker AV300 SF=75.47 MHz {13C} SI=64K SW=18027 O1=7924 PW=9.0 AQ=0.905 RD=0.80 NS=201 SR=0.00 TE=300K 5 April 2021 Opr: NMR User; Solv: CDCl3;

The Best Applied NMR! On−Line: "http://nmr.ioc.ac.ru:8080"

/LPIK AF−426.13 Kokorekin−20259

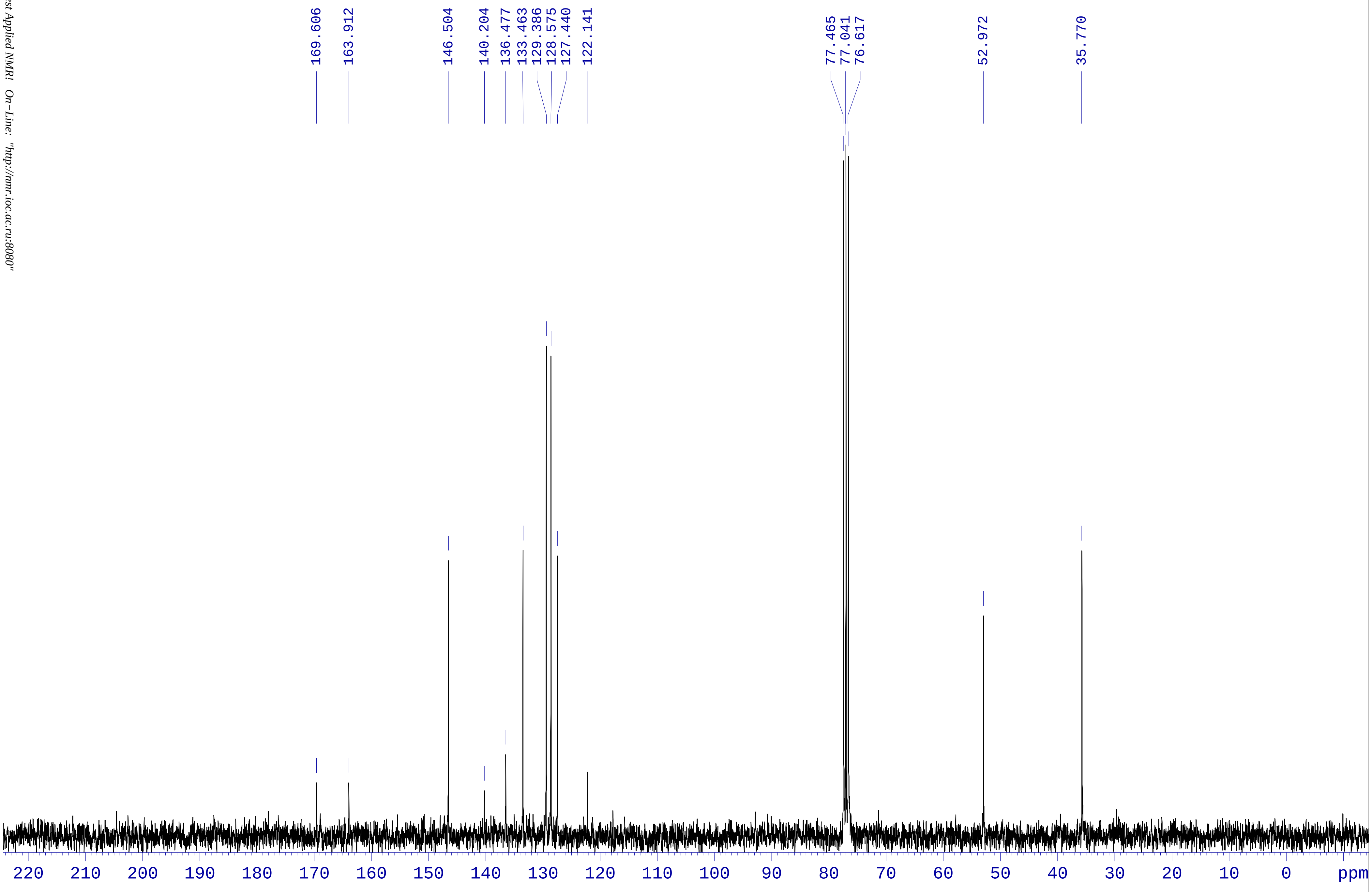

Display Report

Analysis Info

Analysis Name	D:\Data\Kolotyrkina\2021\Bastrakov\0428026.d	Acquisition Date	28.04.2021 12:56:11
Method	tune_50-1600.m	Operator	BDAL@DE
Sample Name	/LPIK AF-426	Instrument / Ser#	micrOTOF 10248
Comment	C14H12N2O4S mH 305.0590 clb added CH3OH		

Acquisition Parameter

Source Type	ESI	Ion Polarity	Positive	Set Nebulizer	1.0 Bar
Focus	Not active			Set Dry Heater	200 °C
Scan Begin	50 m/z	Set Capillary	4500 V	Set Dry Gas	4.0 l/min
Scan End	1600 m/z	Set End Plate Offset	-500 V	Set Divert Valve	Waste

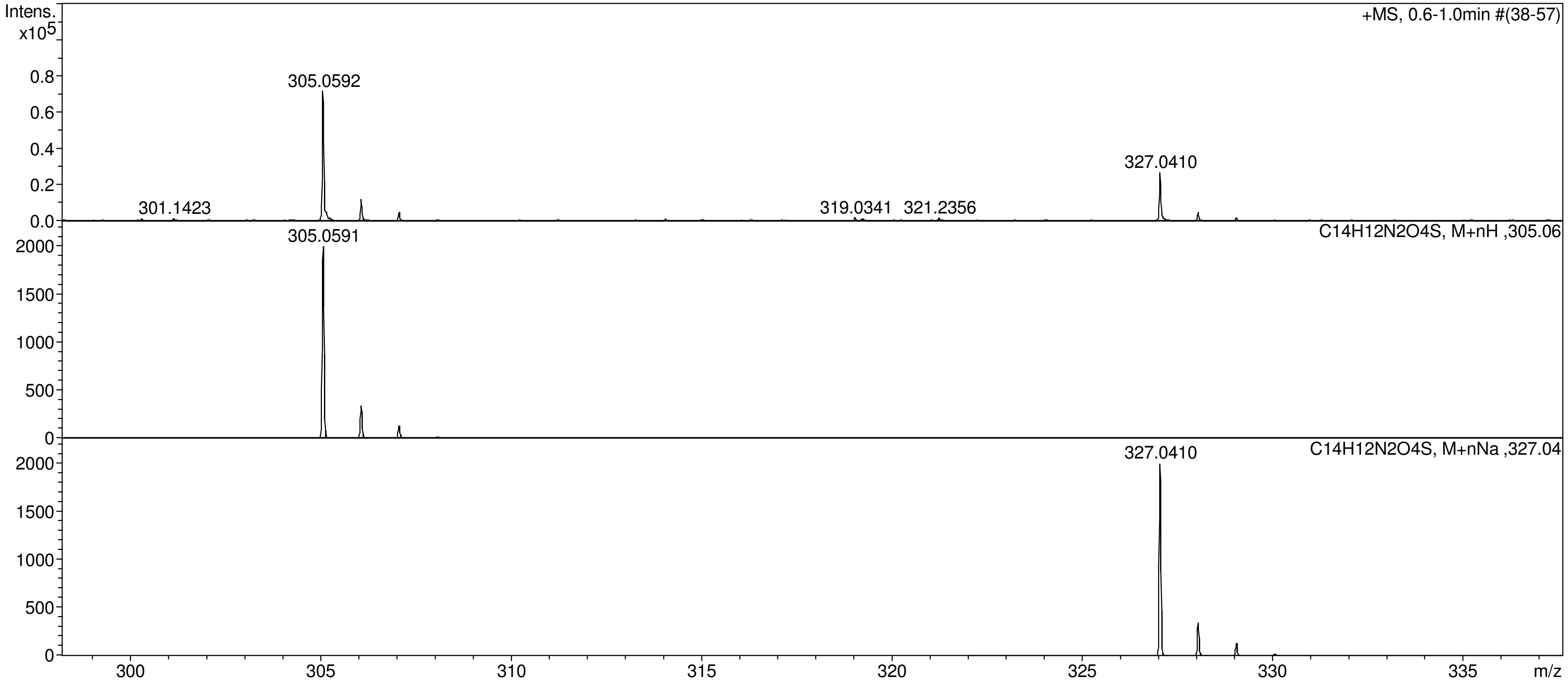

© Zelinsky Institute of Organic Chemistry, Moscow; Bruker AM300 SF=300.13 MHz {1H} SI=16K SW=6010 O1=2401 PW=9.0 AQ=1.351 RD=1.00 NS=1 SR=24.66 TE=298K 1 April 2021 Opr: Shulishov Eu.V.; Solv: CDCl3;

The Best Applied NMR! On-Line: "http://nmr.ioc.ac.ru:8080"

/LPIK AF427

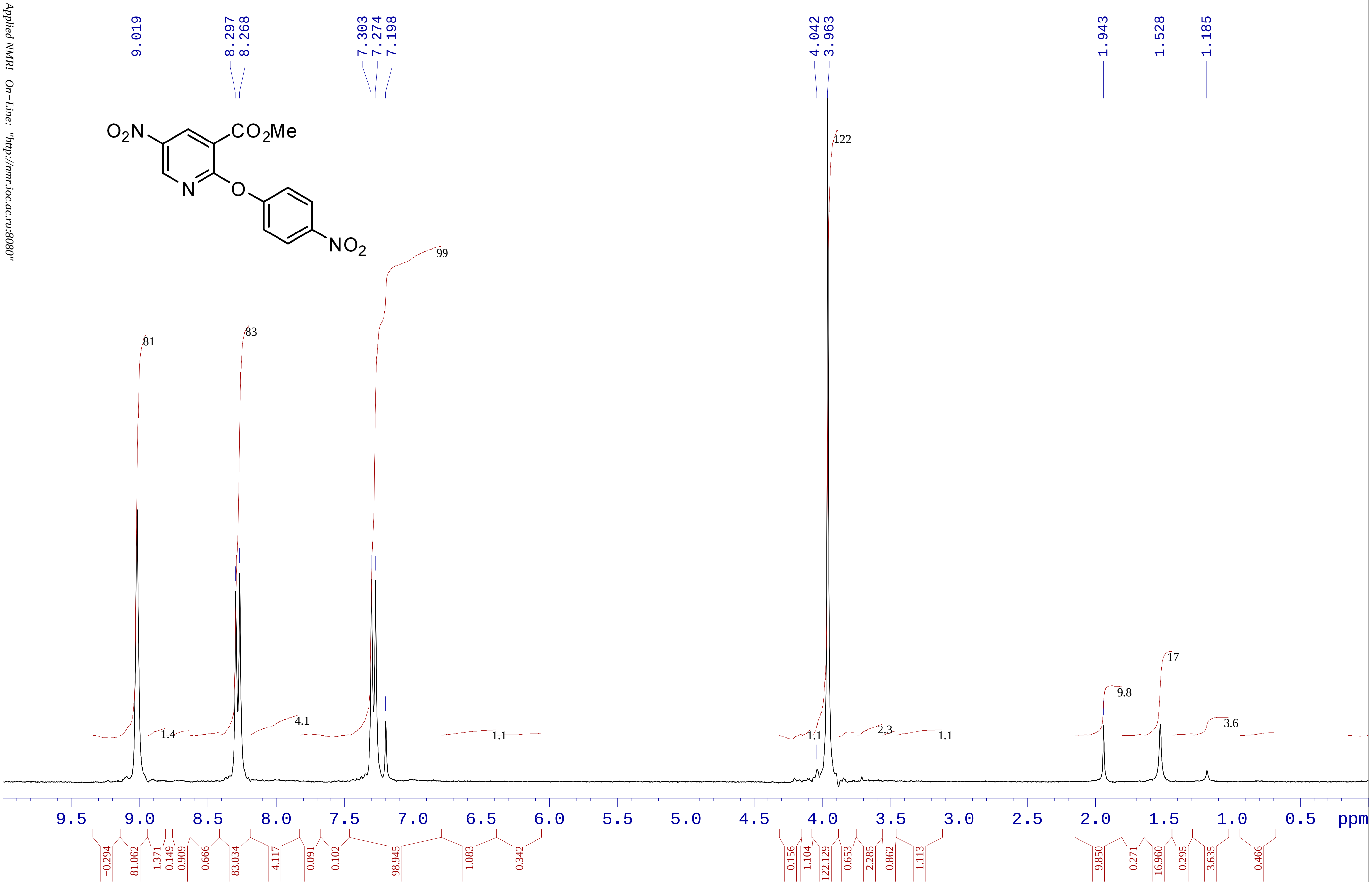

© Zelinsky Institute of Organic Chemistry, Moscow; Bruker AV300 SF=75.47 MHz {13C} SI=64K SW=18027 O1=7924 PW=9.0 AQ=0.905 RD=0.80 NS=130 SR=0.00 TE=300K 6 April 2021 Opr: NMR User; Solv: CDCl3;

The Best Applied NMR! On−Line: "http://nmr.ioc.ac.ru:8080"

/LPIK AF−427.13 Kokorekin−20259

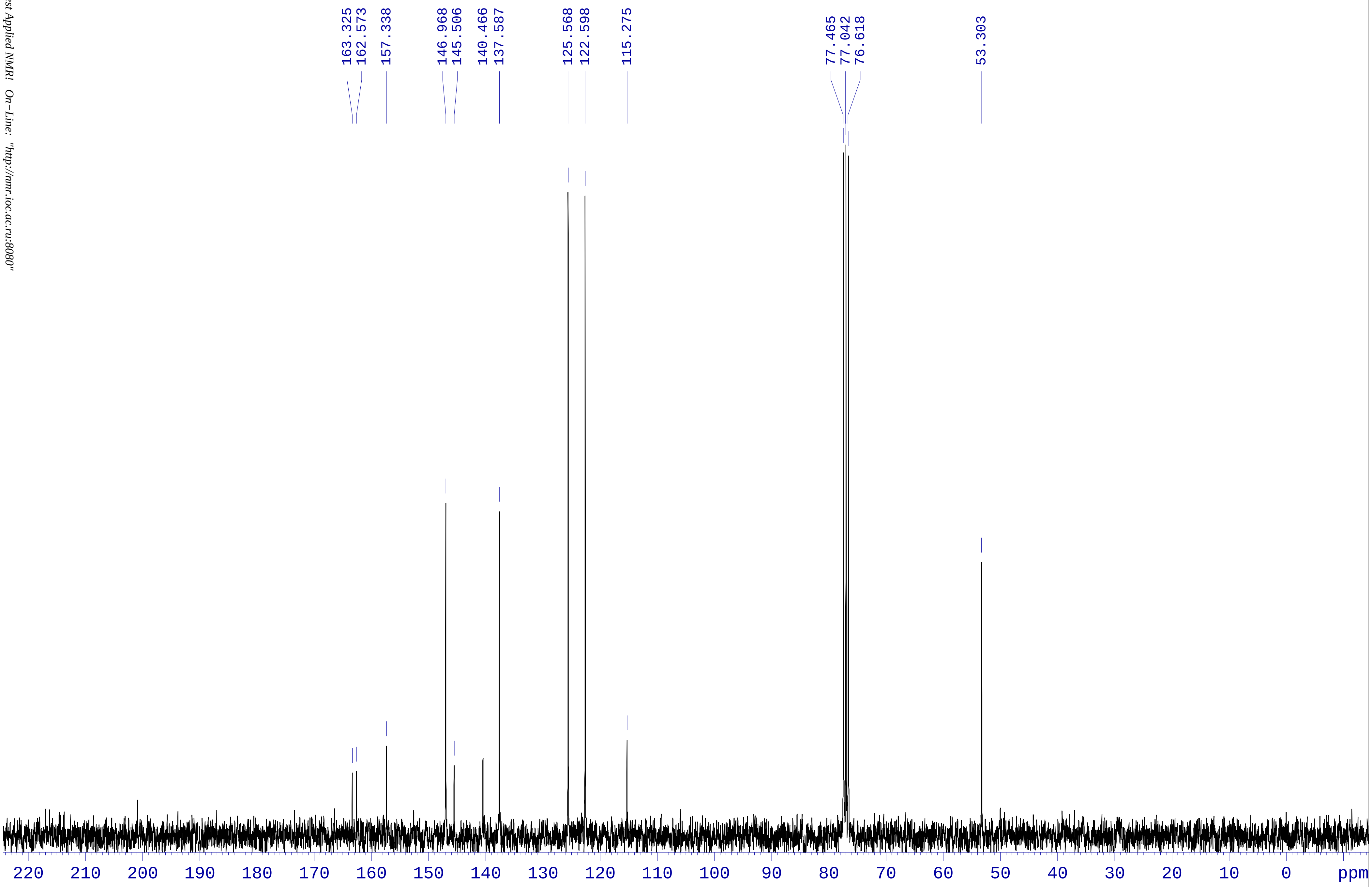

Display Report

Analysis Info

Analysis Name	D:\Data\Kolotyrkina\2021\Bastrakov\0428027.d	Acquisition Date	28.04.2021 13:01:04
Method	tune_50-1600.m	Operator	BDAL@DE
Sample Name	/LPIK AF-427	Instrument / Ser#	micrOTOF 10248
Comment	C13H9N3O7 mH 320.0513 clb added CH3OH		

Acquisition Parameter

Source Type	ESI	Ion Polarity	Positive	Set Nebulizer	1.0 Bar
Focus	Not active			Set Dry Heater	200 °C
Scan Begin	50 m/z	Set Capillary	4500 V	Set Dry Gas	4.0 l/min
Scan End	1600 m/z	Set End Plate Offset	-500 V	Set Divert Valve	Waste

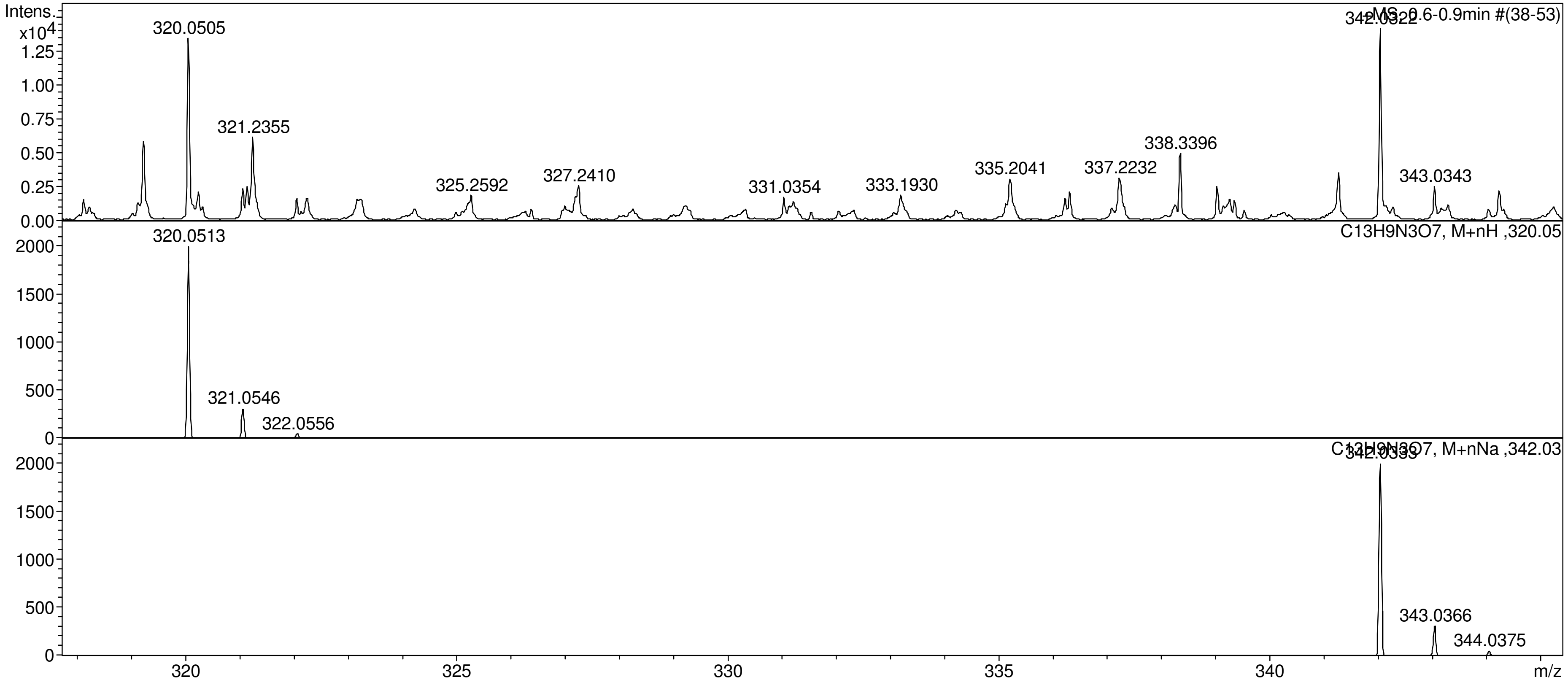

Display Report

Analysis Info

Analysis Name	D:\Data\Kolotyrkina\2021\Bastrakov\0428027.d	Acquisition Date	28.04.2021 13:01:04
Method	tune_50-1600.m	Operator	BDAL@DE
Sample Name	/LPIK AF-427	Instrument / Ser#	micrOTOF 10248
Comment	C13H9N3O7 mH 320.0513 clb added CH3OH		

Acquisition Parameter

Source Type	ESI	Ion Polarity	Positive	Set Nebulizer	1.0 Bar
Focus	Not active			Set Dry Heater	200 °C
Scan Begin	50 m/z	Set Capillary	4500 V	Set Dry Gas	4.0 l/min
Scan End	1600 m/z	Set End Plate Offset	-500 V	Set Divert Valve	Waste

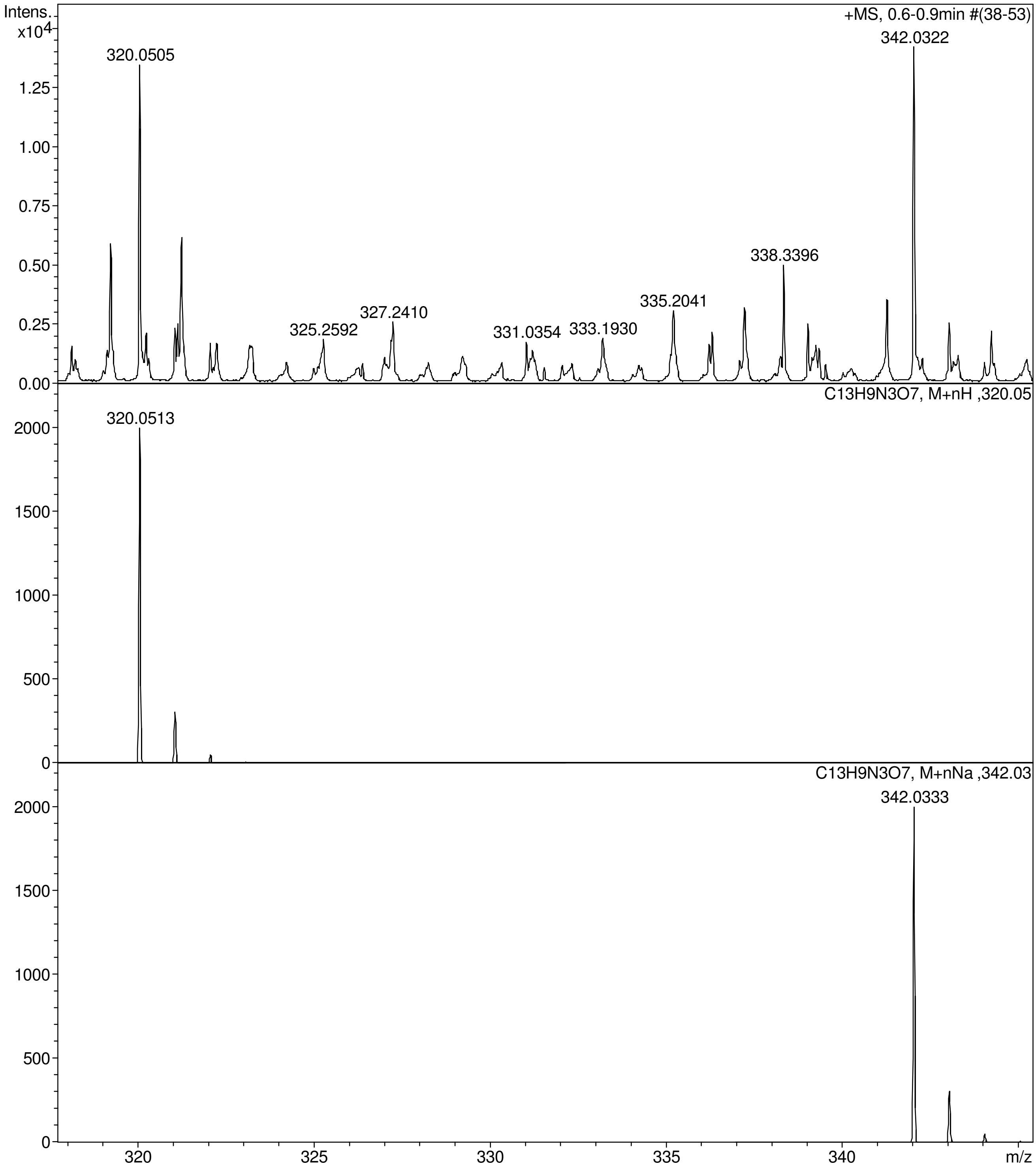

© Zelinsky Institute of Organic Chemistry, Moscow; Bruker AM300 SF=300.13 MHz {1H} SI=16K SW=6010 O1=2401 PW=9.0 AQ=1.351 RD=1.00 NS=1 SR=24.66 TE=298K 1 April 2021 Opr: Shulishov Eu.V.; Solv: CDCl3;

The Best Applied NMR! On-Line: "http://nmr.ioc.ac.ru:8080"

/LPIK AF428

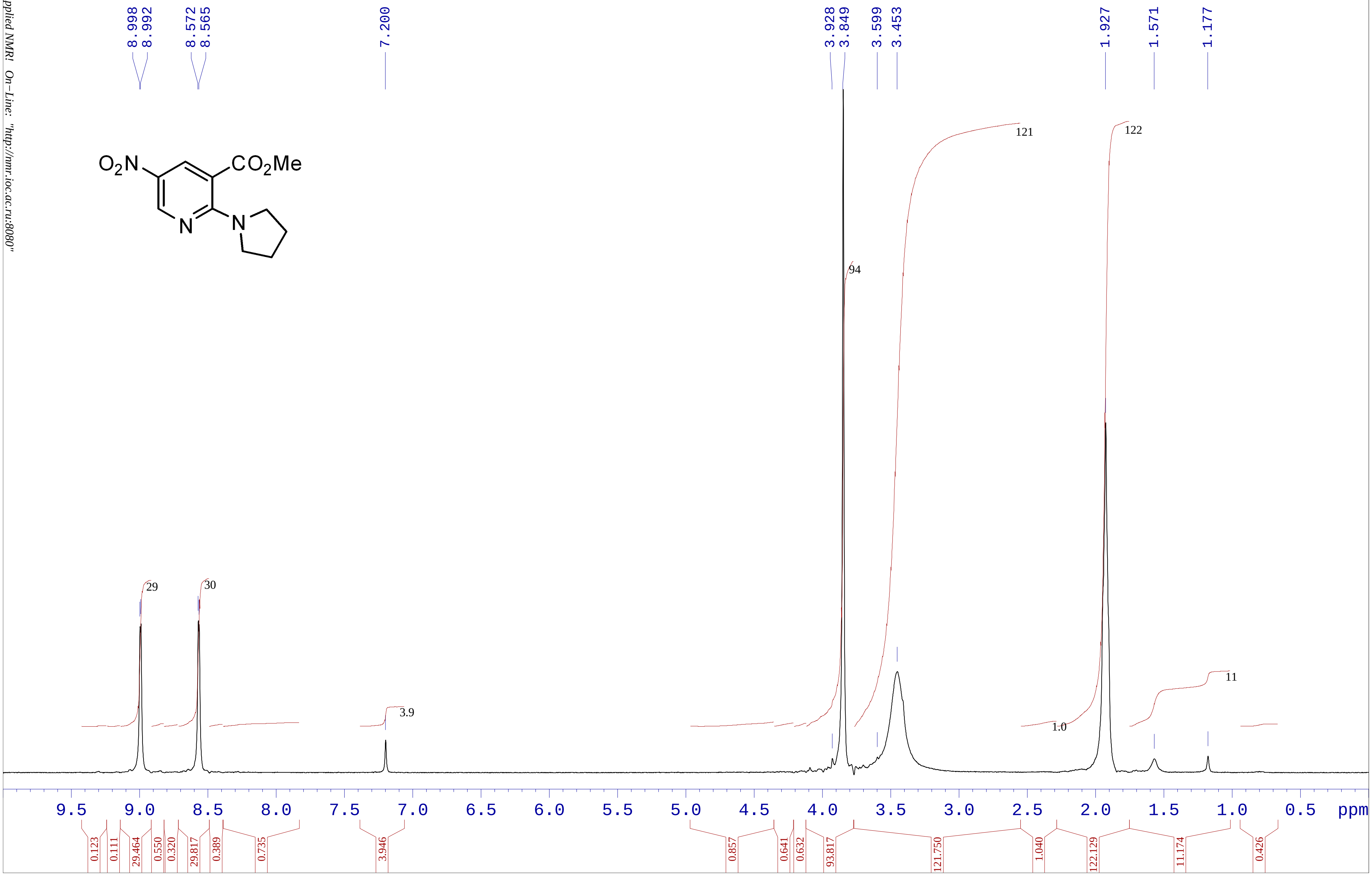

© Zelinsky Institute of Organic Chemistry, Moscow; Bruker AV300 SF=75.47 MHz {13C} SI=64K SW=18027 O1=7924 PW=9.0 AQ=0.905 RD=0.80 NS=148 SR=0.00 TE=300K 6 April 2021 Opr: NMR User; Solv: CDCl3;

The Best Applied NMR! On−Line: "http://nmr.ioc.ac.ru:8080"

/LPIK AF−428.13 Kokorekin−20259

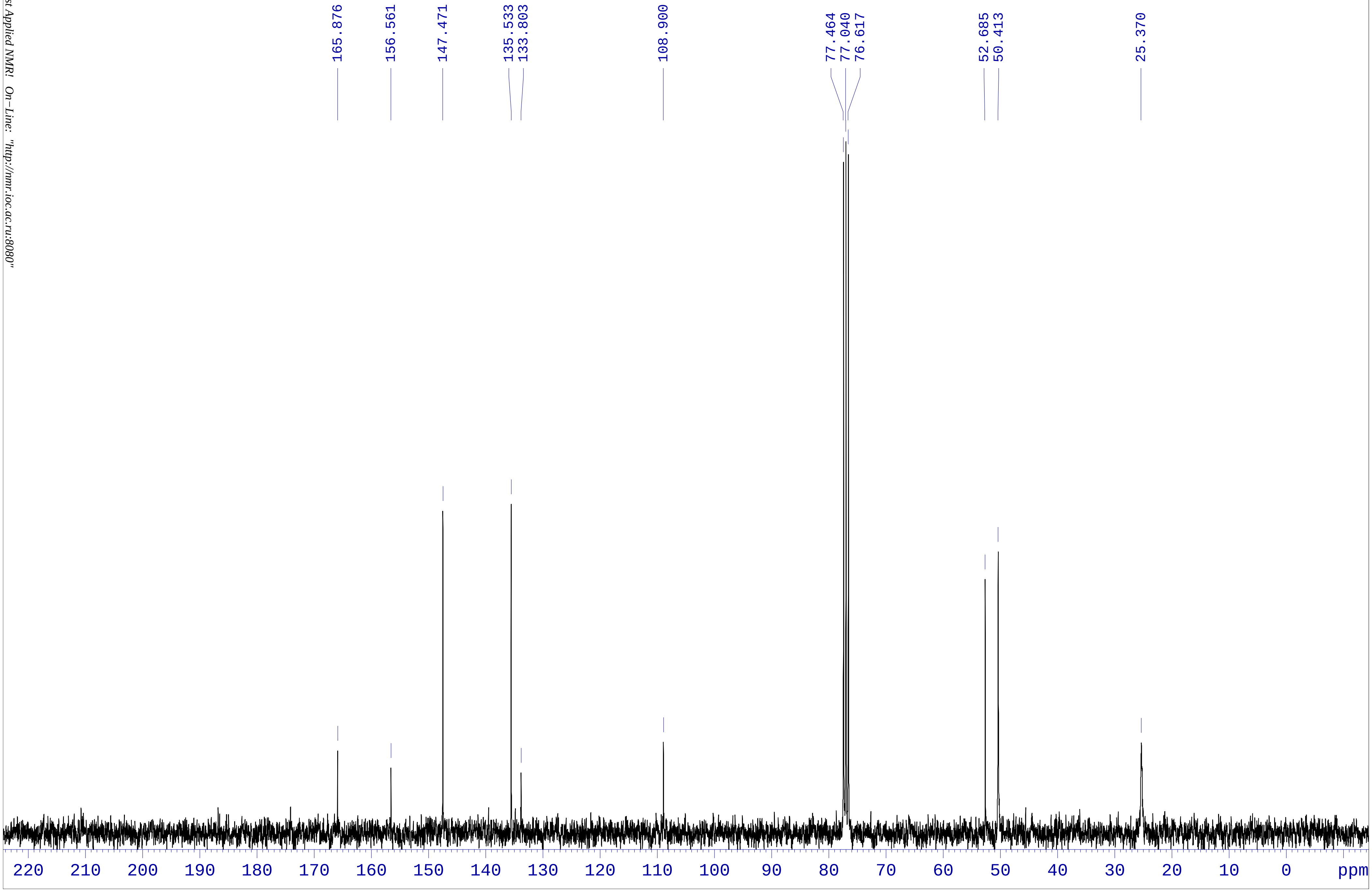

Display Report

Analysis Info

Analysis Name	D:\Data\Kolotyrkina\2021\Bastrakov\0428028.d	Acquisition Date	28.04.2021 13:16:27
Method	tune_50-1600.m	Operator	BDAL@DE
Sample Name	/LPIK AF-428	Instrument / Ser#	micrOTOF 10248
Comment	C11H13N3O4 mH 252.0978 clb added CH3OH		

Acquisition Parameter

Source Type	ESI	Ion Polarity	Positive	Set Nebulizer	1.0 Bar
Focus	Not active			Set Dry Heater	200 °C
Scan Begin	50 m/z	Set Capillary	4500 V	Set Dry Gas	4.0 l/min
Scan End	1600 m/z	Set End Plate Offset	-500 V	Set Divert Valve	Waste

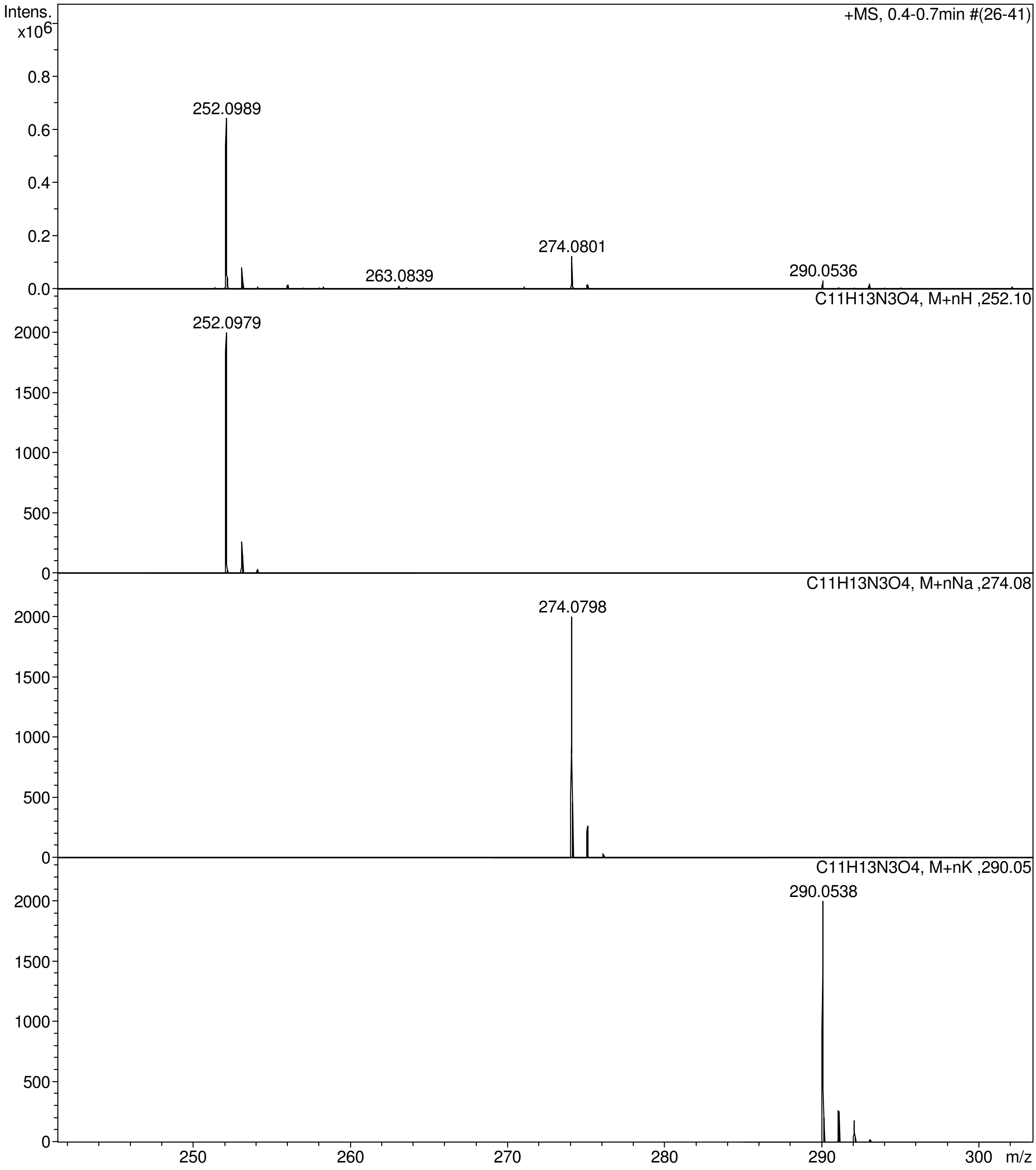

© Zelinsky Institute of Organic Chemistry, Moscow; Bruker AV300 SF=300.13 MHz {1H} SI=64K SW=6010 O1=2101 PW=15.0 AQ=2.715 RD=0.10 NS=1 SR=0.00 TE=300K 9 October 2020 Opr: NMR User; Solv: CDCl3;

The Best Applied NMR! On-Line: "http://nmr.ioc.ac.ru:8080"

/LPIK AF−365.3 Kokorekin−20259

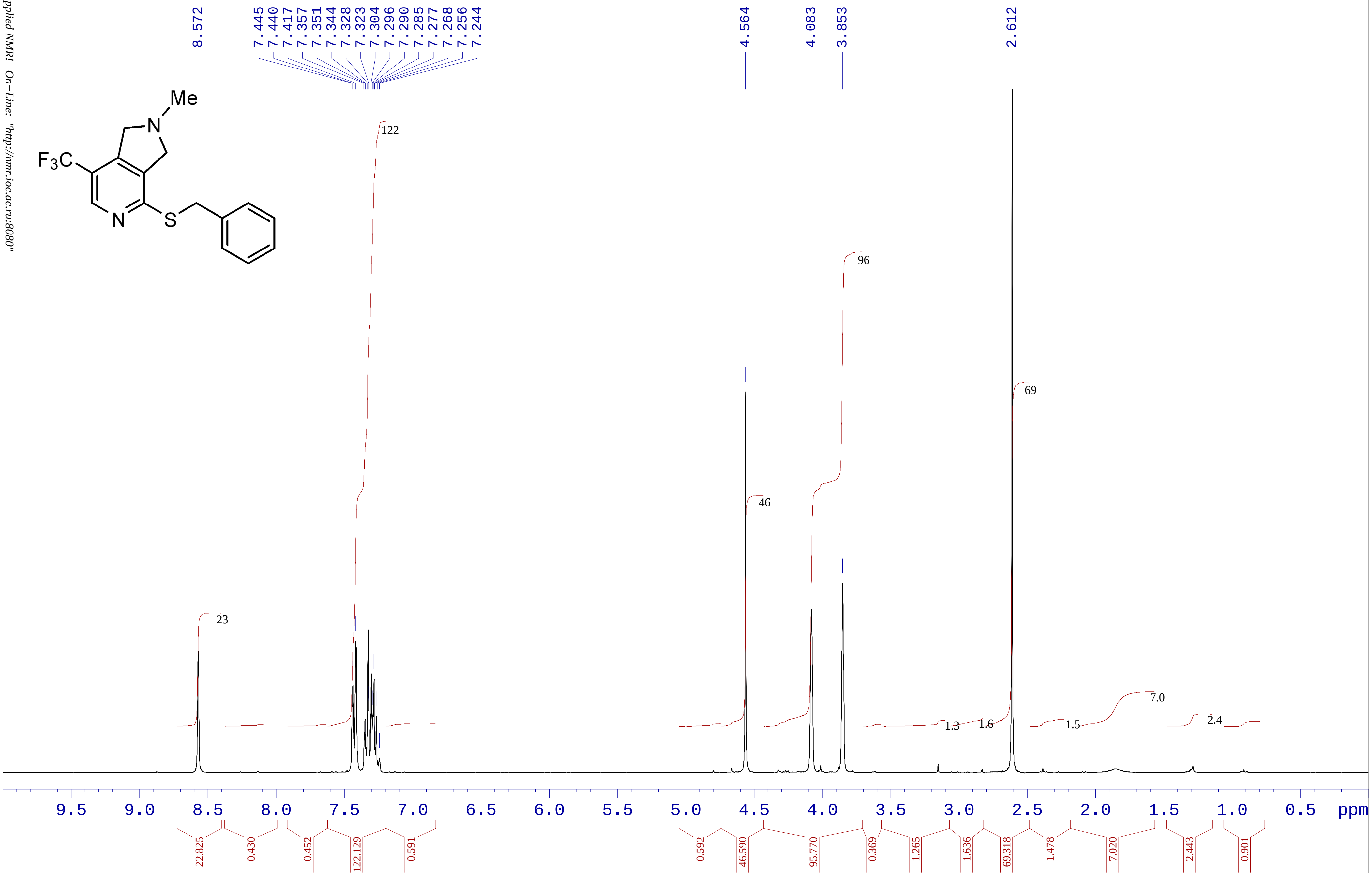

© Zelinsky Institute of Organic Chemistry, Moscow; Bruker AV300 SF=75.47 MHz {13C} SI=64K SW=18027 O1=7924 PW=9.0 AQ=0.905 RD=0.80 NS=181 SR=0.00 TE=300K 12 October 2020 Opr: NMR User; Solv: CDCl3;

/LPIK AF−365.1.13 Kokorekin−20259

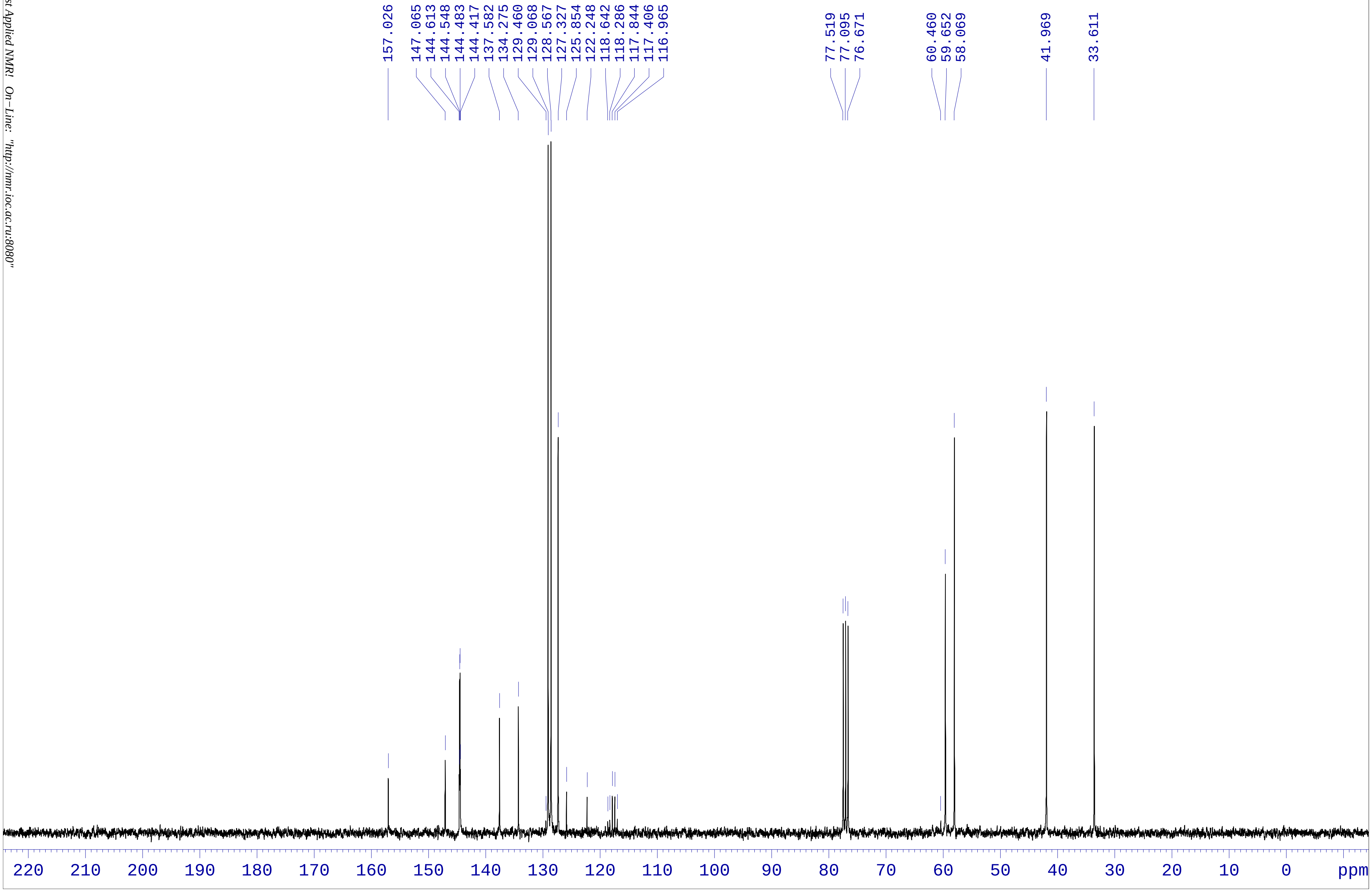

The Best Applied NMR! On−Line: "http://nmr.ioc.ac.ru:8080"

Display Report

Analysis Info

Analysis Name	D:\Data\Kolotyrkina\2021\Bastrakov\0211015.d
Method	tune_50-1600.m
Sample Name	/lpik AF-365
Comment	C16H15F3N2S mH 325.0980 alibrant added CH3OH

Acquisition Date	11.02.2021 11:47:29	
Operator	BDAL@DE	
Instrument / Ser#	micrOTOF	10248

Acquisition Parameter

Source Type	ESI	Ion Polarity	Positive	Set Nebulizer	1.0 Bar
Focus	Not active			Set Dry Heater	200 °C
Scan Begin	50 m/z	Set Capillary	4500 V	Set Dry Gas	4.0 l/min
Scan End	1600 m/z	Set End Plate Offset	-500 V	Set Divert Valve	Waste

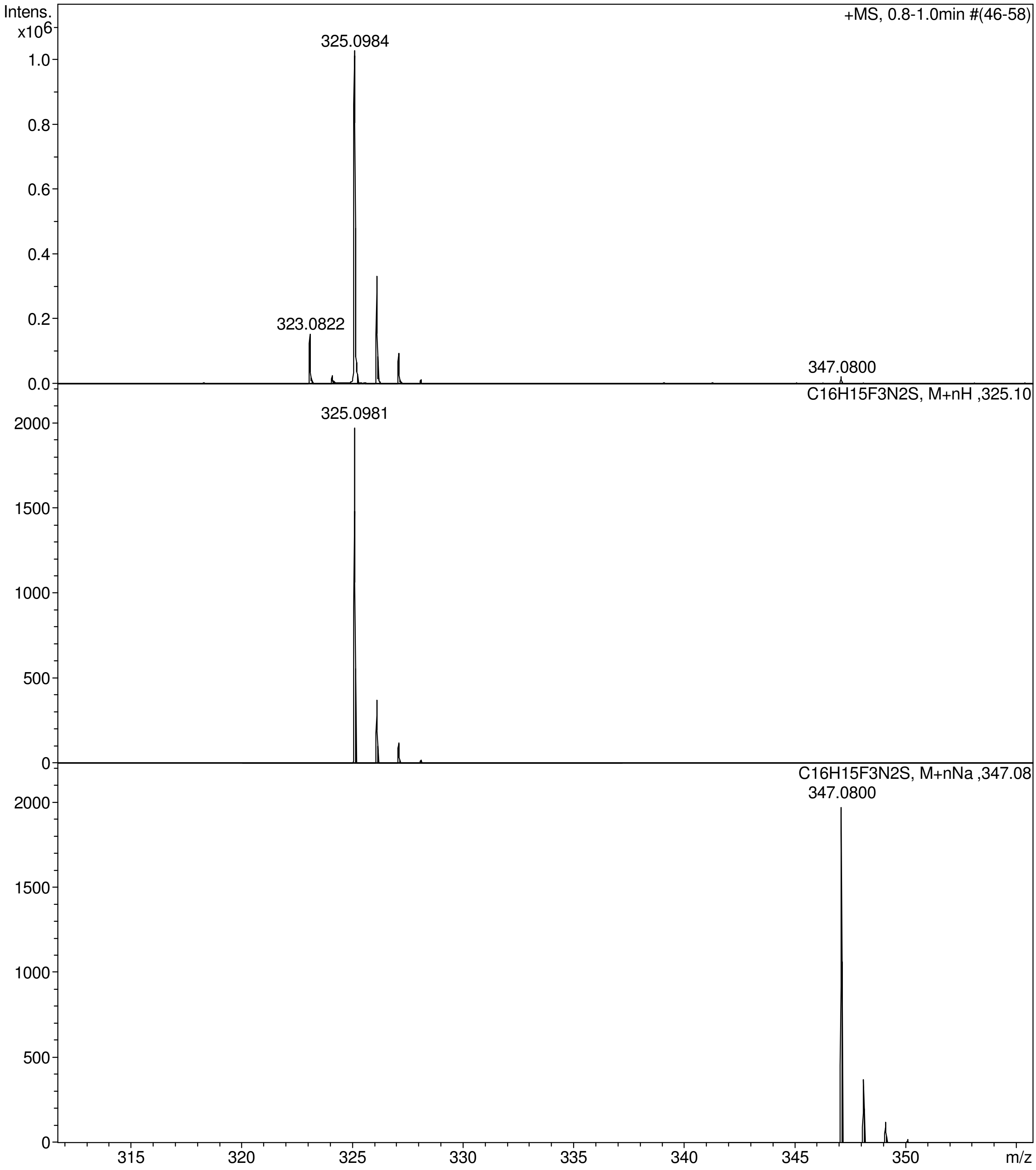

© Zelinsky Institute of Organic Chemistry, Moscow; Bruker AM300 SF=300.13 MHz {1H} SI=32K SW=6010 O1=2401 PW=9.0 AQ=1.352 RD=0.00 NS=1 SR=−1.41 TE=296K 13 October 2020 Opr: Daeva E.D.; Solv: CDCl3;

The Best Applied NMR! On−Line: "http://nmr.ioc.ac.ru:8080"

/LPIK AF−367.R

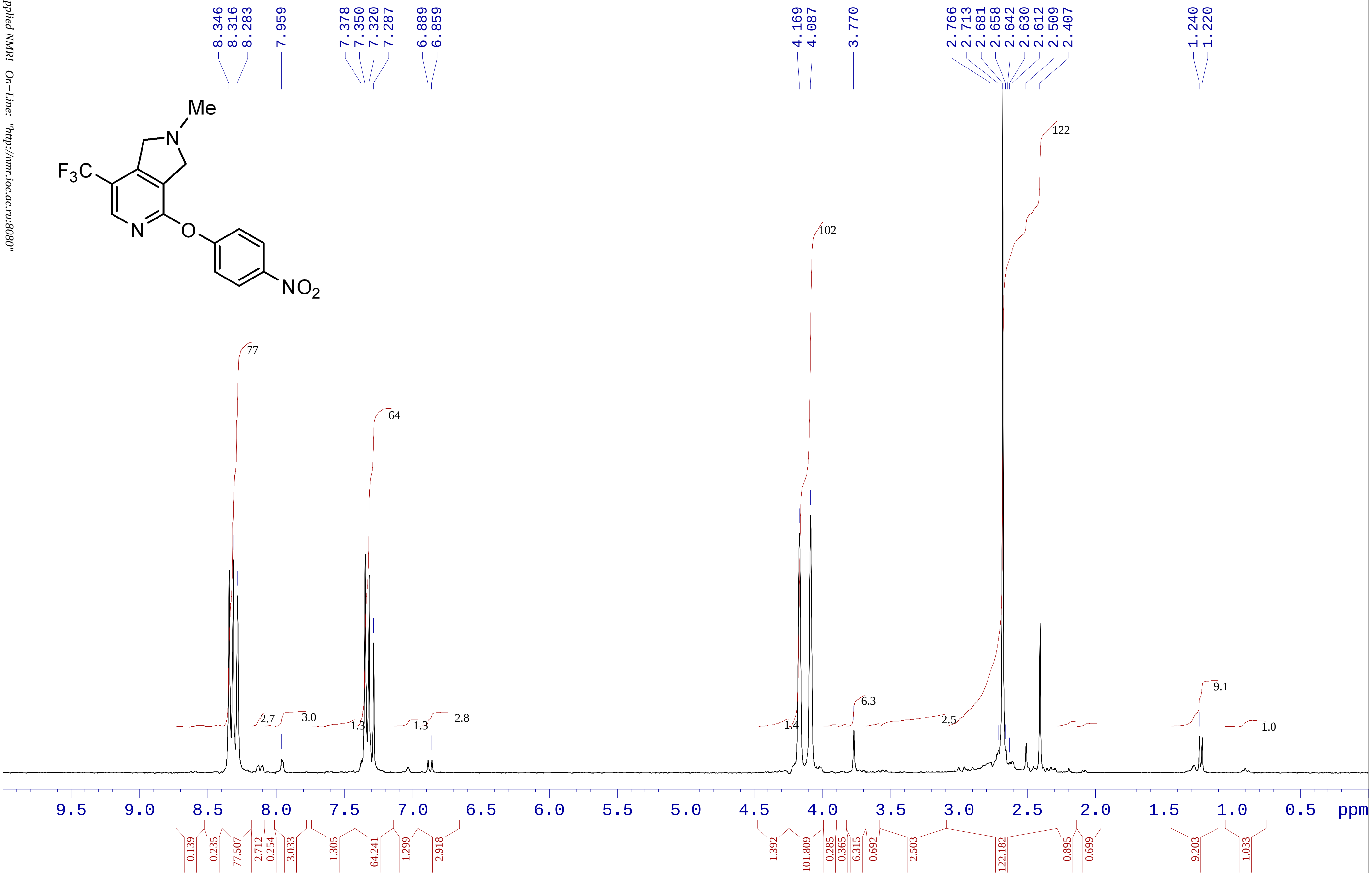

© Zelinsky Institute of Organic Chemistry, Moscow; Bruker AM300 SF=75.49 MHz {13C} SI=32K SW=17855 O1=7549 PW=10.0 AQ=1.143 RD=0.80 NS=1024 SR=0.00 TE=298K 2 November 2020 Opr: NMR User; Solv: CDCl3;

The Best Applied NMR! On−Line: "http://nmr.ioc.ac.ru:8080"

/LPIK AF−367.C13 CDCl3 13C 20mg 1024scans

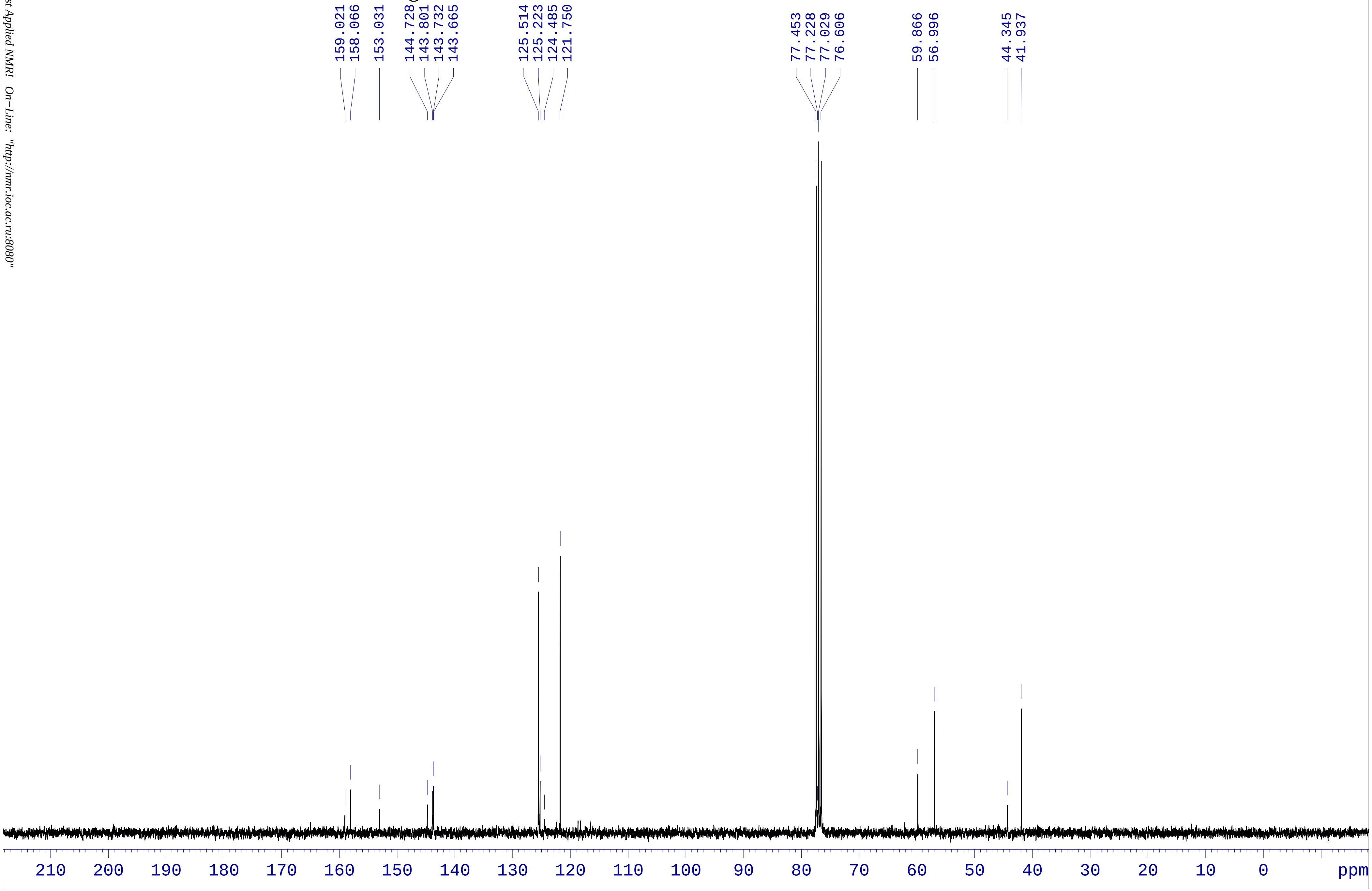

Display Report

Analysis Info

Analysis Name	D:\Data\Kolotyrkina\2021\Bastrakov\0211016.d	Acquisition Date	11.02.2021 11:56:34
Method	tune_50-1600.m	Operator	BDAL@DE
Sample Name	/Ipik AF-367	Instrument / Ser#	micrOTOF 10248
Comment	C15H12F3N3O3 mH 340.0903 alibrant added CH3OH		

Acquisition Parameter

Source Type	ESI	Ion Polarity	Positive	Set Nebulizer	1.0 Bar
Focus	Not active			Set Dry Heater	200 °C
Scan Begin	50 m/z	Set Capillary	4500 V	Set Dry Gas	4.0 l/min
Scan End	1600 m/z	Set End Plate Offset	-500 V	Set Divert Valve	Waste

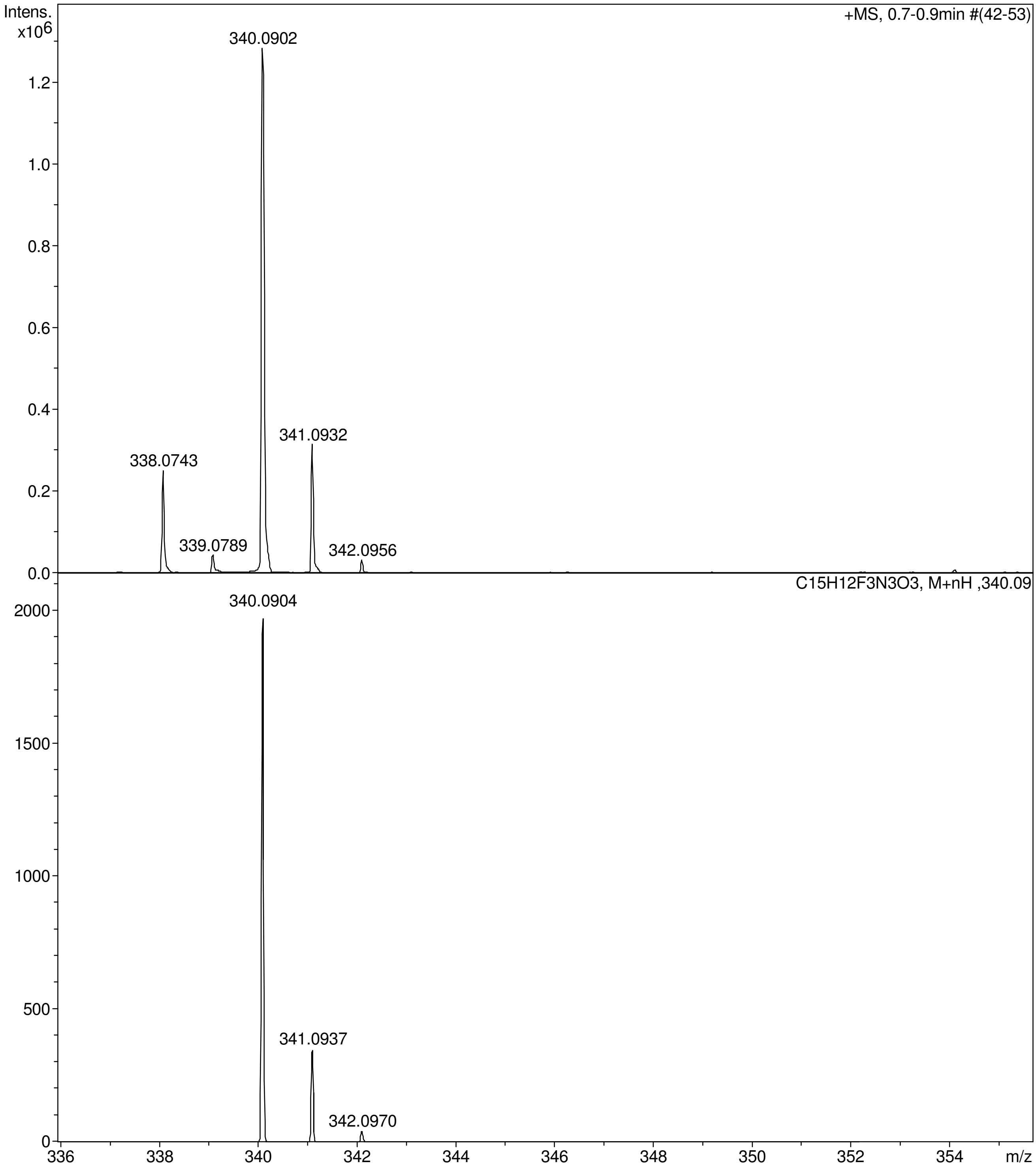

© Zelinsky Institute of Organic Chemistry, Moscow; Bruker AM300 SF=300.13 MHz {1H} SI=16K SW=6010 O1=2401 PW=9.0 AQ=1.352 RD=0.00 NS=1 SR=4.03 TE=301K 16 November 2020 Opr: Daeva E.D.; Solv: CDCl3;

The Best Applied NMR! On−Line: "http://nmr.ioc.ac.ru:8080"

/LPIK AF−388

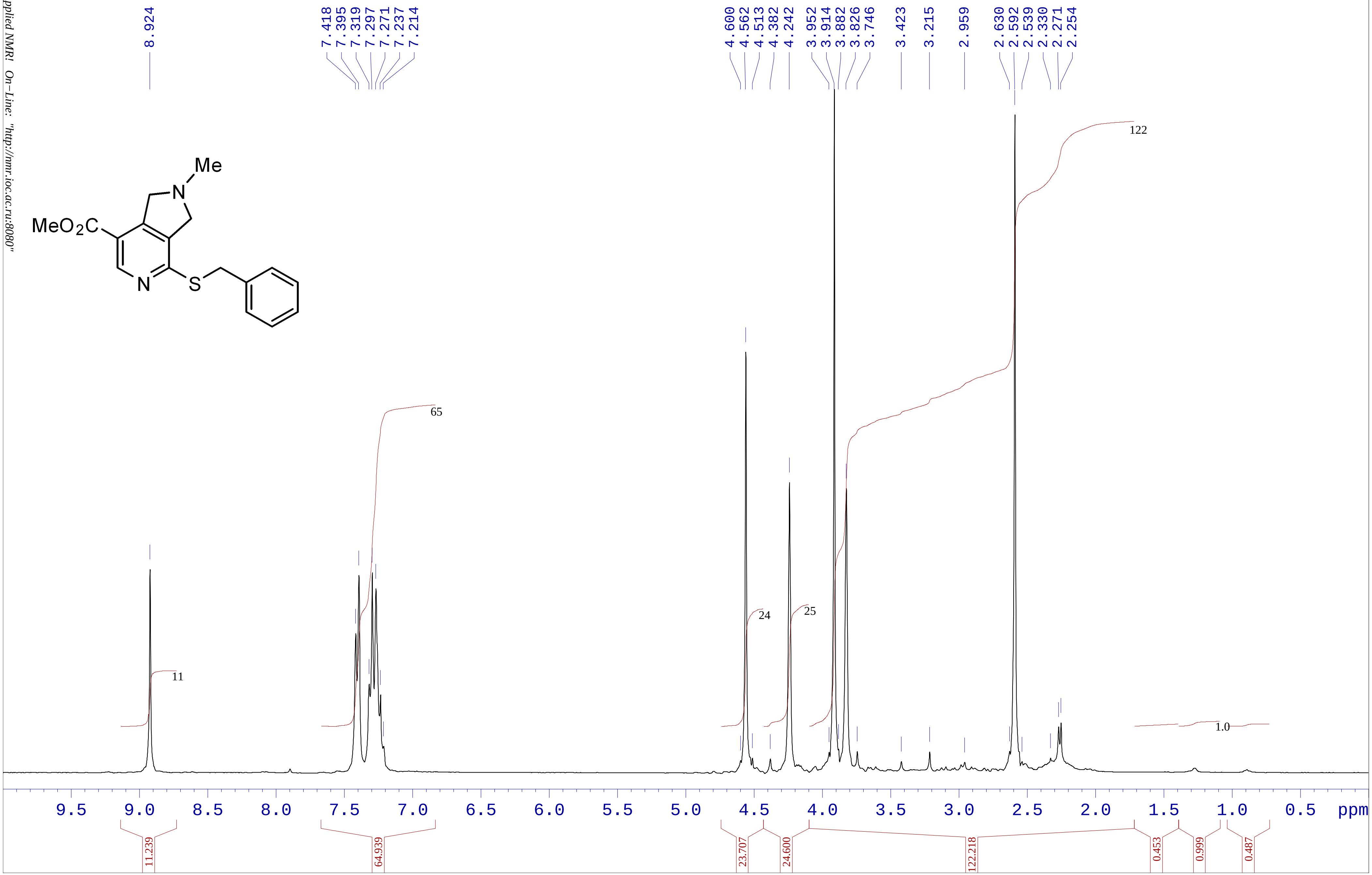

© Zelinsky Institute of Organic Chemistry, Moscow; Bruker AM300 SF=75.49 MHz {13C} SI=32K SW=17855 O1=7549 PW=10.0 AQ=1.143 RD=0.80 NS=512 SR=0.00 TE=298K 17 November 2020 Opr: NMR User; Solv: CDCl3;

The Best Applied NMR! On−Line: "http://nmr.ioc.ac.ru:8080"

/LPIK AF−388.C13 CDCl3 13C 25mg 512scans

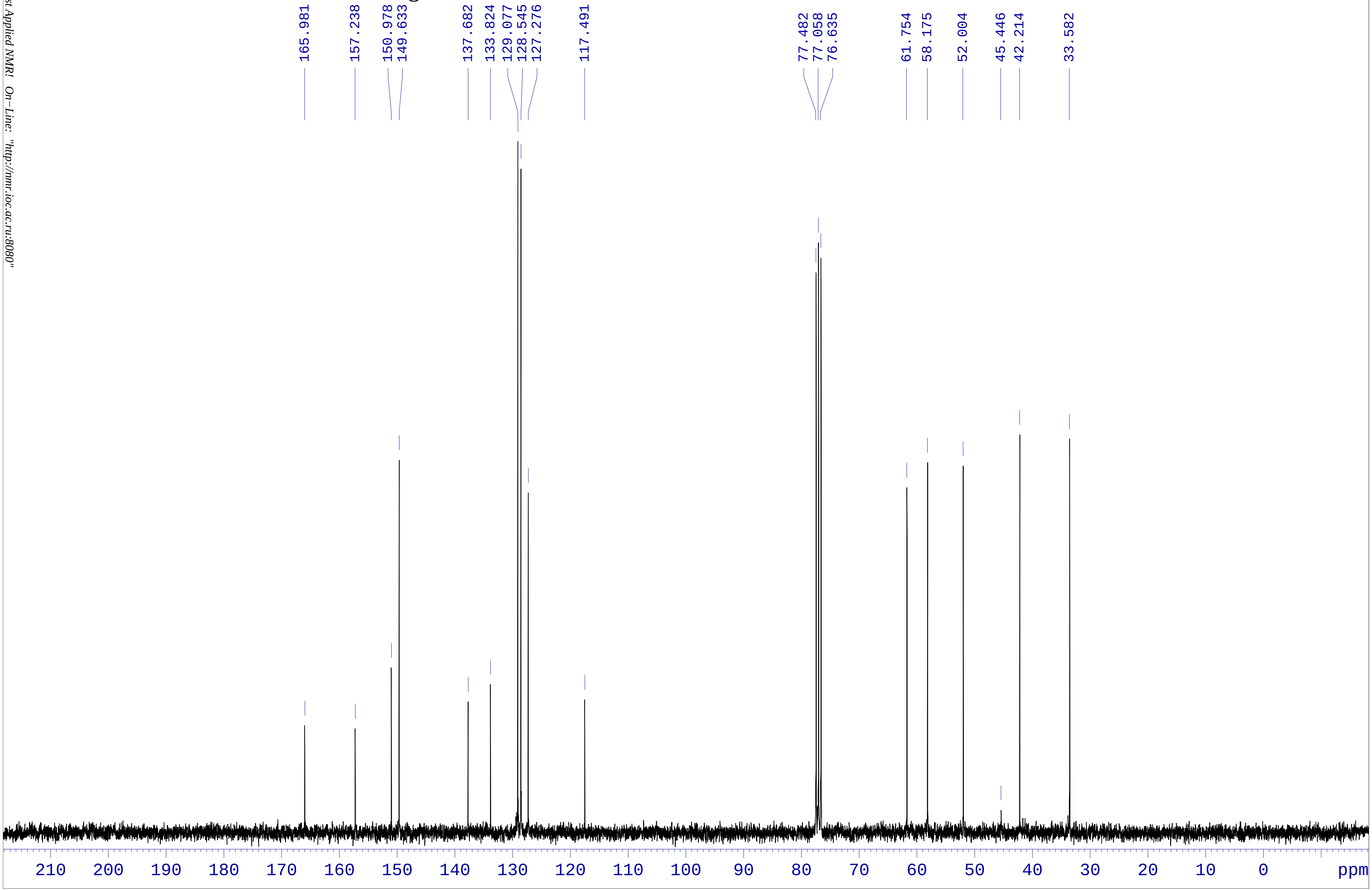

Display Report

Analysis Info

Analysis Name	D:\Data\Kolotyrkina\2021\Bastrakov\0210030.d	Acquisition Date	10.02.2021 21:06:14
Method	tune_50-1600.m	Operator	BDAL@DE
Sample Name	/LPIK AF-388	Instrument / Ser#	micrOTOF 10248
Comment	C17H18N2O2S mH 315.0061 calibrant added CH3CN		

Acquisition Parameter

Source Type	ESI	Ion Polarity	Positive	Set Nebulizer	1.0 Bar
Focus	Not active			Set Dry Heater	200 °C
Scan Begin	50 m/z	Set Capillary	4500 V	Set Dry Gas	4.0 l/min
Scan End	1600 m/z	Set End Plate Offset	-500 V	Set Divert Valve	Waste

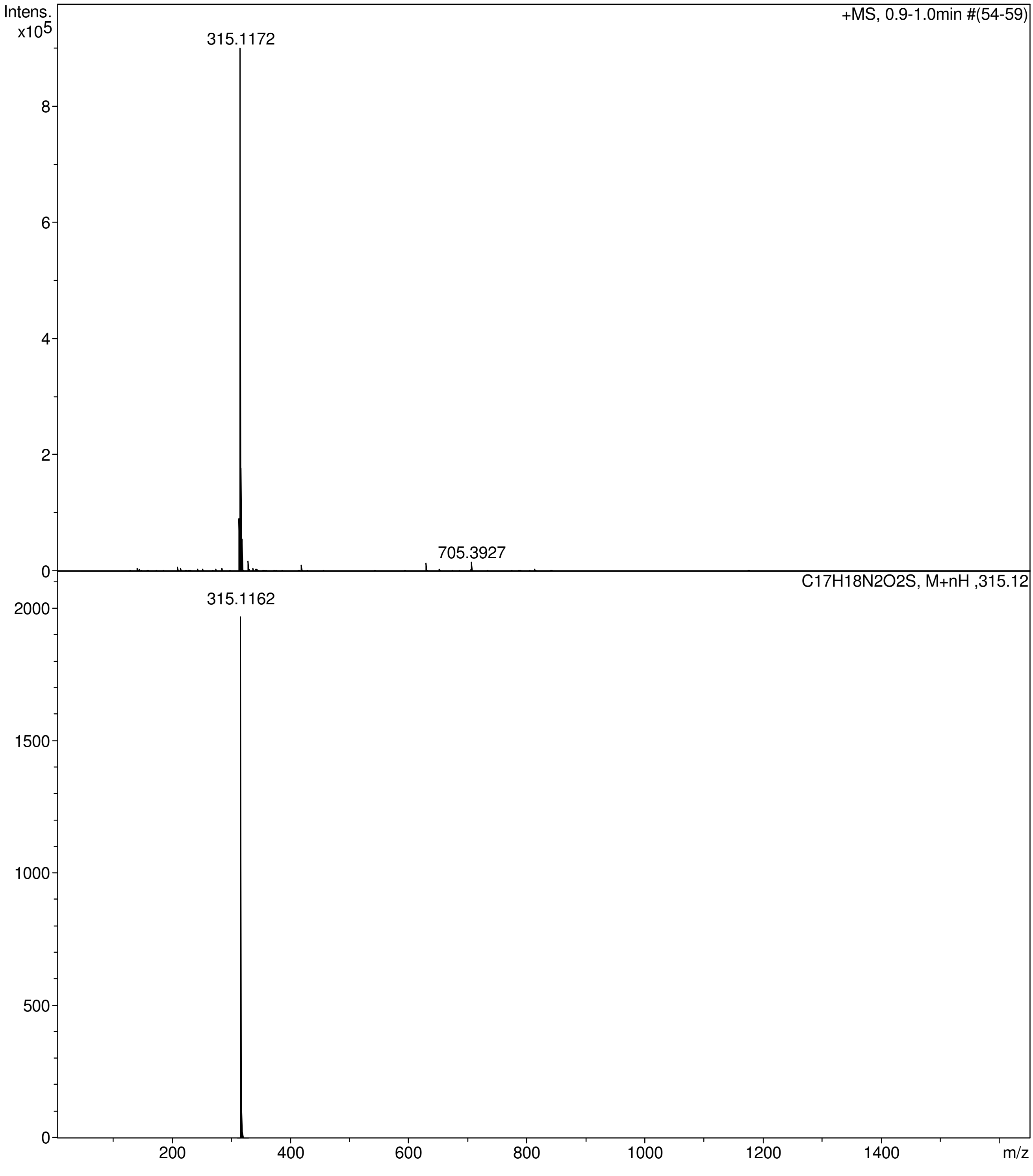

© Zelinsky Institute of Organic Chemistry, Moscow; Bruker AV300 SF=300.13 MHz {1H} SI=64K SW=6010 O1=2101 PW=15.0 AQ=2.715 RD=0.10 NS=1 SR=0.00 TE=300K 20 February 2021 Opr: NMR User; Solv: CDCl3;

The Best Applied NMR! On-Line: "http://nmr.ioc.ac.ru:8080"

/LPIK AF−402.2 Kokorekin−20259

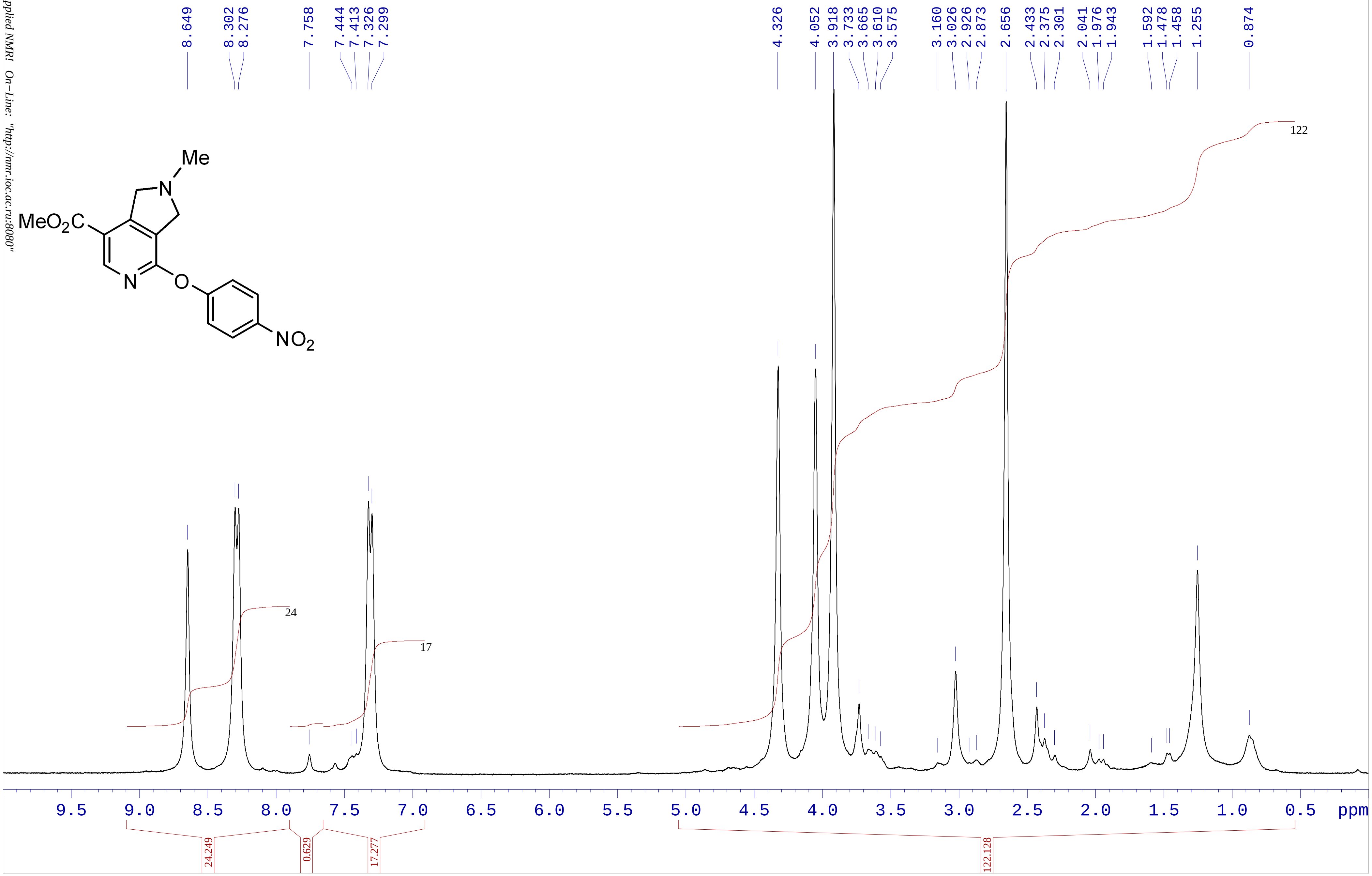

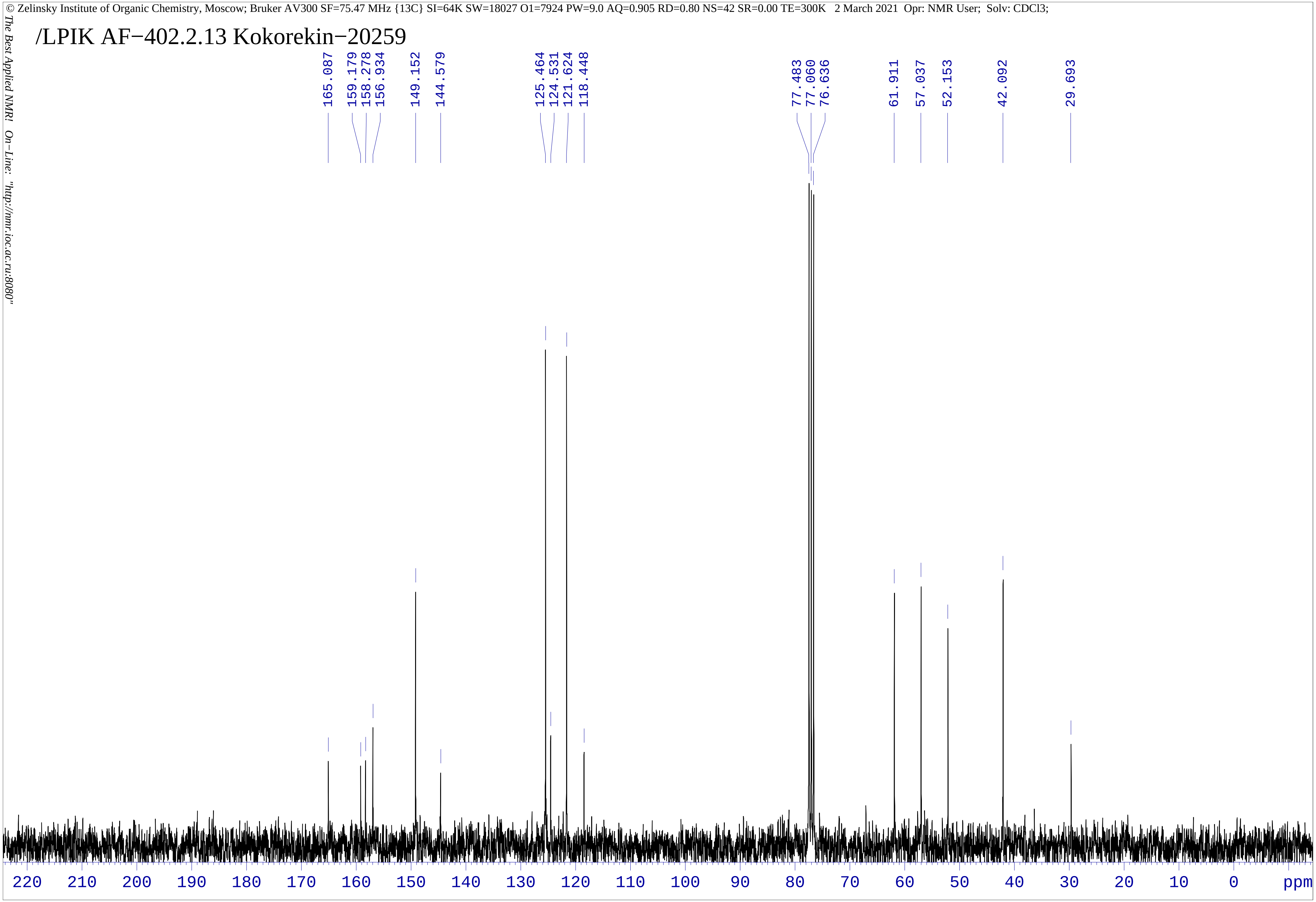
© Zelinsky Institute of Organic Chemistry, Moscow; Bruker AV300 SF=75.47 MHz {13C} SI=64K SW=18027 O1=7924 PW=9.0 AQ=0.905 RD=0.80 NS=42 SR=0.00 TE=300K 2 March 2021 Opr: NMR User; Solv: CDCl3;
The Best Applied NMR! On−Line: "http://nmr.ioc.ac.ru:8080"
/LPIK AF−402.2.13 Kokorekin−20259
165.087
159.179
158.278
156.934
149.152
144.579
125.464
124.531
121.624
118.448
77.483
77.060
76.636
61.911
57.037
52.153
42.092
29.693
220 210 200 190 180 170 160 150 140 130 120 110 100 90 80 70 60 50 40 30 20 10 0 ppm

Display Report

Analysis Info

Analysis Name	D:\Data\Kolotyrkina\2021\Bastrakov\0428029.d	Acquisition Date	28.04.2021 13:21:03
Method	tune_50-1600.m	Operator	BDAL@DE
Sample Name	/LPIK AF-402	Instrument / Ser#	micrOTOF 10248
Comment	C16H15N3O5 mH 330.1084 clb added CH3OH		

Acquisition Parameter

Source Type	ESI	Ion Polarity	Positive	Set Nebulizer	1.0 Bar
Focus	Not active			Set Dry Heater	200 °C
Scan Begin	50 m/z	Set Capillary	4500 V	Set Dry Gas	4.0 l/min
Scan End	1600 m/z	Set End Plate Offset	-500 V	Set Divert Valve	Waste

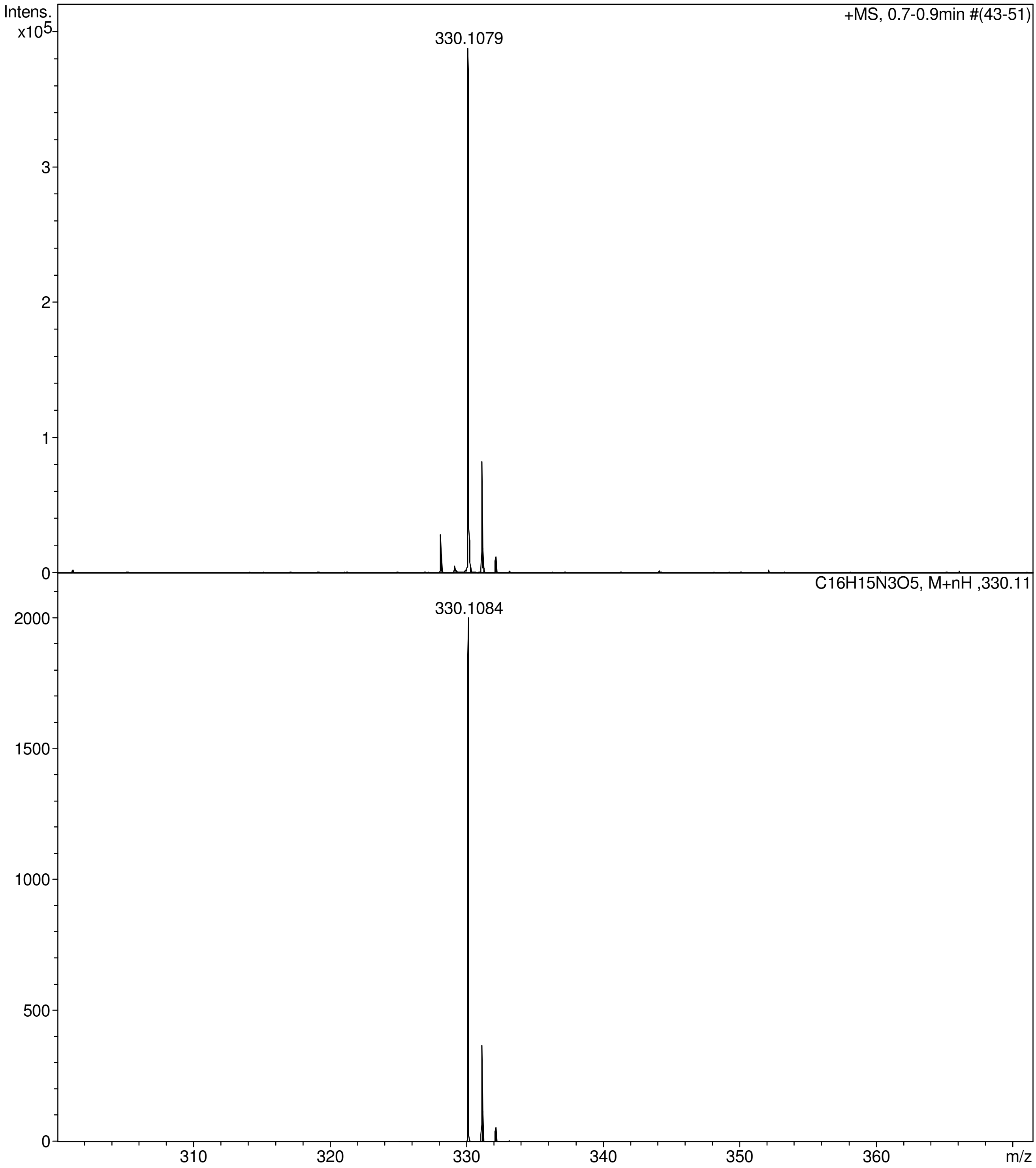

© Zelinsky Institute of Organic Chemistry, Moscow; Bruker AM300 SF=300.13 MHz {1H} SI=16K SW=6010 O1=2401 PW=9.0 AQ=1.352 RD=0.00 NS=1 SR=4.04 TE=300K 24 November 2020 Opr: Daeva E.D.; Solv: CDCl3;

The Best Applied NMR! On−Line: "http://nmr.ioc.ac.ru:8080"

/LPIK AF−393.2

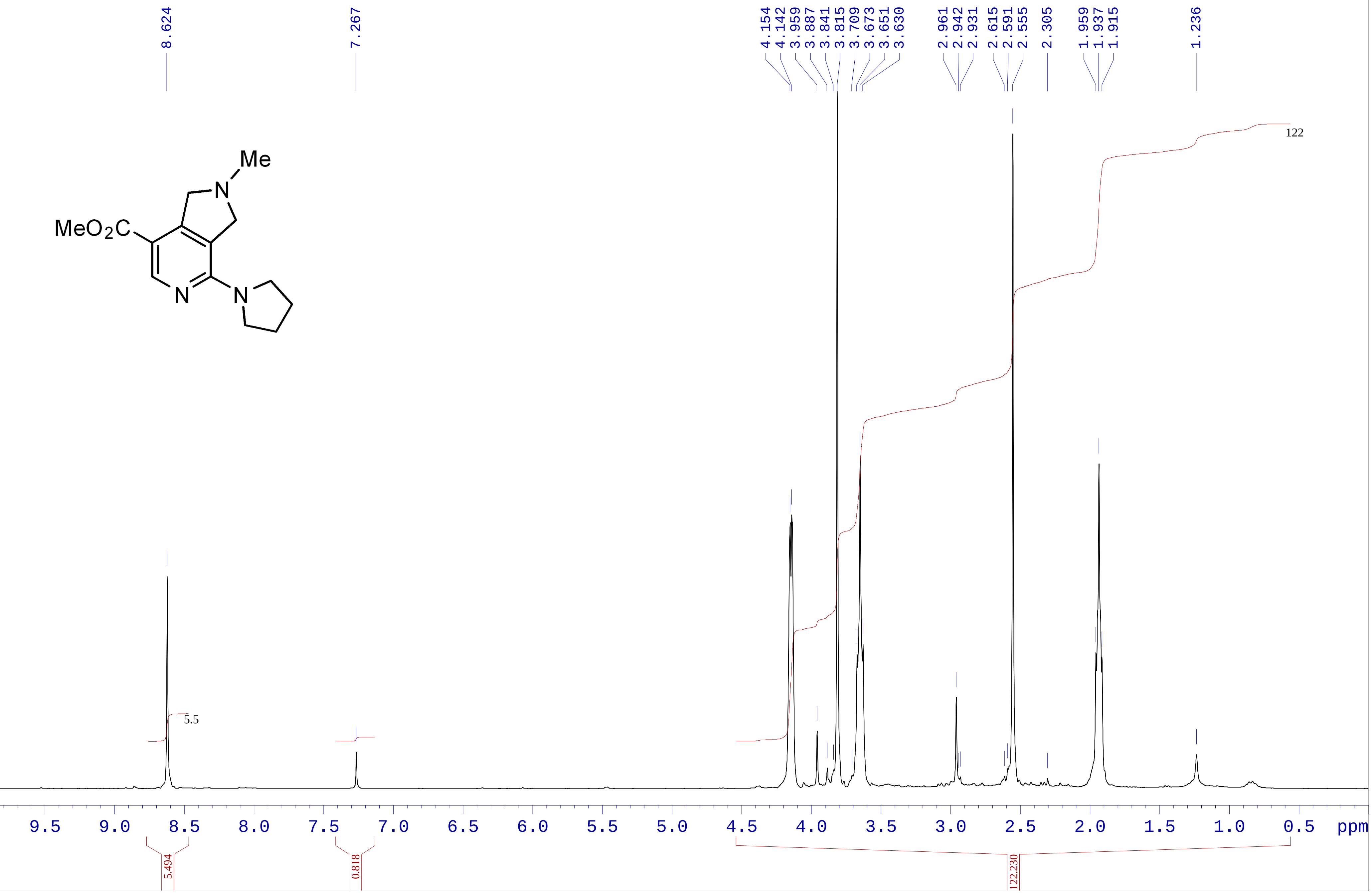

© Zelinsky Institute of Organic Chemistry, Moscow; Bruker AM300 SF=75.47 MHz {13C} SI=32K SW=18114 O1=8301 PW=13.0 AQ=0.900 RD=1.00 NS=588 SR=−3.88 TE=295K 26 November 2020 Opr: Daeva E.D.; Solv: CDCl3;

The Best Applied NMR! On−Line: "http://nmr.ioc.ac.ru:8080"

/LPIK AF−393.2.C13

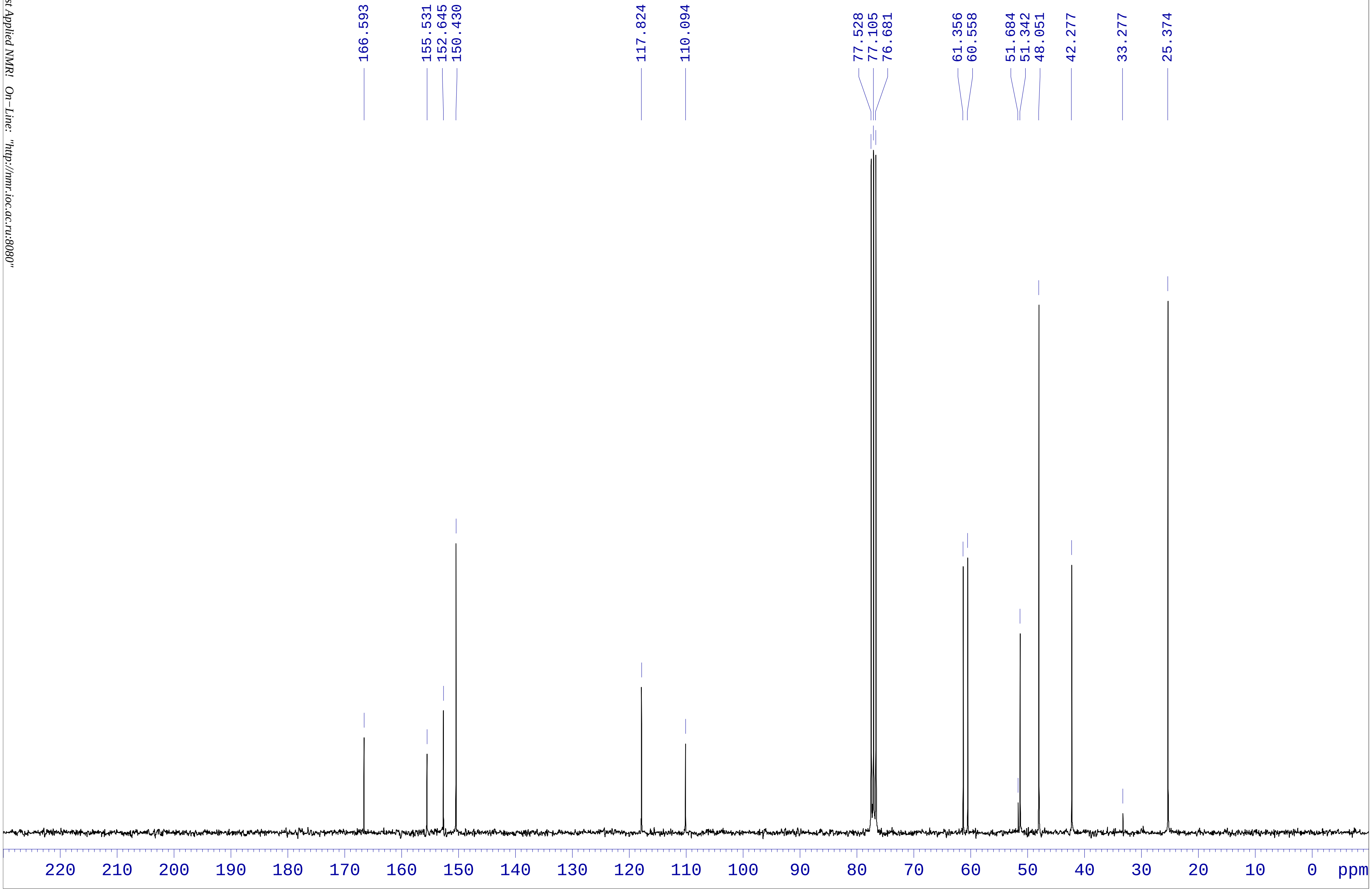

Display Report

Analysis Info

Analysis Name	D:\Data\Kolotyrkina\2021\Bastrakov\0211021.d	Acquisition Date	11.02.2021 12:48:04
Method	tune_50-1600.m	Operator	BDAL@DE
Sample Name	/LPIK AF-393.2	Instrument / Ser#	micrOTOF 10248
Comment	C14H19N3O2 mH 262.1550alibrant added CH3OH		

Acquisition Parameter

Source Type	ESI	Ion Polarity	Positive	Set Nebulizer	1.0 Bar
Focus	Not active			Set Dry Heater	200 °C
Scan Begin	50 m/z	Set Capillary	4500 V	Set Dry Gas	4.0 l/min
Scan End	1600 m/z	Set End Plate Offset	-500 V	Set Divert Valve	Waste

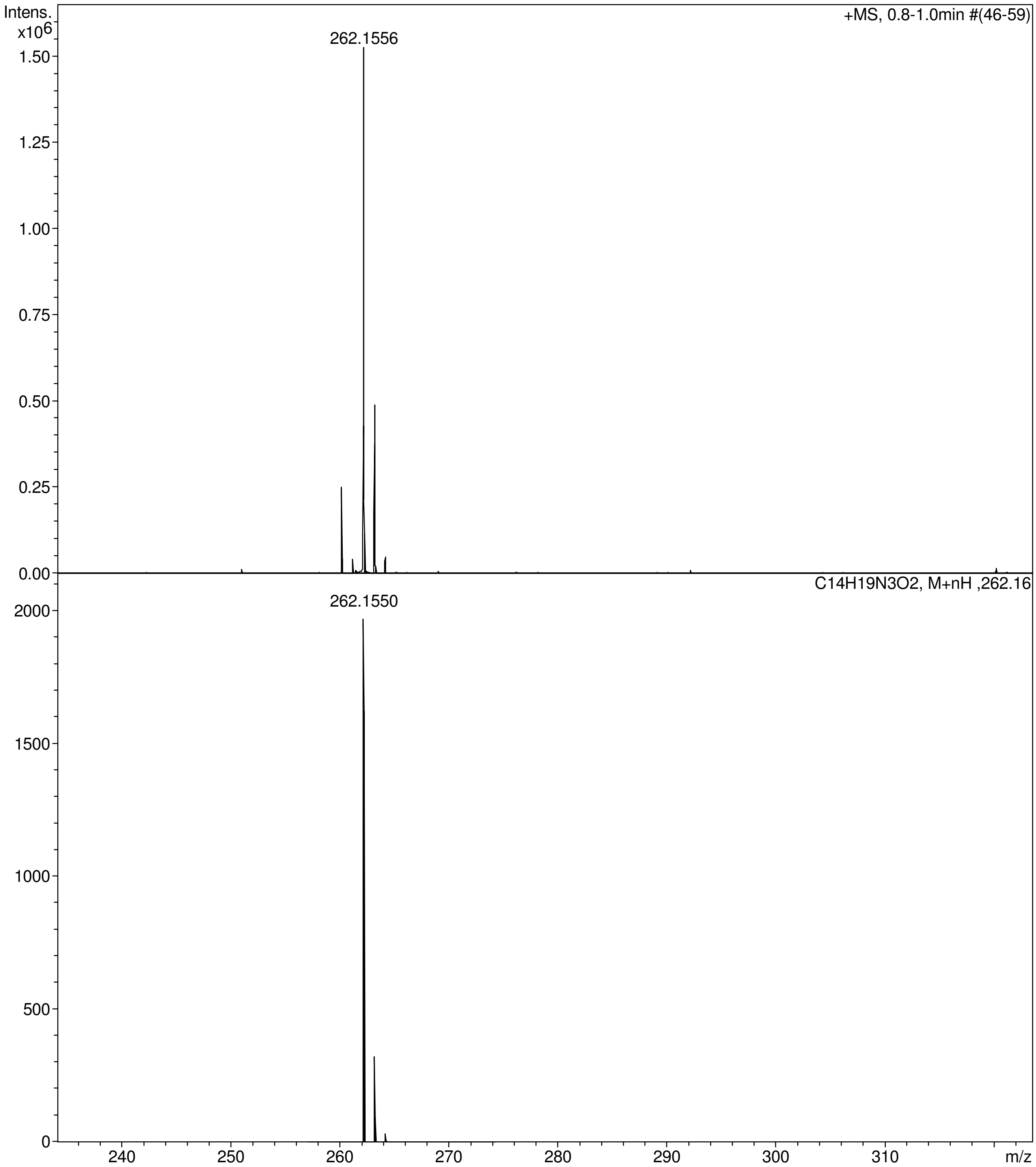

© Zelinsky Institute of Organic Chemistry, Moscow; Bruker AM300 SF=300.13 MHz {1H} SI=32K SW=6010 O1=2401 PW=9.0 AQ=1.352 RD=0.00 NS=1 SR=4.04 TE=300K 3 December 2020 Opr: Daeva E.D.; Solv: CDCl3;

The Best Applied NMR! On−Line: "http://nmr.ioc.ac.ru:8080"

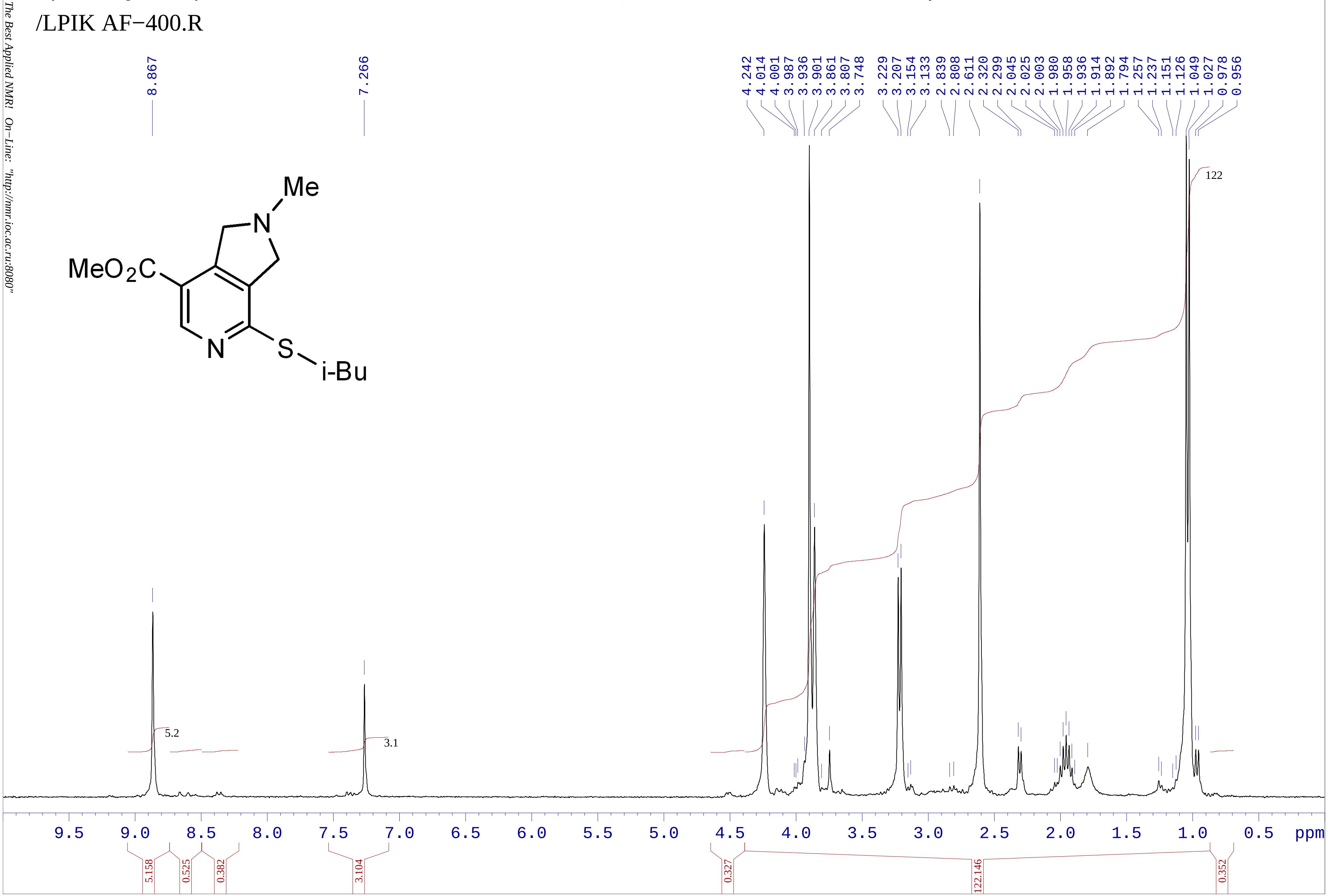

© Zelinsky Institute of Organic Chemistry, Moscow; Bruker AV300 SF=75.47 MHz {13C} SI=64K SW=18027 O1=7924 PW=9.0 AQ=0.905 RD=0.80 NS=142 SR=0.00 TE=300K 2 March 2021 Opr: NMR User; Solv: CDCl3;

The Best Applied NMR! On-Line: "http://nmr.ioc.ac.ru:8080"

/LPIK AF−400.1.13 Kokorekin−20259

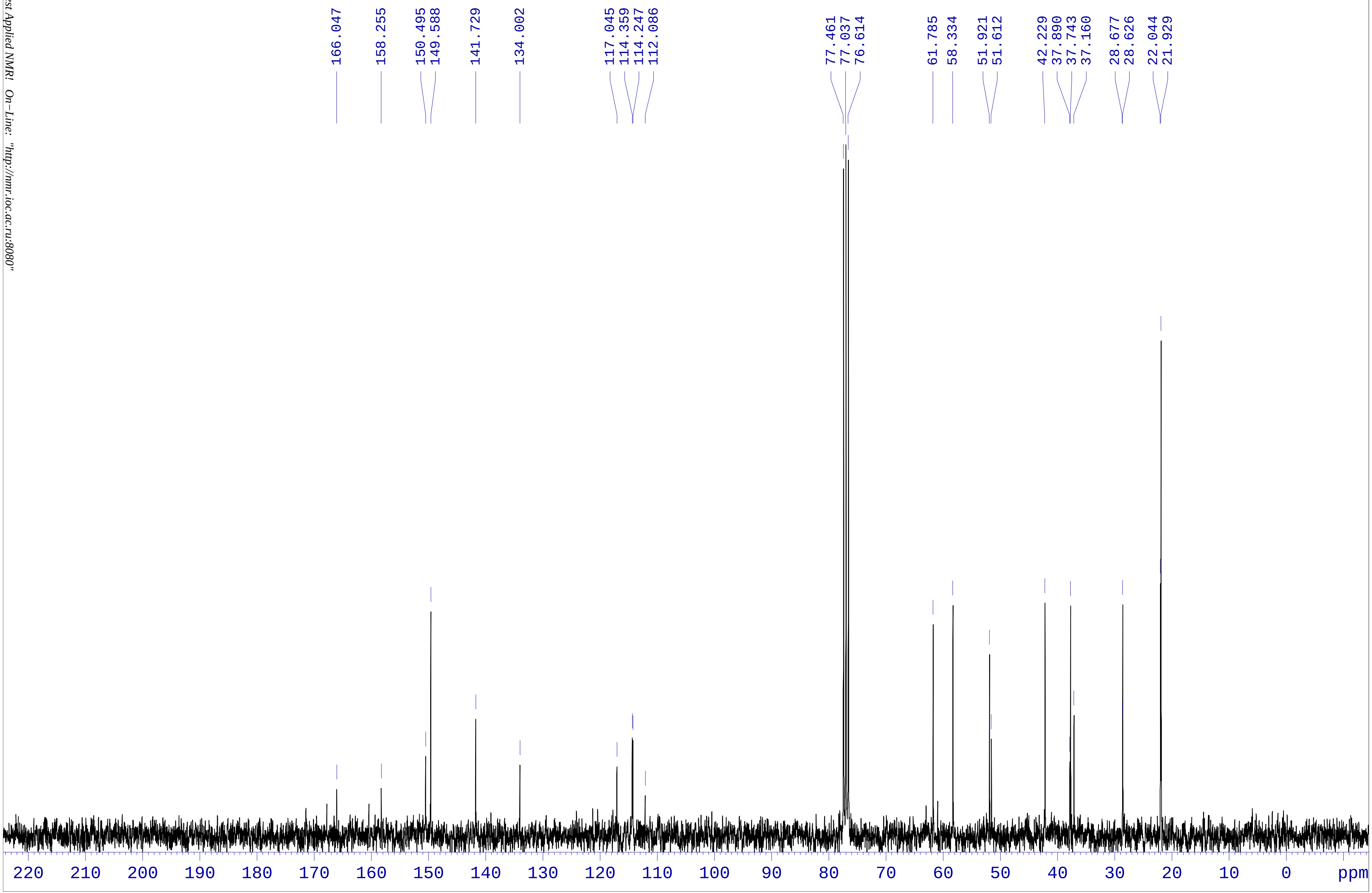

Display Report

Analysis Info

Analysis Name	D:\Data\Kolotyrkina\2021\Bastrakov\0211023.d	Acquisition Date	11.02.2021 13:03:54
Method	tune_50-1600.m	Operator	BDAL@DE
Sample Name	/LPIK AF-400	Instrument / Ser#	micrOTOF 10248
Comment	C14H20N2O2S mH 281.1318 calibrant added CH3OH		

Acquisition Parameter

Source Type	ESI	Ion Polarity	Positive	Set Nebulizer	1.0 Bar
Focus	Not active			Set Dry Heater	200 °C
Scan Begin	50 m/z	Set Capillary	4500 V	Set Dry Gas	4.0 l/min
Scan End	1600 m/z	Set End Plate Offset	-500 V	Set Divert Valve	Waste

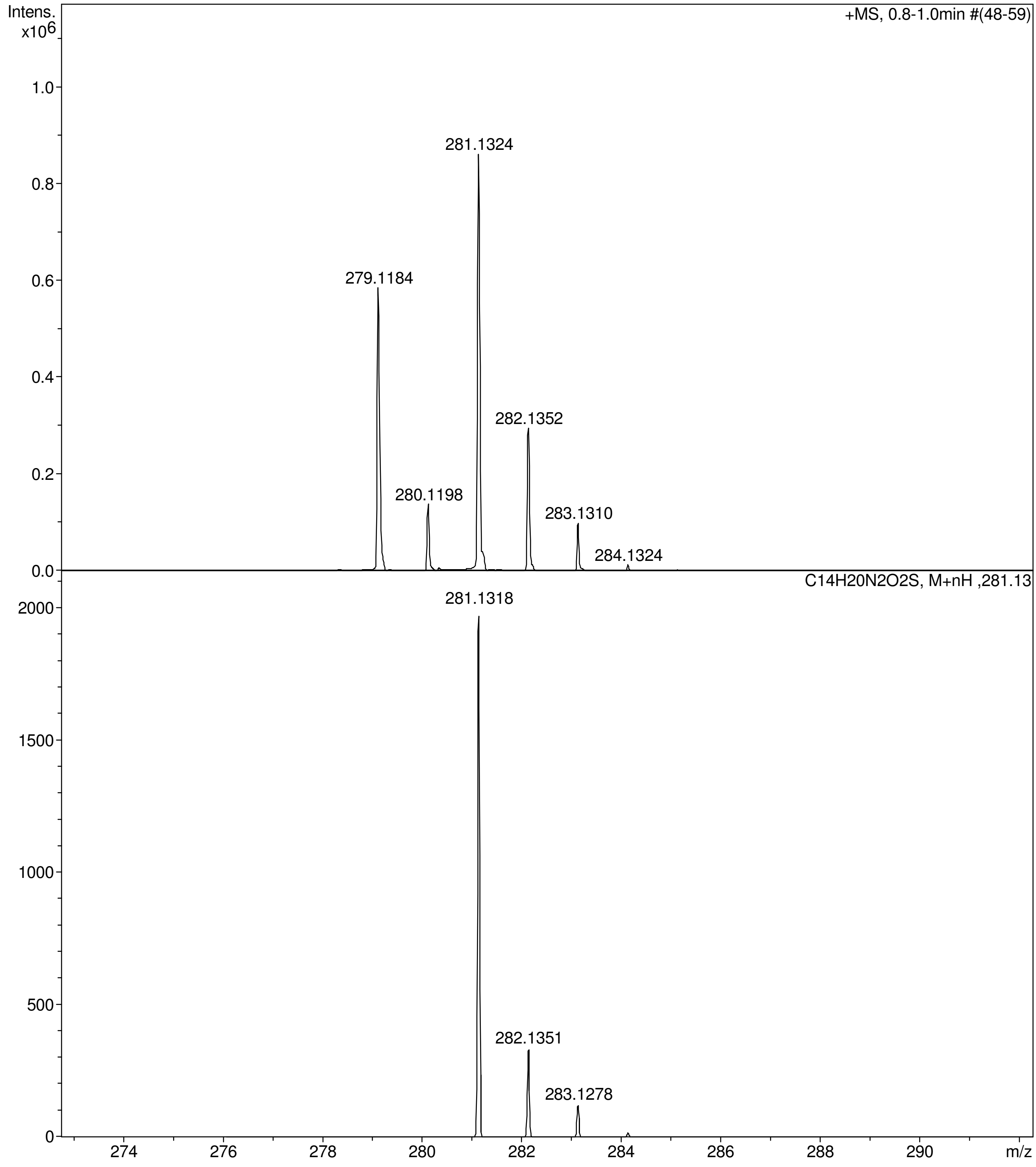

© Zelinsky Institute of Organic Chemistry, Moscow; Bruker AM300 SF=300.13 MHz {1H} SI=32K SW=6010 O1=2401 PW=9.0 AQ=1.352 RD=0.00 NS=1 SR=0.00 TE=300K 16 March 2021 Opr: Daeva E.D.; Solv: CDCl3;

The Best Applied NMR! On−Line: "http://nmr.ioc.ac.ru:8080"

/LPIK AF−409

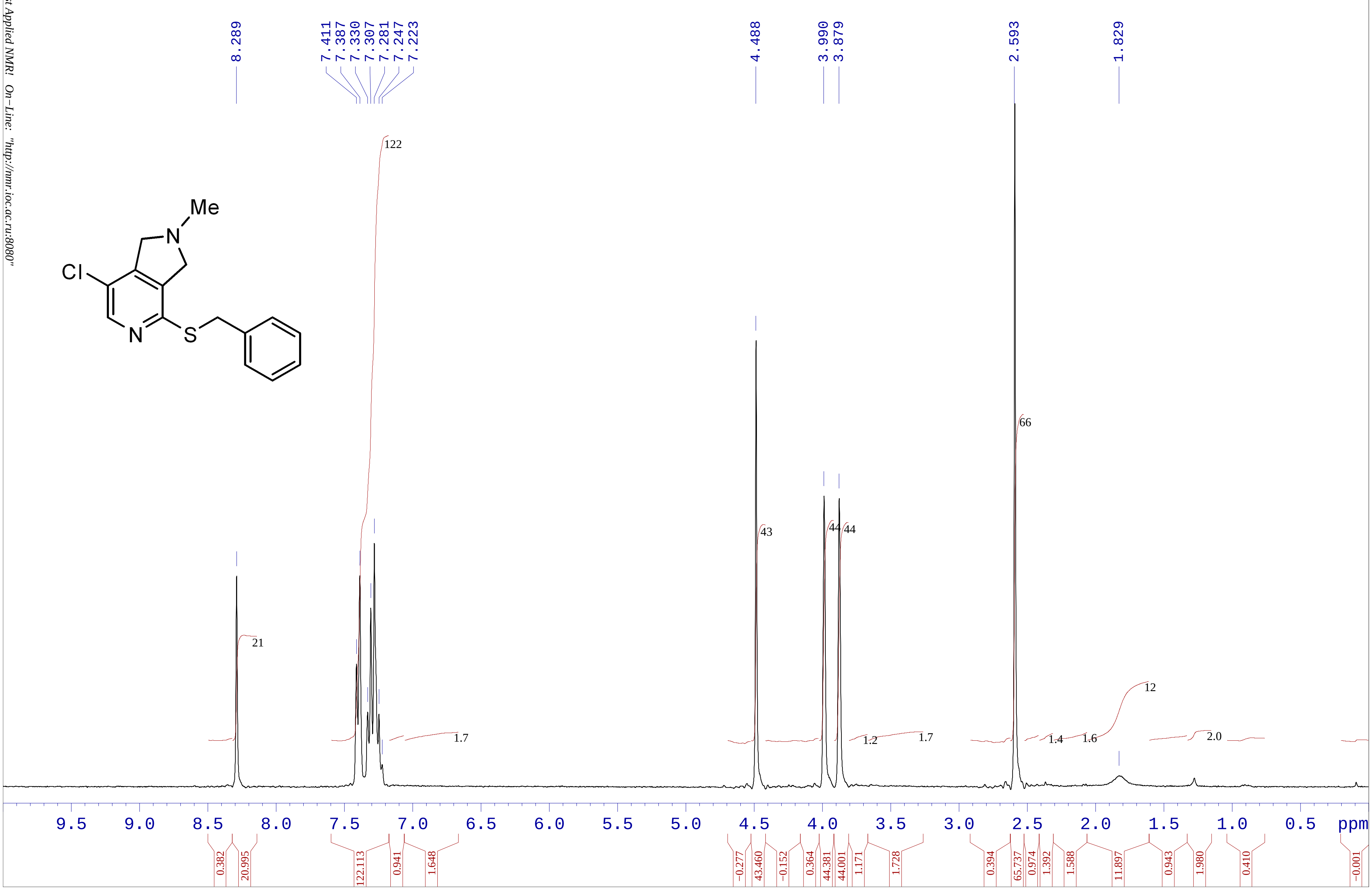

© Zelinsky Institute of Organic Chemistry, Moscow; Bruker DRX500 SF=125.76 MHz {13C} SI=32K SW=32676 O1=13844 PW=6.3 AQ=0.500 RD=2.00 NS=208 SR=11.12 TE=300K 18 March 2021 Opr: Strelenko Yu.A.; Solv: CDCl3;

The Best Applied NMR! On-Line: "http://nmr.ioc.ac.ru:8080"

/LPIK AF−409.C13

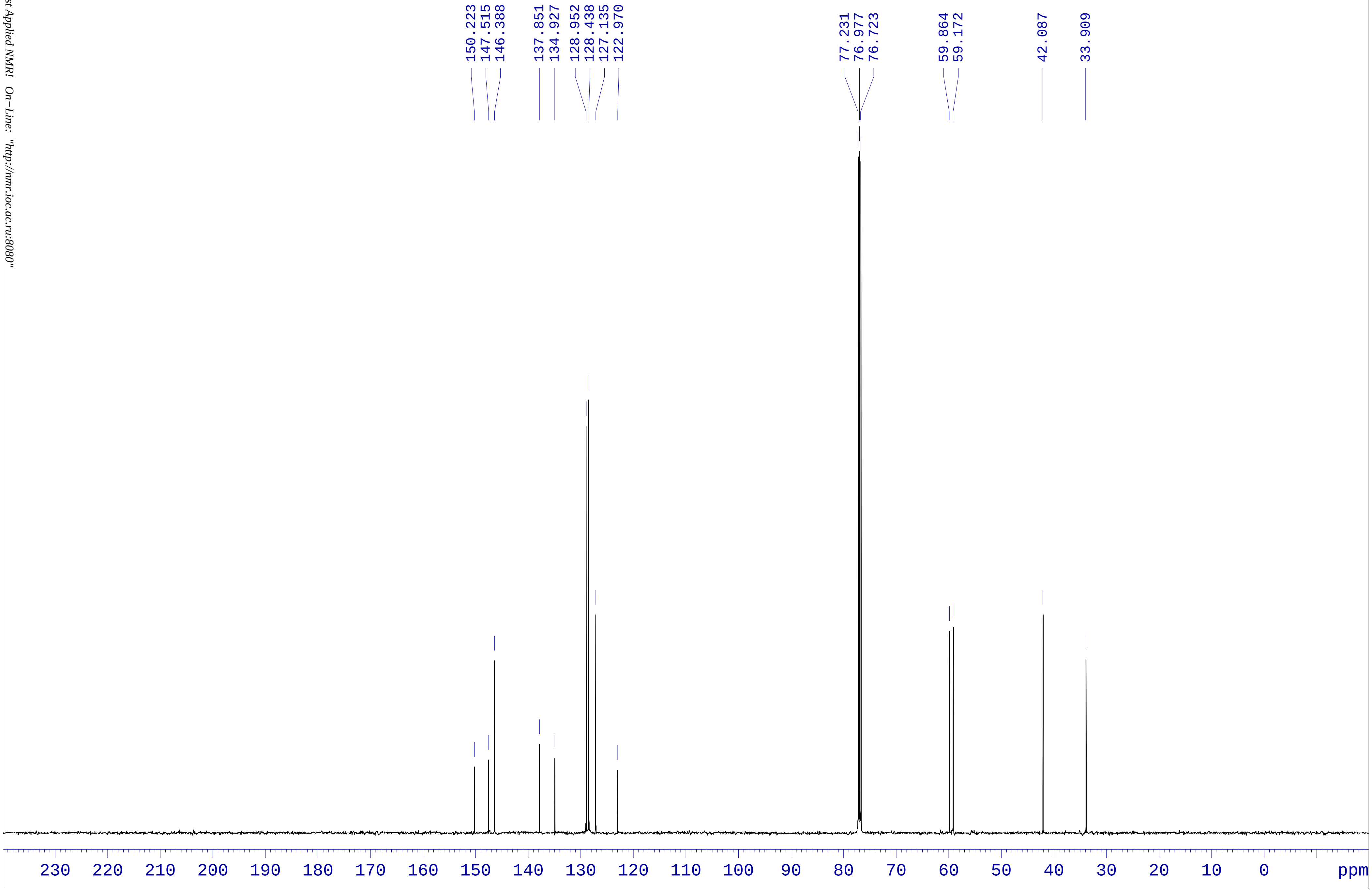

Display Report

Analysis Info

Analysis Name	D:\Data\Kolotyrkina\2021\Bastrakov\0428030.d	Acquisition Date	28.04.2021 13:26:23
Method	tune_50-1600.m	Operator	BDAL@DE
Sample Name	/LPIK AF-409	Instrument / Ser#	micrOTOF 10248
Comment	C15H15ClN2S mH 291.0717 clb added CH3OH		

Acquisition Parameter

Source Type	ESI	Ion Polarity	Positive	Set Nebulizer	1.0 Bar
Focus	Not active			Set Dry Heater	200 °C
Scan Begin	50 m/z	Set Capillary	4500 V	Set Dry Gas	4.0 l/min
Scan End	1600 m/z	Set End Plate Offset	-500 V	Set Divert Valve	Waste

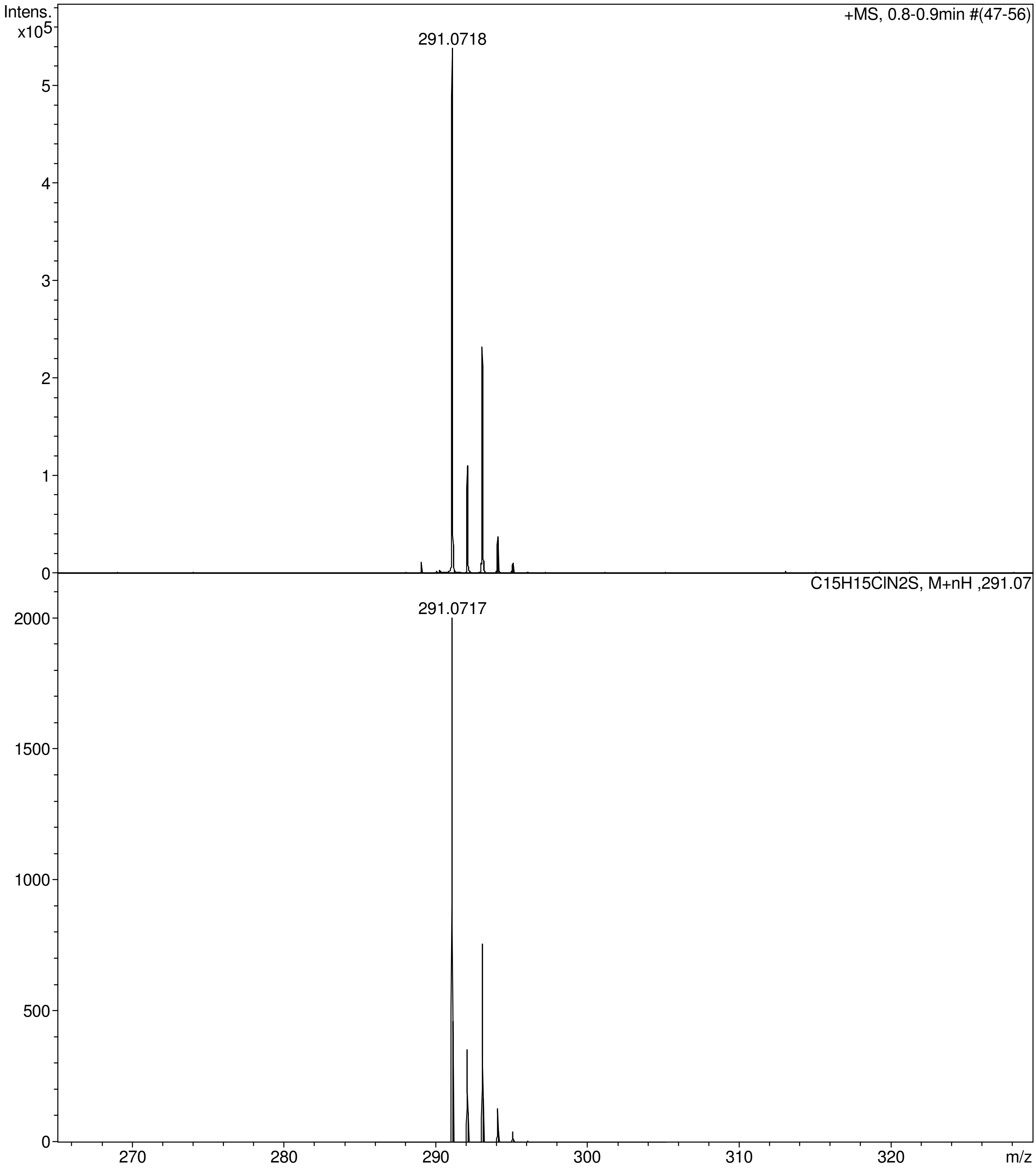

© Zelinsky Institute of Organic Chemistry, Moscow; Bruker AM300 SF=300.13 MHz {1H} SI=32K SW=6010 O1=2401 PW=9.0 AQ=1.352 RD=0.00 NS=1 SR=0.00 TE=299K 17 March 2021 Opr: Daeva E.D.; Solv: CDCl3;

The Best Applied NMR! On−Line: "http://nmr.ioc.ac.ru:8080"

/LPIK AF−413

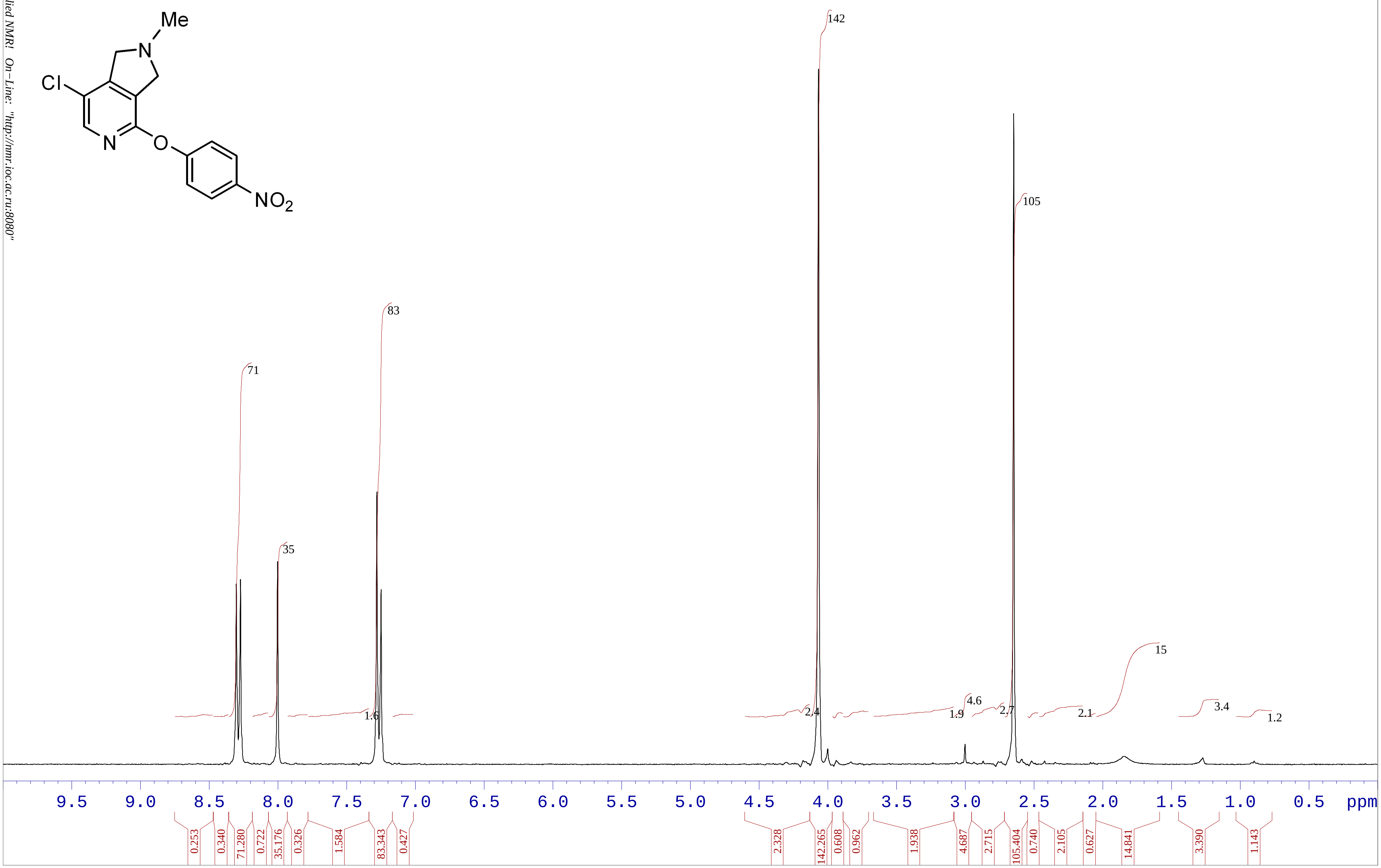

© Zelinsky Institute of Organic Chemistry, Moscow; Bruker DRX500 SF=125.76 MHz {13C} SI=32K SW=32676 O1=13844 PW=6.3 AQ=0.500 RD=2.00 NS=41 SR=11.12 TE=300K 18 March 2021 Opr: Strelenko Yu.A.; Solv: CDCl3;

The Best Applied NMR! On−Line: "http://nmr.ioc.ac.ru:8080"

/LPIK AF−413.C13

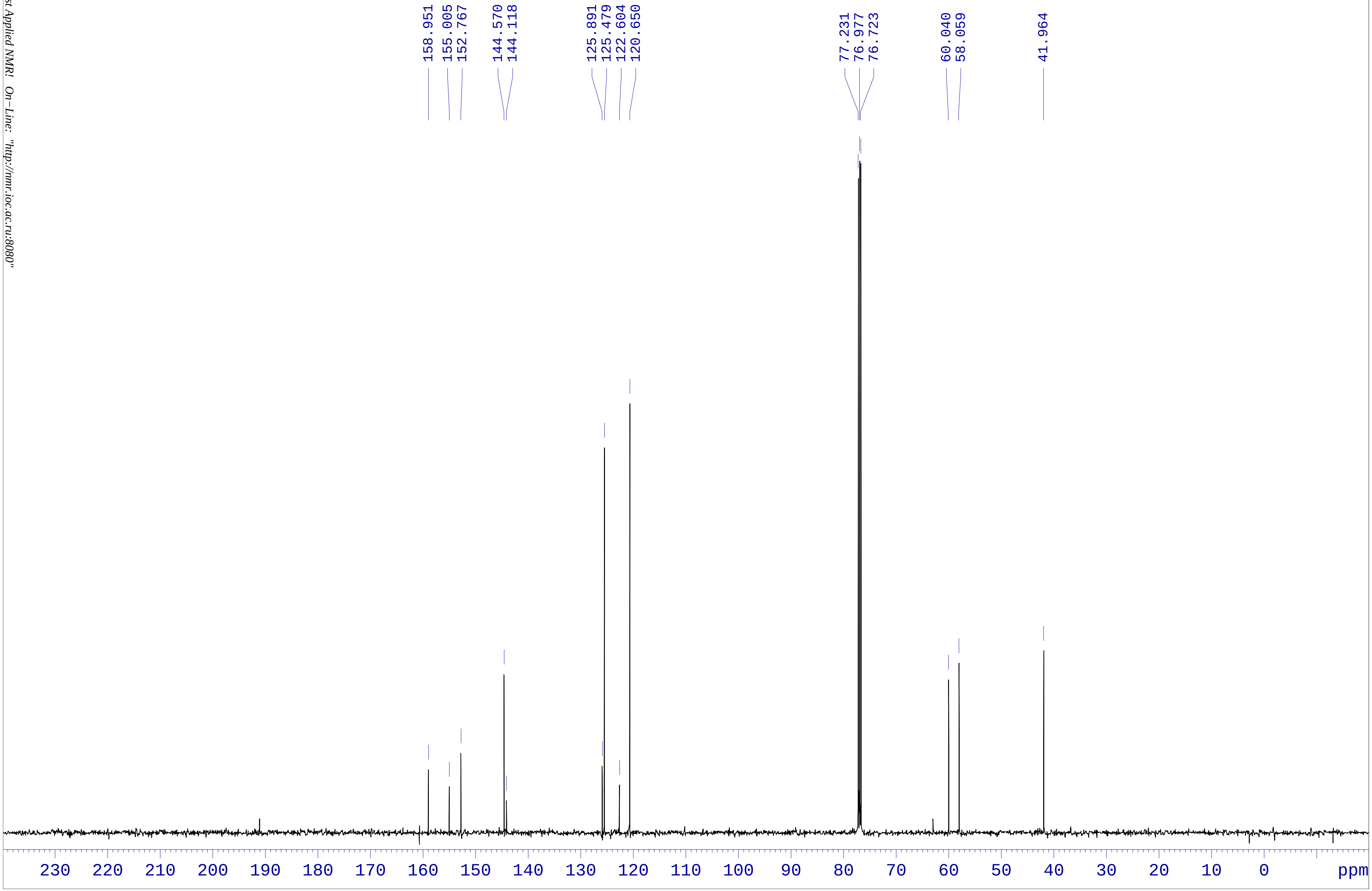

Display Report

Analysis Info

Analysis Name	D:\Data\Kolotyrkina\2021\Bastrakov\0429049.d
Method	tune_50-1600.m
Sample Name	/LPIK AF-413
Comment	C14H12ClN3O3 mH 306.0639 clb added CH3OH
Acquisition Date	29.04.2021 17:57:27
Operator	BDAL@DE
Instrument / Ser#	micrOTOF 10248

Acquisition Parameter

Source Type	ESI	Ion Polarity	Positive	Set Nebulizer	1.0 Bar
Focus	Not active			Set Dry Heater	200 °C
Scan Begin	50 m/z	Set Capillary	4500 V	Set Dry Gas	4.0 l/min
Scan End	1600 m/z	Set End Plate Offset	-500 V	Set Divert Valve	Waste

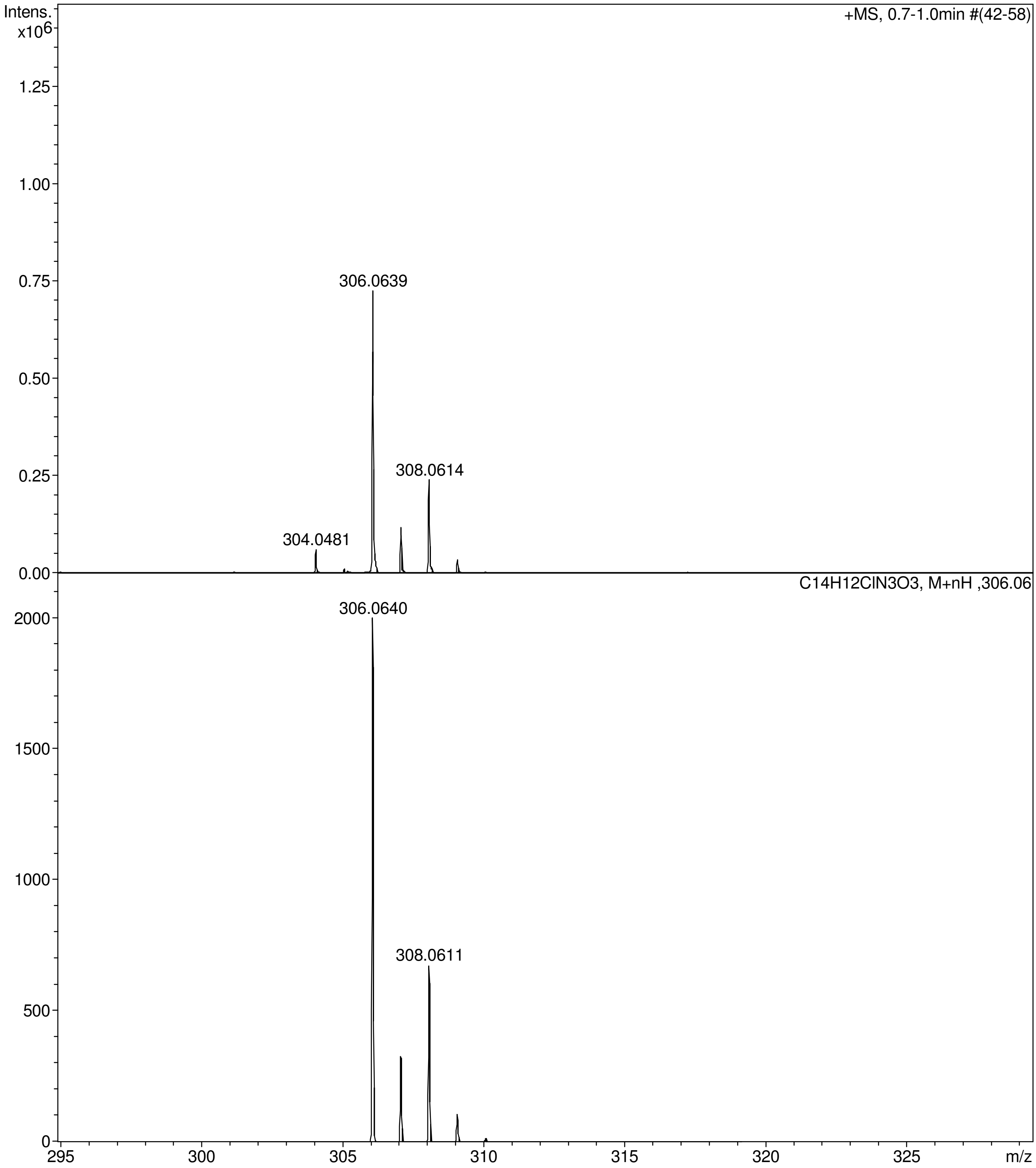

© Zelinsky Institute of Organic Chemistry, Moscow; Bruker AM300 SF=300.13 MHz {1H} SI=16K SW=6010 O1=2401 PW=9.0 AQ=1.351 RD=1.00 NS=1 SR=24.66 TE=297K 5 April 2021 Opr: Shulishov Eu.V.; Solv: CDCl3;

The Best Applied NMR! On-Line: "http://nmr.ioc.ac.ru:8080"

/LPIK AF−429

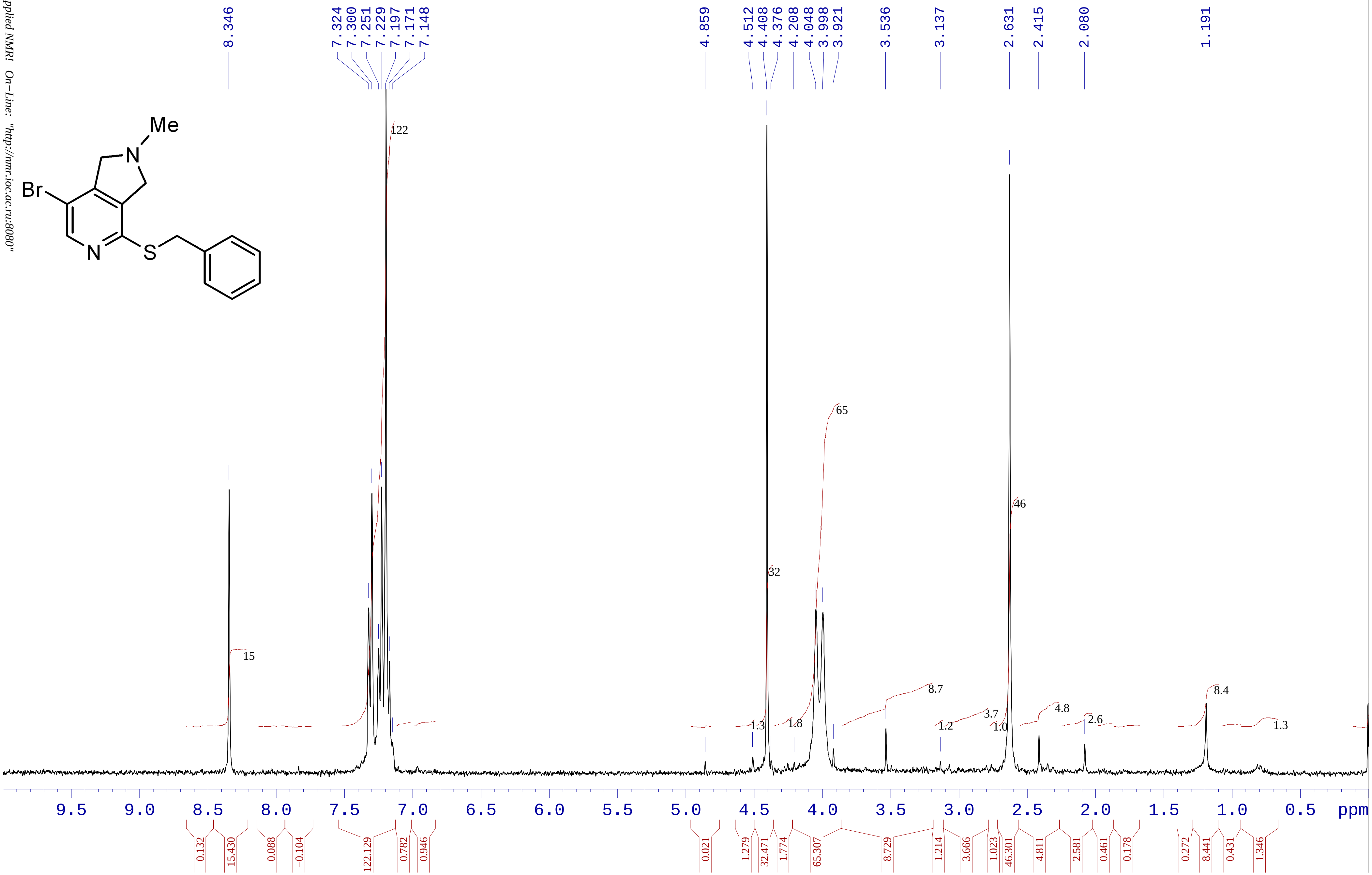

© Zelinsky Institute of Organic Chemistry, Moscow; Bruker AV300 SF=75.47 MHz {13C} SI=64K SW=18027 O1=7924 PW=9.0 AQ=0.905 RD=0.80 NS=97 SR=0.00 TE=300K 8 April 2021 Opr: NMR User; Solv: CDCl3;

The Best Applied NMR! On−Line: "http://nmr.ioc.ac.ru:8080"

/LPIK AF−429.1.13 Kokorekin−20259

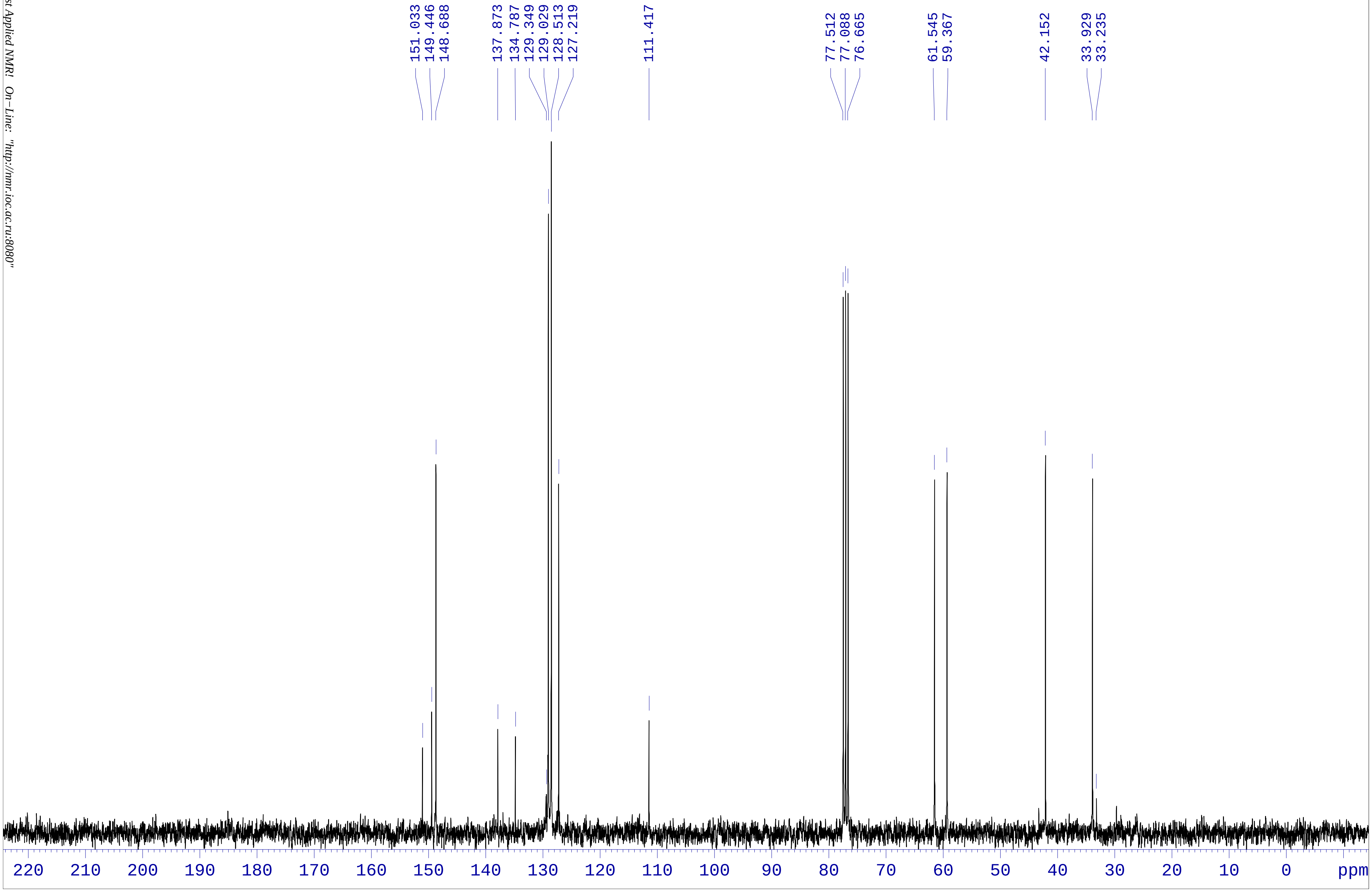

Display Report

Analysis Info

Analysis Name	D:\Data\Kolotyrkina\2021\Bastrakov\0707026.d	Acquisition Date	07.07.2021 16:12:07
Method	tune_50-1600.m	Operator	BDAL@DE
Sample Name	/LPIK AF-429P	Instrument / Ser#	micrOTOF 10248
Comment	C15H15BrN2S mH 335.0212 calibrant added, CH3OH		

Acquisition Parameter

Source Type	ESI	Ion Polarity	Positive	Set Nebulizer	1.0 Bar
Focus	Not active			Set Dry Heater	200 °C
Scan Begin	50 m/z	Set Capillary	4500 V	Set Dry Gas	4.0 l/min
Scan End	1600 m/z	Set End Plate Offset	-500 V	Set Divert Valve	Waste

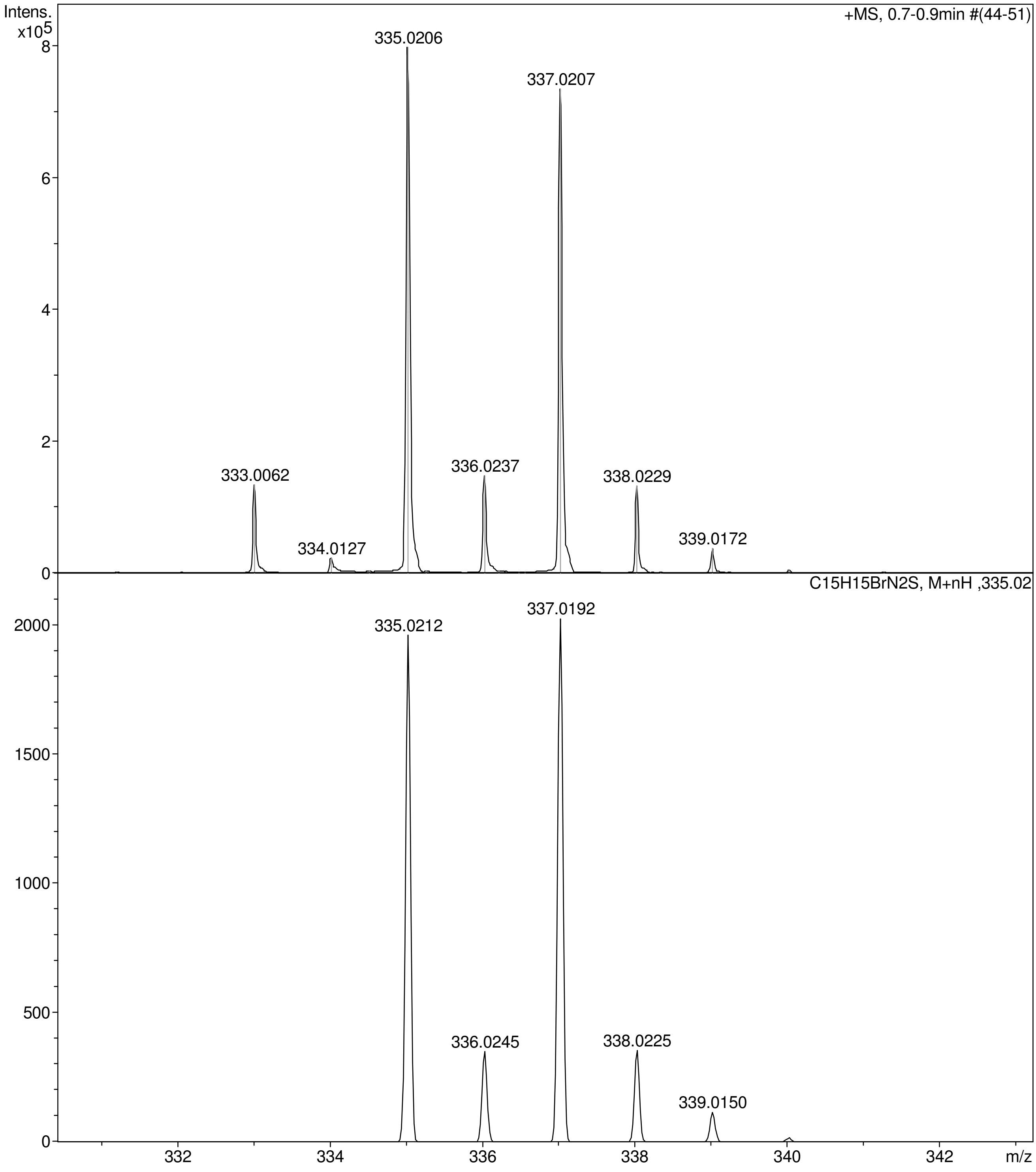

© Zelinsky Institute of Organic Chemistry, Moscow; Bruker AM300 SF=300.13 MHz {1H} SI=16K SW=6010 O1=2401 PW=9.0 AQ=1.351 RD=1.00 NS=1 SR=3.53 TE=298K 7 April 2021 Opr: Shulishov Eu.V.; Solv: CDCl3;

The Best Applied NMR! On-Line: "http://nmr.ioc.ac.ru:8080"

/LPIK AF−430

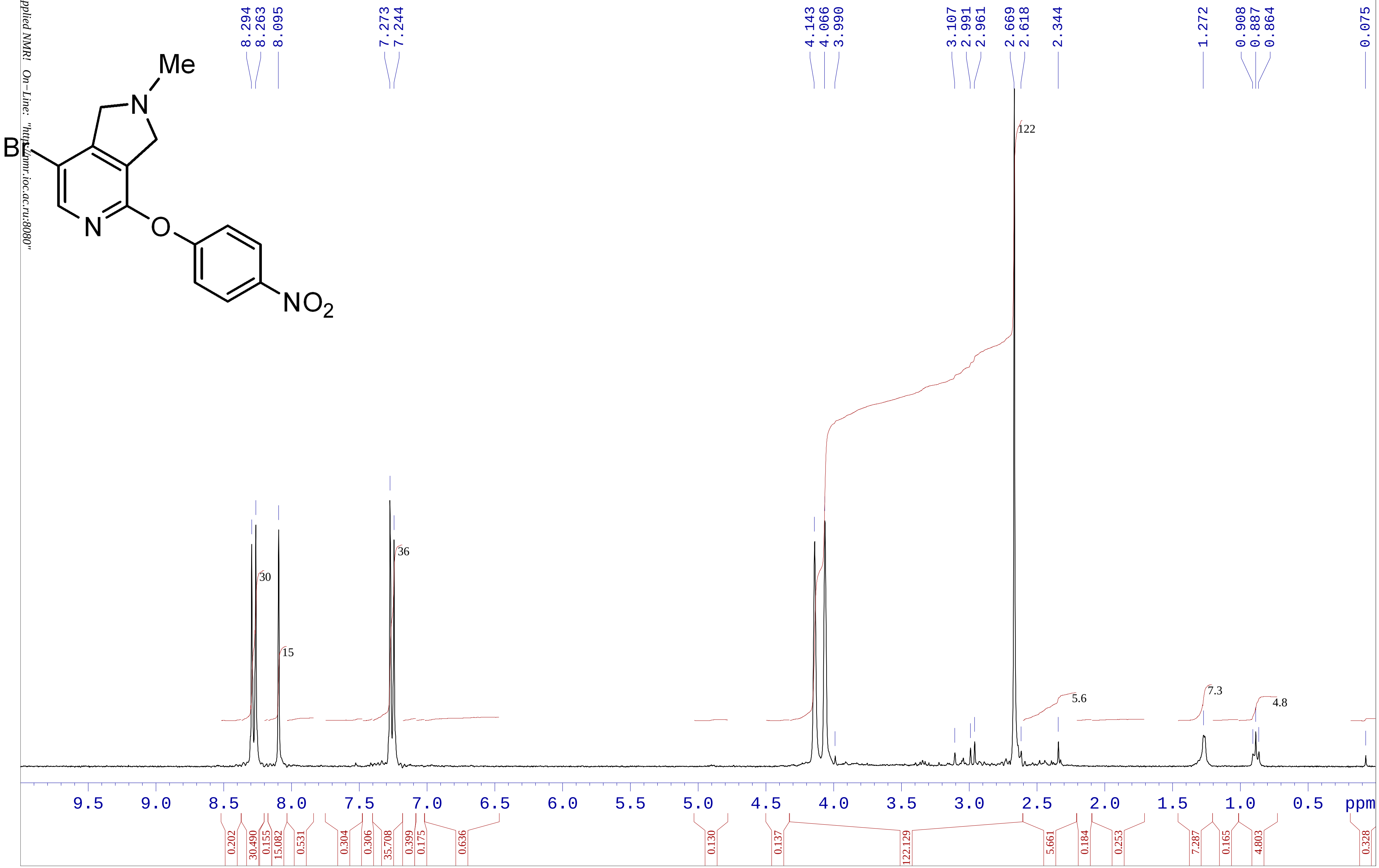

© Zelinsky Institute of Organic Chemistry, Moscow; Bruker AM300 SF=75.47 MHz {13C} SI=32K SW=18114 O1=8301 PW=14.3 AQ=0.901 RD=0.30 NS=1929 SR=−4.08 TE=297K 7 April 2021 Opr: Shulishov Eu.V.; Solv: CDCl3;

The Best Applied NMR! On−Line: "http://nmr.ioc.ac.ru:8080"

/LPIK AF−430.C13

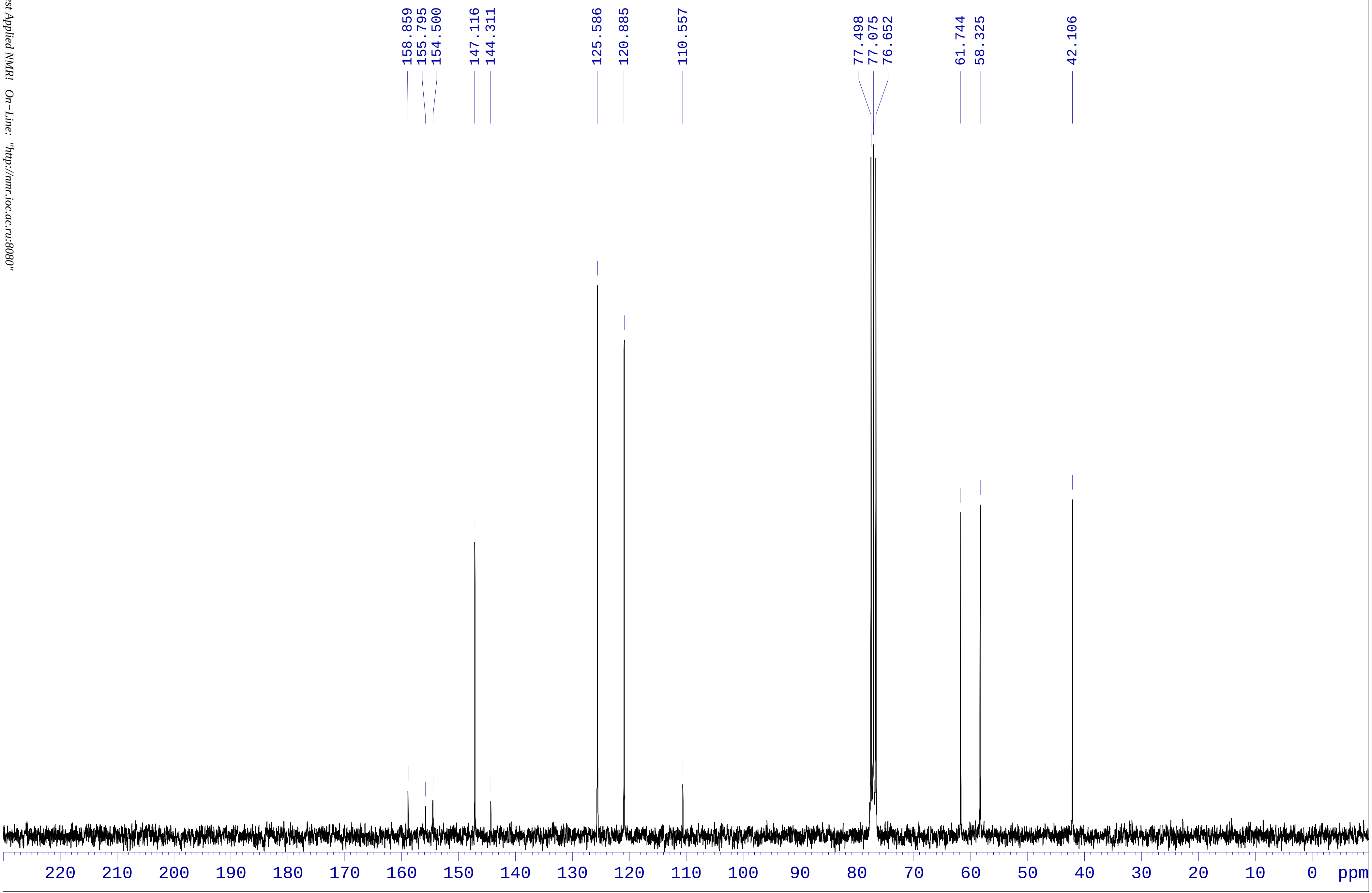

Display Report

Analysis Info

Analysis Name	D:\Data\Kolotyrkina\2021\Bastrakov\0429051.d	Acquisition Date	29.04.2021 18:06:30
Method	tune_50-1600.m	Operator	BDAL@DE
Sample Name	/LPIK AF-430	Instrument / Ser#	micrOTOF 10248
Comment	C14H12BrN3O3 mH 350.0134clb added CH3OH		

Acquisition Parameter

Source Type	ESI	Ion Polarity	Positive	Set Nebulizer	1.0 Bar
Focus	Not active			Set Dry Heater	200 °C
Scan Begin	50 m/z	Set Capillary	4500 V	Set Dry Gas	4.0 l/min
Scan End	1600 m/z	Set End Plate Offset	-500 V	Set Divert Valve	Waste

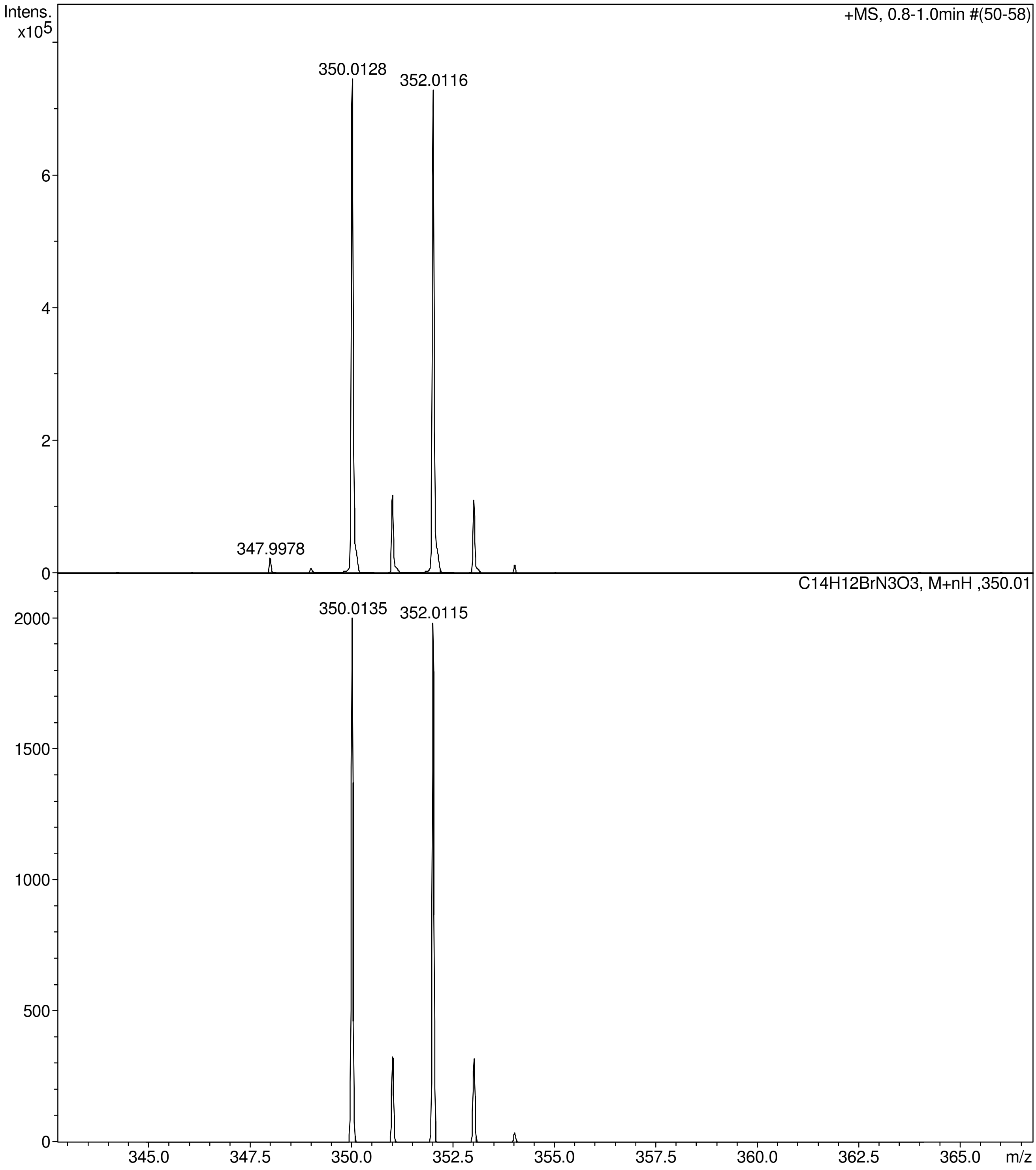

© Zelinsky Institute of Organic Chemistry, Moscow; Bruker AV300 SF=300.13 MHz {1H} SI=64K SW=6010 O1=2101 PW=15.0 AQ=2.715 RD=0.10 NS=1 SR=0.00 TE=300K 17 May 2021 Opr: NMR User; Solv: CDCl3;

The Best Applied NMR! On-Line: "http://nmr.ioc.ac.ru:8080"

/LPIK AF−432.1.1 Kokorekin−20259

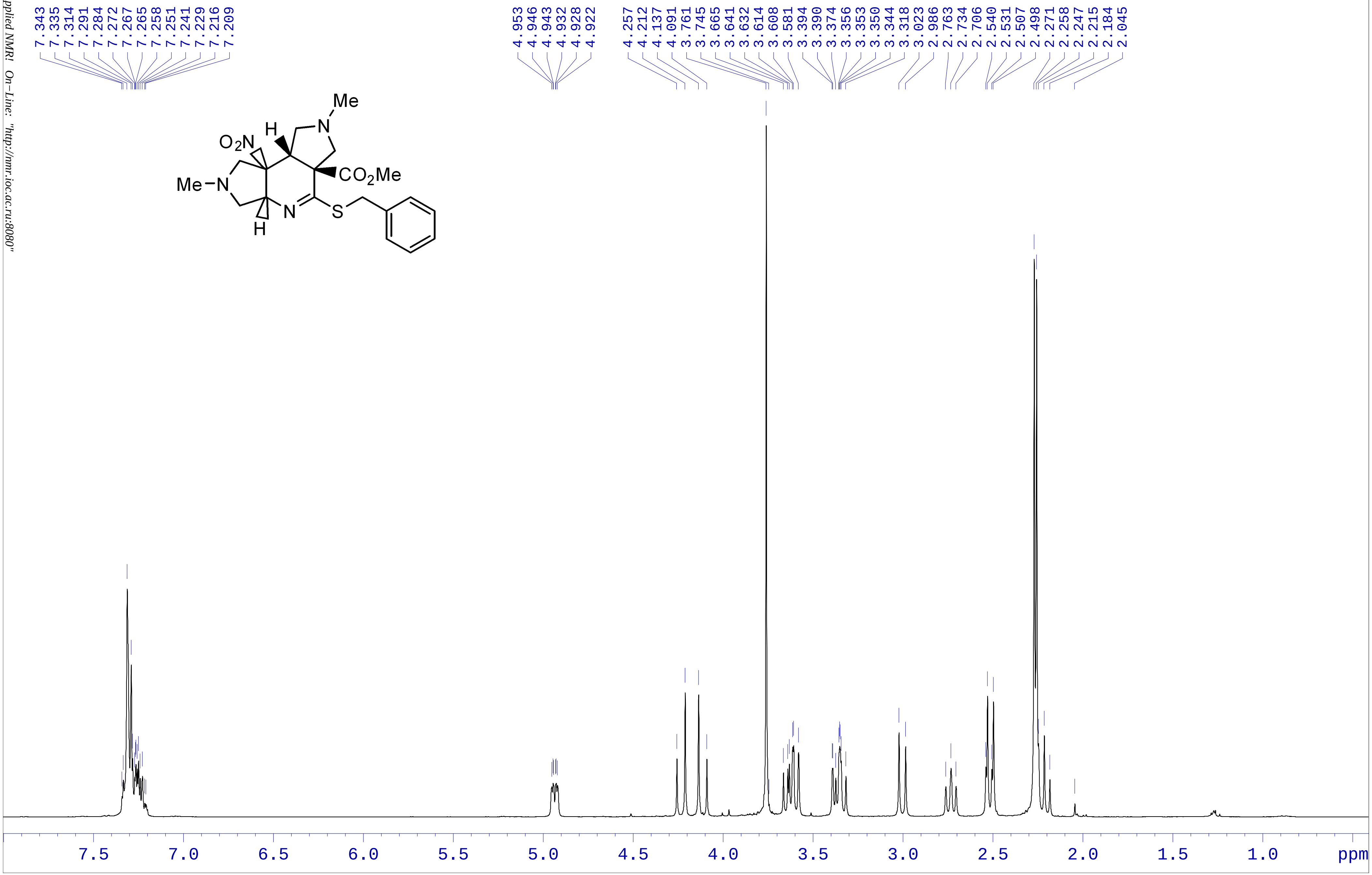

© Zelinsky Institute of Organic Chemistry, Moscow; Bruker AV300 SF=75.47 MHz {13C} SI=64K SW=18027 O1=7924 PW=9.0 AQ=0.905 RD=0.80 NS=103 SR=0.00 TE=300K 16 April 2021 Opr: NMR User; Solv: CDCl3;

The Best Applied NMR! On−Line: "http://nmr.ioc.ac.ru:8080"

/LPIK AF−432.1 Kokorekin−20259

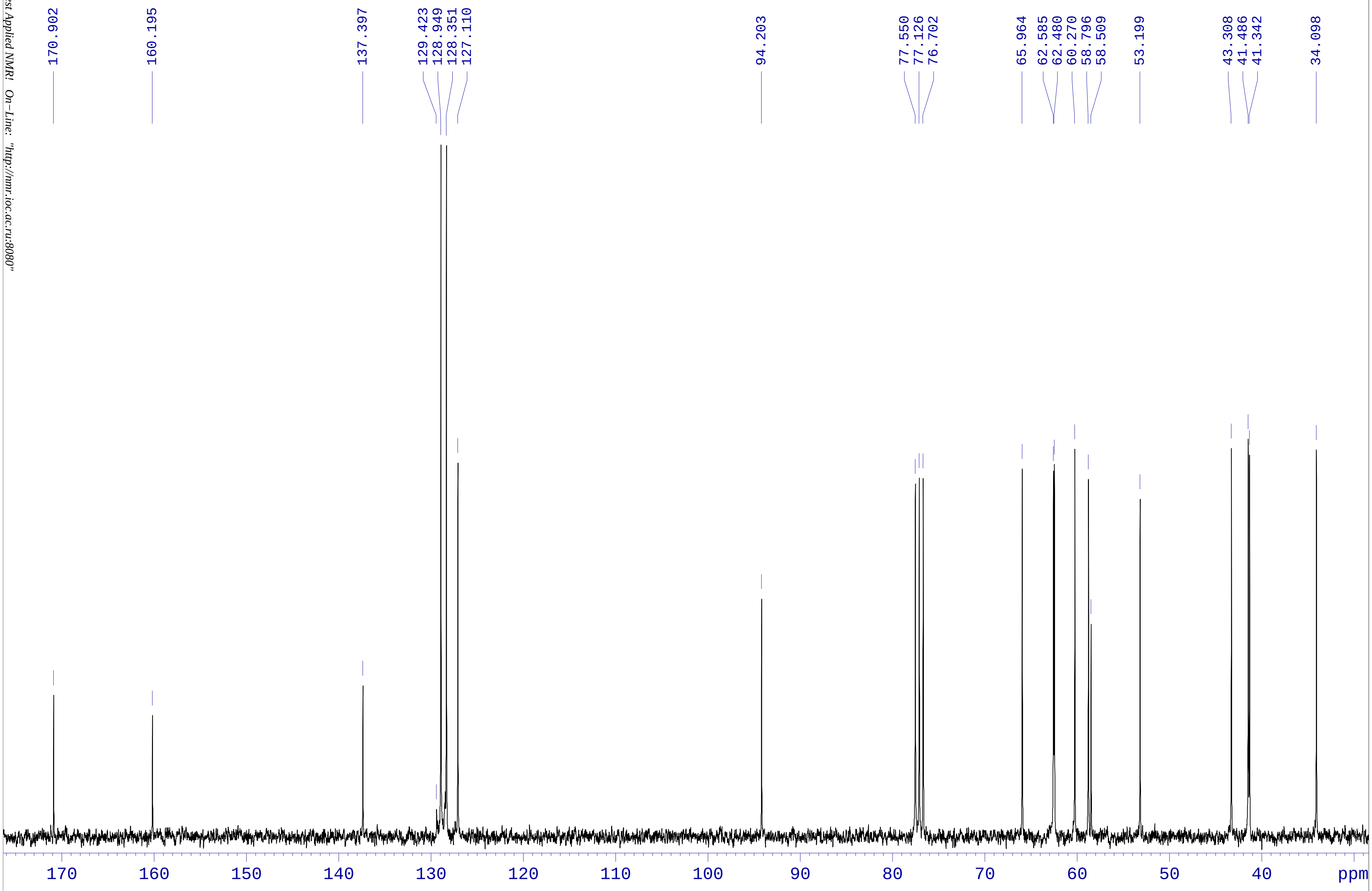

Display Report

Analysis Info

Analysis Name	D:\Data\Kolotyrkina\2021\Bastrakov\0429052.d	Acquisition Date	29.04.2021 18:12:20
Method	tune_50-1600.m	Operator	BDAL@DE
Sample Name	/LPIK AF-432.1	Instrument / Ser#	micrOTOF 10248
Comment	C20H26N4O4S mH 419.1747 clb added CH3OH		

Acquisition Parameter

Source Type	ESI	Ion Polarity	Positive	Set Nebulizer	1.0 Bar
Focus	Not active			Set Dry Heater	200 °C
Scan Begin	50 m/z	Set Capillary	4500 V	Set Dry Gas	4.0 l/min
Scan End	1600 m/z	Set End Plate Offset	-500 V	Set Divert Valve	Waste

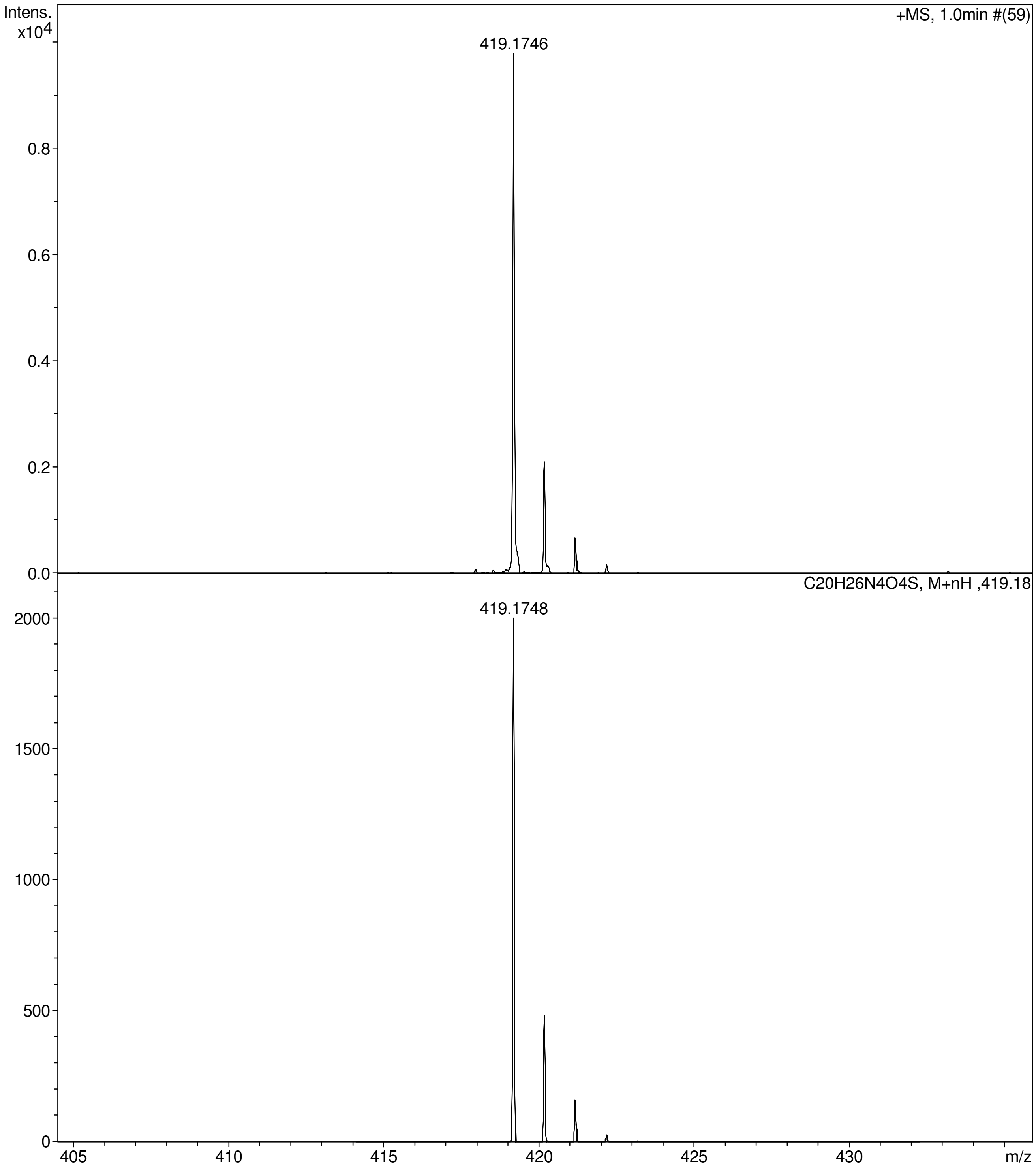

© Zelinsky Institute of Organic Chemistry, Moscow; Bruker AV300 SF=300.13 MHz {1H−1H}COSY SI=1024x256 SW=2242 O1=1302 PW=15.0 AQ=0.237 RD=1.88 NS=1x128 SR=0.00 TE=300K 17 May 2021 Opr: NMR User; Solv: CDCl3;

The Best Applied NMR! On−Line: "http://nmr.ioc.ac.ru:8080"

/LPIK AF−432.1.1 Kokorekin−20259
COSY

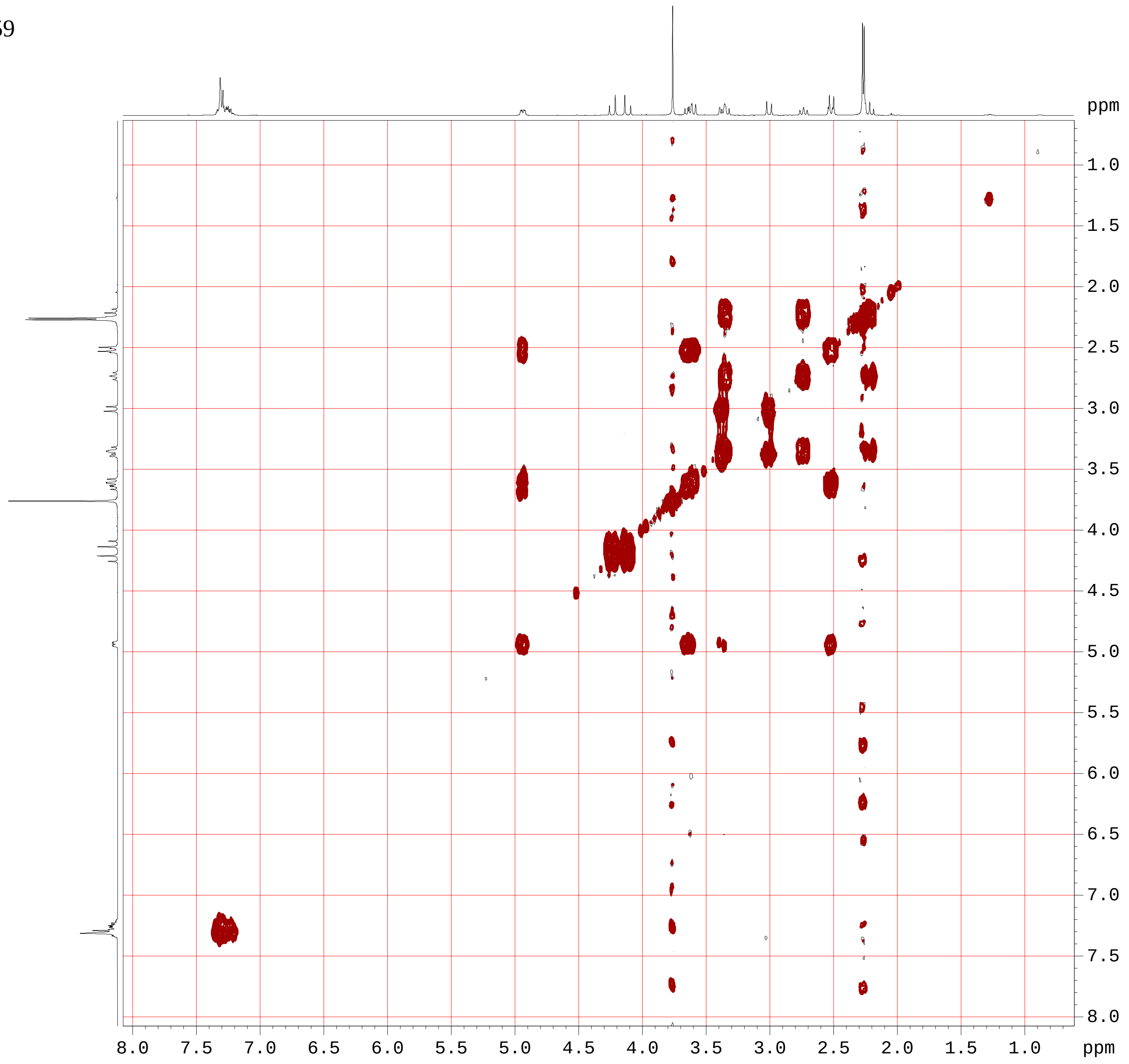

© Zelinsky Institute of Organic Chemistry, Moscow; Bruker AV300 SF=300.13 MHz {1H−1H}COSY SI=1024x256 SW=2242 O1=1302 PW=15.0 AQ=0.237 RD=1.88 NS=1x128 SR=0.00 TE=300K 17 May 2021 Opr: NMR User; Solv: CDCl3;

The Best Applied NMR! On−Line: "http://nmr.ioc.ac.ru:8080"

/LPIK AF−432.1.1 Kokorekin−20259
COSY

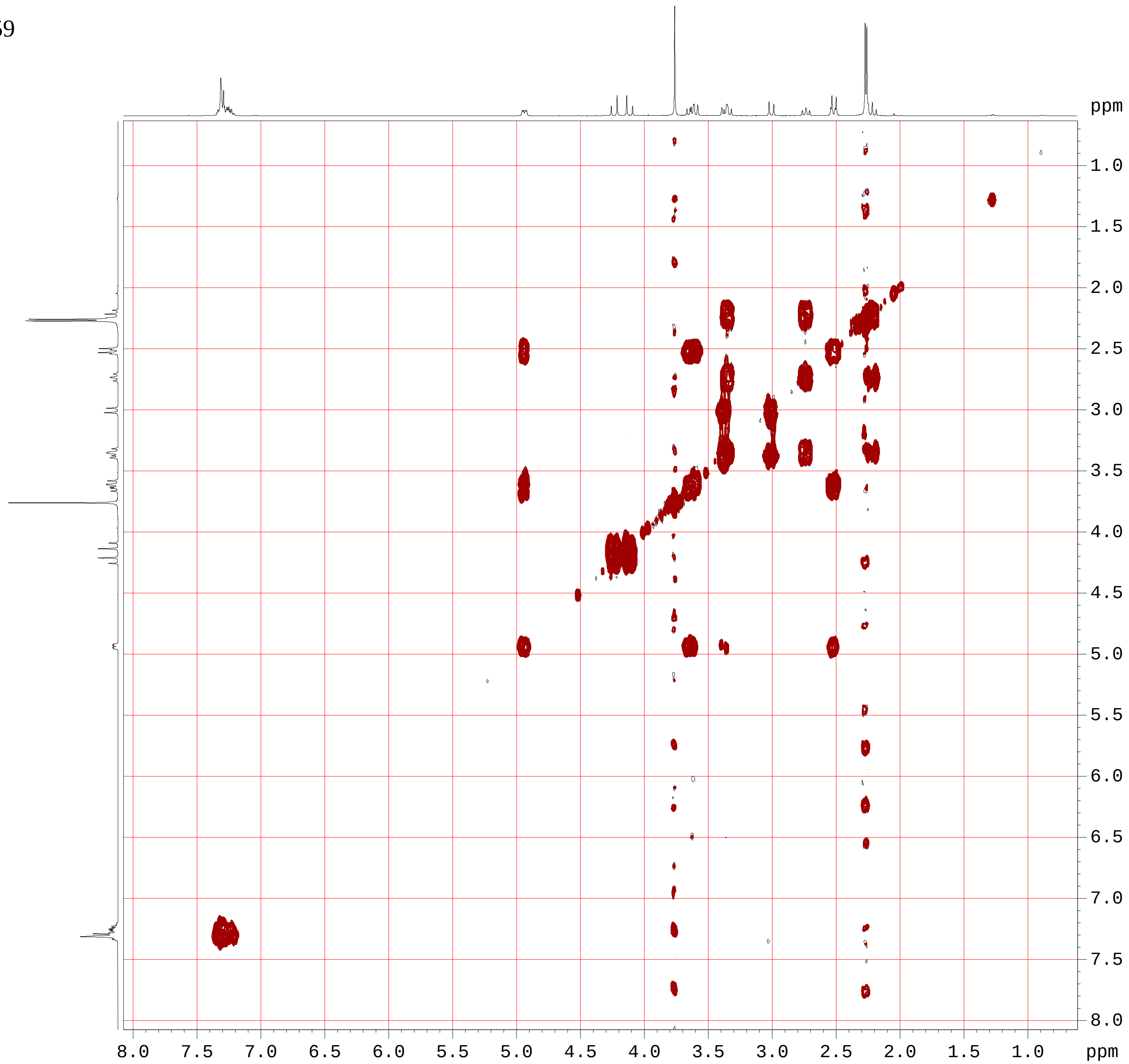

© Zelinsky Institute of Organic Chemistry, Moscow; Bruker AV300 SF=300.13 MHz {1H−13C}HSQC SI=1024x512 SW=2242 O1=1302 PW=15.0 AQ=0.230 RD=1.39 NS=1x256 SR=0.00 TE=300K 17 May 2021 Opr: NMR User; Solv: CDCl3;

The Best Applied NMR! On−Line: "http://nmr.ioc.ac.ru:8080"

/LPIK AF−432.1.1 Kokorekin−20259
HSQC

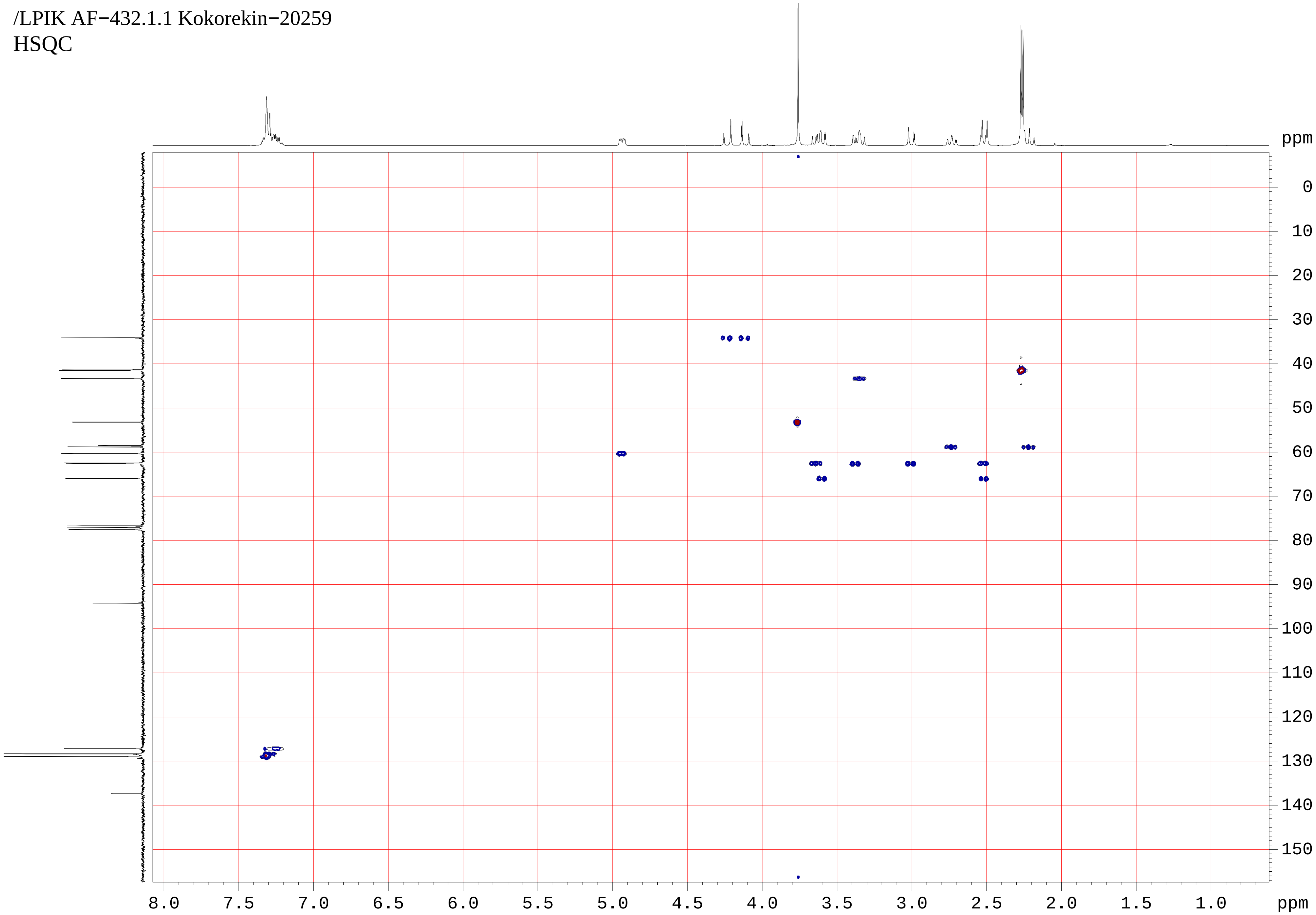

© Zelinsky Institute of Organic Chemistry, Moscow; Bruker AV300 SF=300.13 MHz {1H−1H}NOESY SI=1024x512 SW=2336 O1=1265 PW=15.0 AQ=0.409 RD=1.90 NS=2x256 SR=0.00 TE=300K 19 May 2021 Opr: NMR User; Solv: CDCl3;

The Best Applied NMR! On−Line: "http://nmr.ioc.ac.ru:8080"

/LPIK AF−432.1.NOESY Kokorekin−20259
NOESY

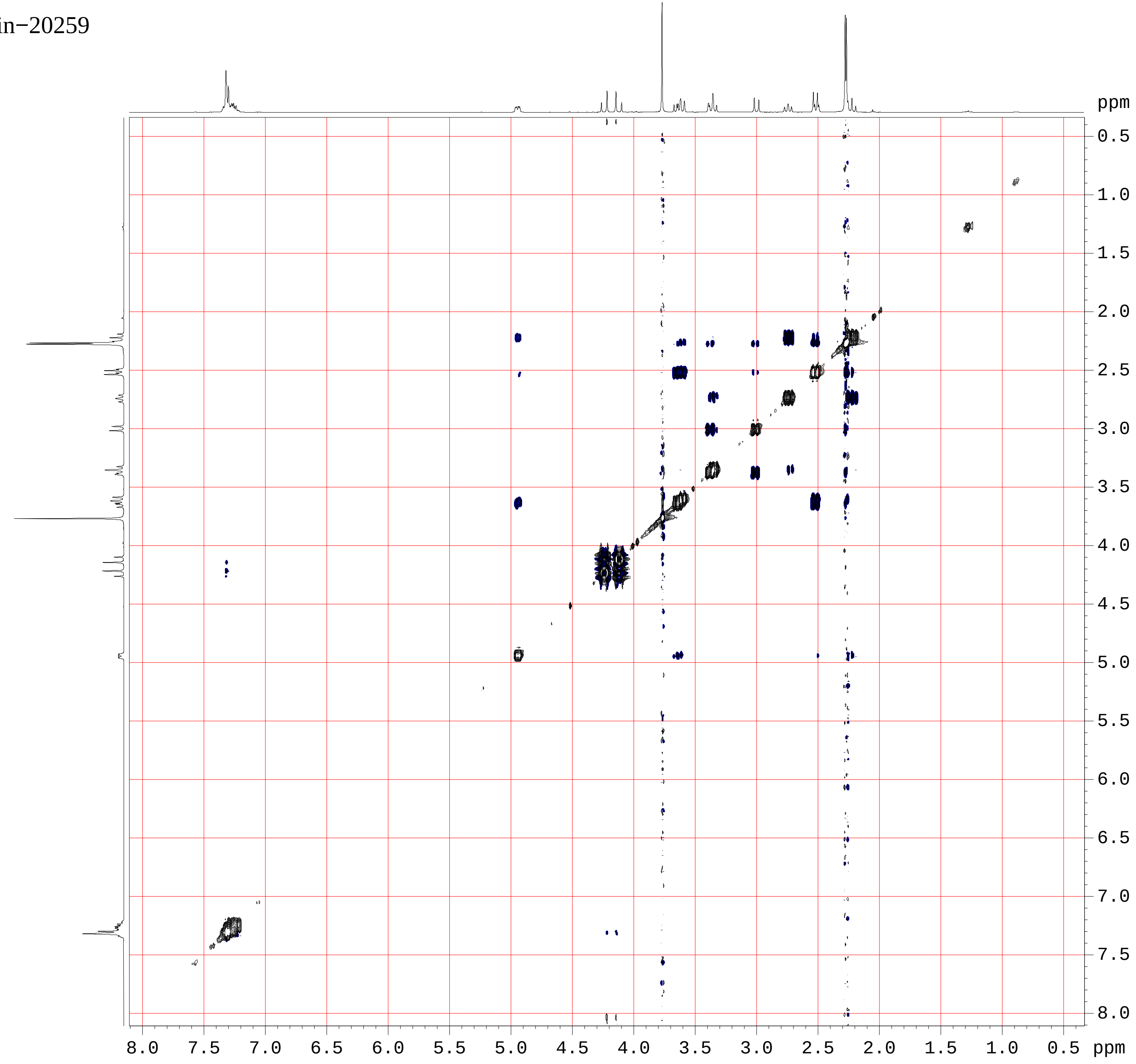

Supplement: Supplementary file 1 [file molecules-26-05547-s001.zip › molecules-1323374-supplementary.pdf]
